# Design, Synthesis and Biochemical Evaluation of Novel Ethanoanthracenes and Related Compounds to Target Burkitt’s Lymphoma

**DOI:** 10.3390/ph13010016

**Published:** 2020-01-17

**Authors:** Andrew J. Byrne, Sandra A. Bright, James P. McKeown, John E. O’Brien, Brendan Twamley, Darren Fayne, D. Clive Williams, Mary J. Meegan

**Affiliations:** 1 School of Pharmacy and Pharmaceutical Sciences, Trinity Biomedical Sciences Institute, Trinity College Dublin, 152-160 Pearse St, Dublin 2, D02 R590, Ireland; byrnea40@tcd.ie (A.J.B.); mckeowjp@tcd.ie (J.P.M.); 2 School of Biochemistry and Immunology, Trinity Biomedical Sciences Institute, Trinity College Dublin, 152-160 Pearse St, Dublin 2, D02 R590, Ireland; brights@tcd.ie (S.A.B.); FAYNED@tcd.ie (D.F.); Clive.Williams@tcd.ie (D.C.W.); 3 School of Chemistry, Trinity Biomedical Sciences Institute, Trinity College Dublin, 152-160 Pearse St, Dublin 2, D02 R590, Ireland; jeobrien@tcd.ie (J.E.O.); TWAMLEYB@tcd.ie (B.T.)

**Keywords:** Burkitt’s lymphoma, DG-75, MUTU-1, anthracene, nitrostyrene, maprotiline, 9,10-dihydro-9,10-ethanoanthracene, apoptosis

## Abstract

Lymphomas (cancers of the lymphatic system) account for 12% of malignant diseases worldwide. Burkitt’s lymphoma (BL) is a rare form of non-Hodgkin’s lymphoma in which the cancer starts in the immune B-cells. We report the synthesis and preliminary studies on the antiproliferative activity of a library of 9,10-dihydro-9,10-ethanoanthracene based compounds structurally related to the antidepressant drug maprotiline against BL cell lines MUTU-1 and DG-75. Structural modifications were achieved by Diels-Alder reaction of the core 9-(2-nitrovinyl)anthracene with number of dienophiles including maleic anhydride, maleimides, acrylonitrile and benzyne. The antiproliferative activity of these compounds was evaluated in BL cell lines EBV^−^ MUTU-1 and EBV^+^ DG-75 (chemoresistant). The most potent compounds **13j**, **15**, **16a**, **16b**, **16c**, **16d** and **19a** displayed IC_50_ values in the range 0.17–0.38 μM against the BL cell line EBV^−^ MUTU-1 and IC_50_ values in the range 0.45–0.78 μM against the chemoresistant BL cell line EBV^+^ DG-75. Compounds **15**, **16b** and **16c** demonstrated potent ROS dependent apoptotic effects on the BL cell lines which were superior to the control drug taxol and showed minimal cytotoxicity to peripheral blood mononuclear cells (PBMCs). The results suggest that this class of compounds merits further investigation as antiproliferative agents for BL.

## 1. Introduction

Burkitt’s lymphoma (BL) is an aggressive non-Hodgkin lymphoma occurring with high incidence in developing areas such as equatorial Africa and Papua New Guinea [1]. Within these tropical regions of high incidence (40–50 per 10^6^), BL accounts for approximately 50% of childhood cancers and up to 90% of diagnosed childhood lymphomas [2]. The treatment of BL in these regions is difficult and the development of new, safe and cost-effective therapeutics are of current interest. For developed countries, the sporadic form represents 1–2% of adult lymphomas [3]. In contrast, the endemic BL is commonly associated with infection by the oncogenic Epstein-Barr virus (EBV), which interrupts cellular pathways regulating cell proliferation and thus prevents apoptosis [4]. Treatment of BL includes the monoclonal antibody rituximab which binds to the cell surface protein CD20 of malignant and normal B lymphocytes [5,6]. Chemotherapy drugs used in combination with rituximab include vincristine, methotrexate, doxorubicin and cyclophosphamide, which result in approximately 90% survival rates in children [7]. However, due to reported development of resistance to chemotherapy drugs and increased incidences of immunodeficient HIV-associated BL [8,9], the development of selective and potent treatments for BL is required.

**Figure 1:**
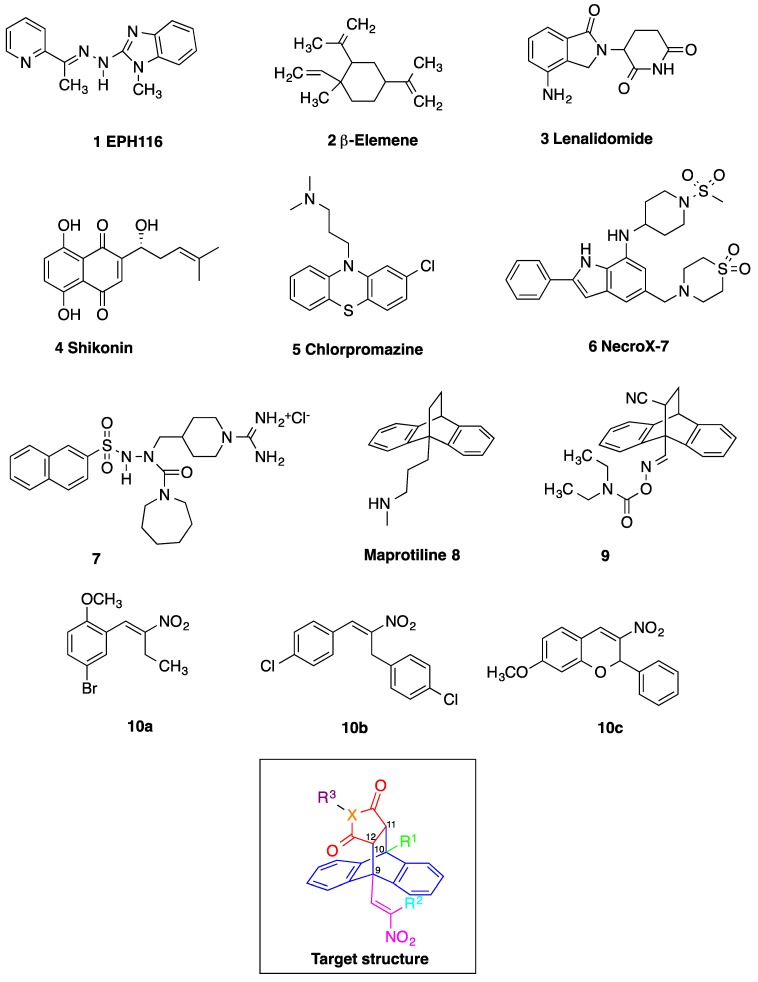
Chemical structures of compounds with reported activity against Burkitt’s lymphoma: compounds **1**–**7**, maprotiline **8**, ethanoanthracene **9** and nitrostyrene lead compounds **10a**–**c** with target ethanoanthracene structure.

Among the compounds with reported biochemical effects in BL are a series of *N*-heteroarylhydrazones (e.g., compound **1**, EPH116) which demonstrated antiproliferative effects and inhibition of ribonucleotide reductase in BL cells [10], Figure 1. The sesquiterpene β-elemene **2** effectively inhibited the growth and induced the apoptosis of BL cells through upregulation of p53 upregulated modulator of apoptosis (PUMA) expression and modulating PUMA related apoptotic signalling pathway [11]. A phase I clinical trial of lenalidomide **3** (G_2_M arrest) and blinatumomab combination in treating patients with relapsed non-Hodgkin lymphoma including relapsed and refractory Burkitt lymphoma is currently in progress [12]. The natural naphthoquinone shikonin **4** suppressed cellular proliferation and induced caspase-dependent apoptosis in BL cells with inhibition of *c-MYC* and suppression of the phosphatidylinositol 3-kinase/protein kinase B/mammalian target of rapamycin (PI3K/AKT/mTOR) pathway [13]. Phenothiazines such as chlorpromazine **5**, trifluoroperazine and thioridazine were noted to both suppress proliferation and induce apoptosis in BL cells [14], while the novel indole based compound NecroX-7 **6** is a reactive oxygen species scavenger and has been shown to induce G_2_/M arrest in BL cell lines [15,16]. Amidinopiperidine-based serine protease inhibitor **7** has been reported as a selective inducer of apoptosis in BL cells [17]. The functional overexpression and the pathogenetic role of the *MYC* proto-oncogene in BL is established [18], indicating the potential role of direct and indirect *MYC* inhibitors as new experimental therapies [19].

Our previous research reported the antidepressant drug maprotiline **8** (Figure 1) as an anti-proliferative and pro-apoptotic agent in BL cell lines MUTU-I and DG-75 [20,21]. The serotonin transporter (SERT) has been identified in B-cell malignancies; subsequently antidepressants and structurally related compounds were investigated for potential antileukemia/antilymphoma activity [22]. Induction of apoptosis was demonstrated by the selective serotonin reuptake inhibitor (SSRI) citalopram and the antidepressants imipramine and clomipramine in HL-60 acute myeloid leukaemia, and human T-lymphocytes [23,24,25]. Although these compounds act as non-selective SERT ligands, the pro-apoptotic activity of these drugs appear to be independent of SERT. In addition, fluoxetine [20,21,22], 3,4-methylenedioxymethamphetamine (MDMA) and analogues [22,26], fenfluramine [22], clomipramine [22] and the norepinephrine transporter (NET) targeting maprotiline and analogues have demonstrated proapoptotic effects in BL cell lines [20,21,27]. Our subsequent work involved the generation of a compound library structurally related to the tetracyclic antidepressant maprotiline. A biological screen of this library identified a number of lead compounds in BL cell lines (MUTU-I and DG-75) [27]. From this study we identified the 9,10-dihydro-9,10-ethanoanthracene scaffold e.g., compound **9** as favourable for anti-proliferative activity in these cell lines while the (*E*)-9-(2-nitrovinyl)anthracene was also shown to elicit a potent pro-apoptotic effect [27]. The ethanoanthracene scaffold was previously reported to demonstrate activity as L-type Ca^2+^ channel blockers [28], glucocorticoid and cannabinoid receptor modulators [29,30], inhibitors of drug efflux in multidrug resistance [31,32] together with antimicrobial [33] antimalarial [34], anti-inflammatory [35] and cytotoxic activity [36].

Nitrostyrenes and related nitrovinyl compounds induce anti-cancer effects and stimulate an apoptotic response in cancer cell lines e.g., oral and colon cancers, osteosarcoma and Erlich ascetic tumour cell lines [37,38,39,40,41,42], modulating tumorigenesis in colon and breast cancer via reactive oxygen species (ROS) mediated pathways [43,44,45]. Inhibition of telomerase, protein tyrosine phosphatase (PTP), phospholipase A2 and tubulin have been demonstrated for simple nitrostyrenes, while antimicrobial, anti-inflammatory and immunosuppressive effects are also reported [37,46,47]. Nitrostyrenes also act as Retinoid X receptor alpha (RXRα) ligands which inhibit tumor necrosis factor alpha (TNFα) activation of nuclear factor kappa B (NF–κΒ) [48]. We have previously investigated the synthesis, characterisation and biochemical evaluation of a series of structurally diverse nitrostyrenes (e.g., compound **10a**, Figure 1) and related nitrovinyl compounds in the MUTU-I and DG-75 cell lines and identified the potent apoptotic effect induced by (*E*)-1,3-bis(aryl)-2-nitro-1-propenes (e.g., compound **10b**, Figure 1) [49] and related heterocycles containing the nitrovinyl pharmacophore e.g., 3-nitro-2-phenyl-2*H*-chromene (**10c**), (Figure 1) with antiproliferative effects superior to the cancer therapeutics fludarabine and taxol [50]. A subset of these lead nitrostyrene compounds were also shown to elicit a potent anti-proliferative and pro-apoptotic response towards a range of malignant cell lines e.g., MCF-7 (ER positive breast cancer), HL-60 (acute promyelocytic leukemia) and HeLa (human cervical cancer) [51]. We have also reported the pro-apoptotic effect of selected examples of these nitrostyrene compounds in chronic lymphocytic leukemia (CLL) cell lines and also in ex vivo CLL patient samples [51].

In the present work, a structurally diverse library of 9,10-dihydro-9,10-ethanoanthracene compounds related in structure to the previous lead nitrostyrene compounds **10a**–**c** and the tetracyclic antidepressant maprotiline **8** were synthesised. This approach will facilitate the identification of potent and selective compounds which may be useful in the design of proapoptotic agents. These compounds are synthesised by Diels-Alder cycloaddition reaction of the required anthracene-diene system and hence modification of the ethano-bridgehead could be achieved by variation of the dienophile employed. The dienophiles chosen for the study included maleic anhydride, maleimide and N-substituted maleimides together with benzyne, acrylate esters and acrylonitrile. Variation of the anthracene substitution at C-9 from nitrovinyl to alternative double bonded systems such as cyanovinyl, imine and oxime are also of interest for biological activity, as is the introduction of maleimide linkers and aryl-substituted maleimides. Structural modifications of the nitrovinyl unit by reduction and extension of the alkyl chain length at C-2 were also explored, (see target structure, Figure 1). The compounds were evaluated in the EBV^−^ MUTU-1 cell line and chemoresistant EBV^+^ DG-75 cell line to establish the structure-activity relationships for these ethanoanthraenes and to optimize the antiproliferative and proapoptotic effects in BL cell lines.

## 2. Chemistry

**Scheme 1:**
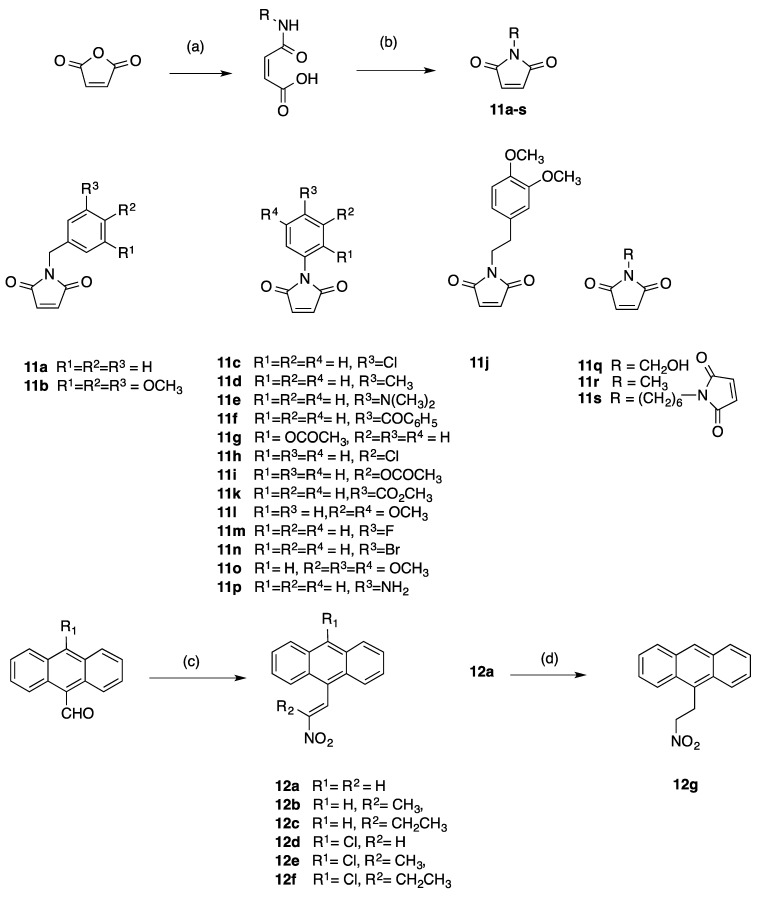
Synthesis of maleimides **11a**–**s** and nitrovinylanthracenes **12a**–**f** and nitroethylanthracene **12g**. Reagents and conditions: (**a**) RNH_2_, diethyl ether, reflux, 1 h. (**b**) NaOAc, Ac_2_O, 120 ^o^C, 30 min. (15–70%); (**c**) Piperidine acetate, excess nitroalkane (CH_3_NO_2_, CH_3_CH_2_NO_2_ or CH_3_CH_2_CH_2_NO_2_), 90 ^o^C, N_2_, 1.5 h. (50–99%); (**d**) NaBH_4_, (CH_3_)_2_CHOH, CH_2_Cl_2_, RT, 24 h, 85%.

The synthesis of the required compounds was achieved by Diels-Alder cycloaddition reaction of the 9-(2-nitrovinyl)anthracene dienes with selected dienophiles. The maleimide-based dienophiles were first prepared by reaction of maleic anhydride with a series of amines (alkyl, benzyl, aryl amines and a diamine) and involved generation of the corresponding amic acid intermediates, (step (a), Scheme 1). Subsequent intramolecular cyclisation of the amic acid with sodium acetate and acetic anhydride, results in the formation of the related maleimide or N-substituted maleimide compounds **11a**–**s**, (step (b), Scheme 1). The (*E*)-9-(2-nitrovinyl)anthracenes **12a**–**f** were prepared by Henry-Knoevenagel condensation of the nitroalkane with various 9-anthraldehydes giving access to a number of diverse diene systems (step (c), Scheme 1). 9-(2-Nitroethyl)anthracene **12g**, was obtained by reduction of **12a** with sodium borohydride, (step (d), Scheme 1). A series of (*E*)-11,12-substiuted-9-(2-nitrovinyl)-9,10-dihydro-9,10-ethanoanthracenes was synthesised by Diels-Alder reaction of the (*E*)-9-(2-nitrovinyl)anthracenes **12a**–**f** and the dienophiles **11a**–**s**.

**Scheme 2:**
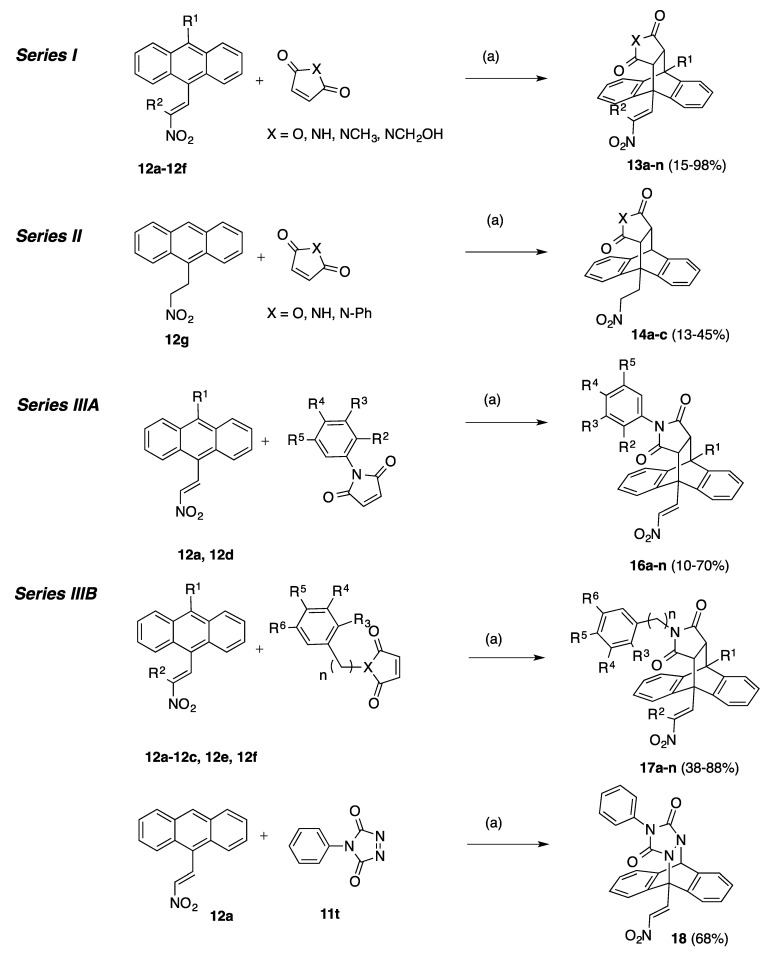
Synthesis of ethanoanthracenes **13a**–**n** (Series I, Table 1), **14a**–**c** (Series II, Table 1), **16a**–**n** (Series IIIA, Table 2), **17a**–**n**, **18** (Series IIIB, Table 3): Reagents and conditions (**a**) Toluene, 90 °C, 48 h.

**Scheme 3:**
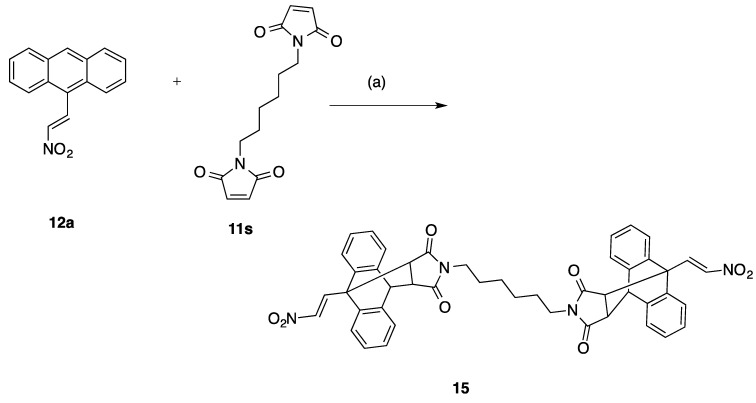
Synthesis of ethanoanthracene dimer **15** (Series 1, Table 1): Reagents and conditions: (**a**) Toluene, 90 °C, 48 h., (10%).

**Scheme 4:**
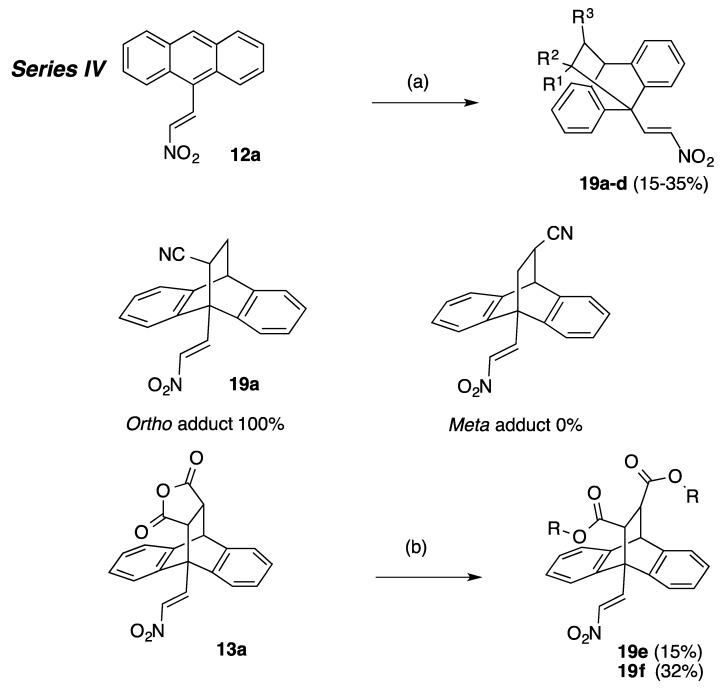
Synthesis of ethanoanthracenes **19a**–**f** (Series IV, Table 6)**.** Reagents and conditions: (**a**) R^1^R^2^C=CHR^3^, (NCC=CH_2_, CH_3_CH_2_OCOC=CH_2_, CH_3_OCOC=CH_2_, CH_3_OCO(CN)C=CH_2_), Toluene, 90 °C, 48 h; (**b**) H_2_SO_4_, R-OH, (R = CH_3_, CH_2_CH_3_), reflux, 6 h.

**Scheme 5:**
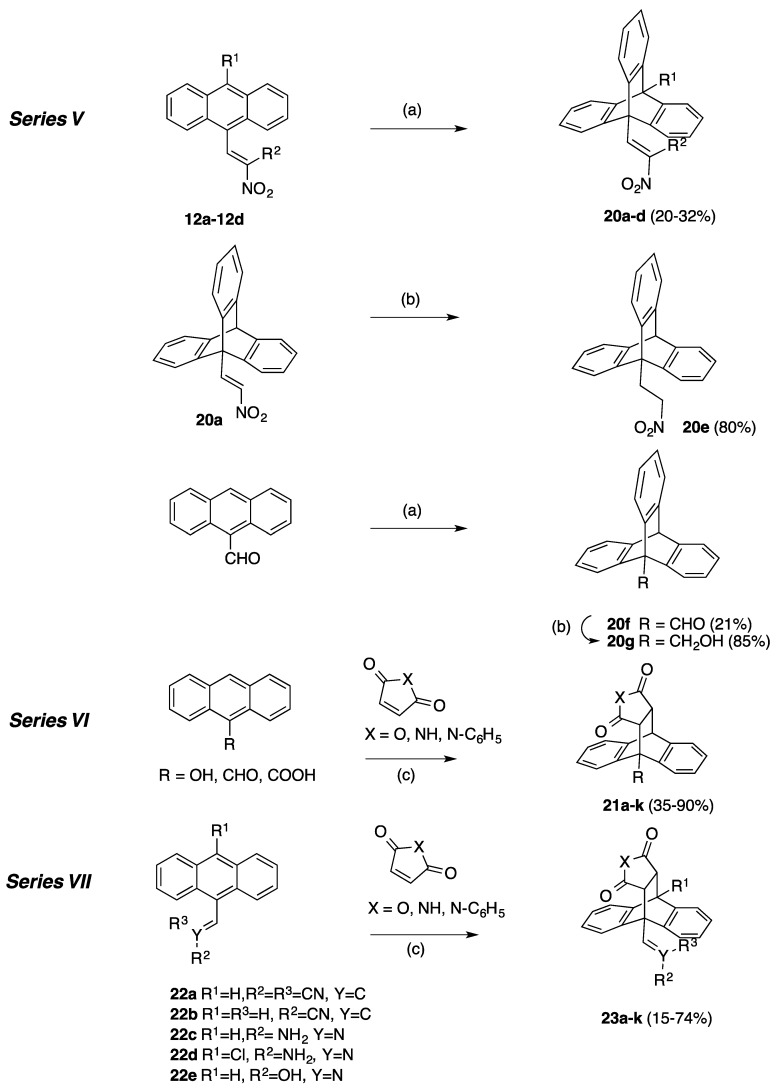
Synthesis of ethanoanthracenes **20a**–**g** (Series V, Table 7), **21a**–**k** (Series VI, Table 8) and **23a**–**k** (Series VII, Table 9). Reagents and conditions: (**a**) Anthranilic acid, CCl_3_CO_2_H, isoamyl nitrite, 0 °C then toluene, 90 °C, 1 h; (**b**) NaBH_4_, (CH_3_)_2_CHOH, CH_2_Cl_2_; (**c**) Toluene, 90 °C, 48 h.

A summary of these Diels-Alder reactions is shown in Scheme 2, Scheme 3, Scheme 4 and Scheme 5. The novel adducts synthesised are arranged as follows:

***Series I (Scheme 2 and Scheme 3, Table 1):*** (*E*)-9-(2-Nitrovinyl)-9,10-dihydro-9,10-ethanoanthracenes **13a**–**n** including maleic anhydride and maleimide Diels-Alder adducts and dimer **15.**

***Series II (Scheme 2, Table 1):*** 9-(2-Nitroethyl)-9,10-dihydro-9,10-ethanoanthracenes **14a**–**c**.

***Series III (Scheme 2, Table 2, Table 3, Table 4 and Table 5):*** (*E*)-9-(2-Nitrovinyl)-9,10-dihydro-9,10-ethanoanthracenes (**16a**–**n**, Series IIIA and **17a**–**n**, Series IIIB) including substituted aryl and benzyl maleimide Diels-Alder adducts and triazole adduct **18**.

***Series IV (Scheme 4, Table 6):*** (*E*)-9-(2-Nitrovinyl)-9,10-dihydro-9,10-ethanoanthracenes **19a**–**f**, substituted at C-11 and C-12.

***Series V (Scheme 5, Table 7):*** (*E*)-9-(2-Nitrovinyl)-9,10-dihydro-9,10-[1,2]benzenoanthracenes **20a**–**g**.

***Series VI (Scheme 5, Table 8):*** 9,10-Dihydro-9,10-ethanoanthracene Diels-Alder adducts **21a**–**k** substituted at C-9.

***Series VII (Scheme 5, Table 9):*** 9,10-Dihydro-9,10-ethanoanthracene Diels-Alder adducts **23a**–**k** containing acrylonitrile, oxime and imine functional groups at C-9.

The compounds **13a**–**n** and **14a**–**c** (Series I and II) encompasses maleic anhydride, maleimide and simple maleimide Diels-Alder adducts of 9-(2-nitrovinyl)anthracenes while the **16a**–**n**, **17a**–**n** products (Series IIIA and IIIB) include substituted phenyl and benzyl maleimide Diels-Alder adducts of 9-(2-nitrovinyl)anthracenes. All products were prepared by cycloaddition reaction of the anthracene dienes with the appropriate dienophile at 90 °C in toluene for 48 h and afforded compounds **13a**–**n** (15–98%), **14a**–**c** (30–45%), **16a**–**n** (10–70%) and **17a**–**n** (38–88%), Scheme 2. Variation of alkyl chain length at the C-2 and C-10 substitution was achieved by utilising different nitroalkanes for the Henry-Knoevenagel condensation with substituted 9-anthraldehydes giving access to a number of diverse diene systems. 9-(2-Nitroethyl)anthracene **12g** was also reacted with maleic anhydride, maleimide and phenyl-maleimide to give the corresponding saturated Diels-Alder adducts (**14a**–**c**), Table 1, Scheme 2. In the ^1^H NMR spectrum of the novel maleic anhydride adduct **13a** the signal at 3.82 ppm appears as a double doublet (*J* = 9.16, 3.66 Hz) and is assigned to H-11 due to interaction with H-10 and H-12 protons which appear as doublets at 4.98 ppm and 4.20 ppm respectively. The doublets occurring at 8.11 ppm and 8.28 ppm (*J* = 14.04 Hz) were assigned to the *trans* coupled protons of the nitrovinyl unit. The assignments were confirmed from the heteronuclear multiple bond correlation (HMBC) and carbon-hydrogen correlation spectroscopy (C-H COSY) NMR spectra, (Supplementary Information). The novel dimer compound **15** was obtained by cycloaddition reaction of (*E*)-9-(2-nitrovinyl)anthracene **12a** and the dimaleimide **11s**, Scheme 3. The ^1^H NMR spectrum of **15** was analogous with the above data for **13a**: the ethano bridge protons at 3.36 ppm (double-doublet), 3.76 ppm (double-doublet) and 4.86–4.92 ppm (multiplet) as expected. High resolution mass spectrometry confirmed the required molecular ion for the dimer 775.2771, C_46_H_39_N_4_O_8_ [M^+^ + H].

The (*E*)-9-(2-nitrovinyl)-13-phenyl-9,10-dihydro-9,10-[3,4]epipyrroloanthracene-12,14-diones Series IIIA (**16a**–**16n**) and Series IIIB (**17a**–**17n**) were prepared from the appropriately substituted (*E*)-9-(2-nitrovinyl)anthracenes via Diels-Alder cycloaddition with substituted benzyl and aryl maleimides with varied aryl substitutions including methoxy, halogen (bromine, chlorine, fluorine), alkyl, hydroxy, ester and amines, (Table 2 and Table 3). The characteristic ^1^H NMR spectrum of compound **16j** shows a double doublet at 3.53 ppm (*J* = 8.55, 3.05 Hz) assigned to H_11_. Doublets occurring at 3.92 ppm (*J* = 8.55 Hz) and 4.95 ppm (*J* = 3.05 Hz) were assigned to H_12_ and H_10_, respectively. The assignments were confirmed from the C-H COSY and DEPT 90 NMR spectra, (See Supplementary Information).

**Figure 2:**
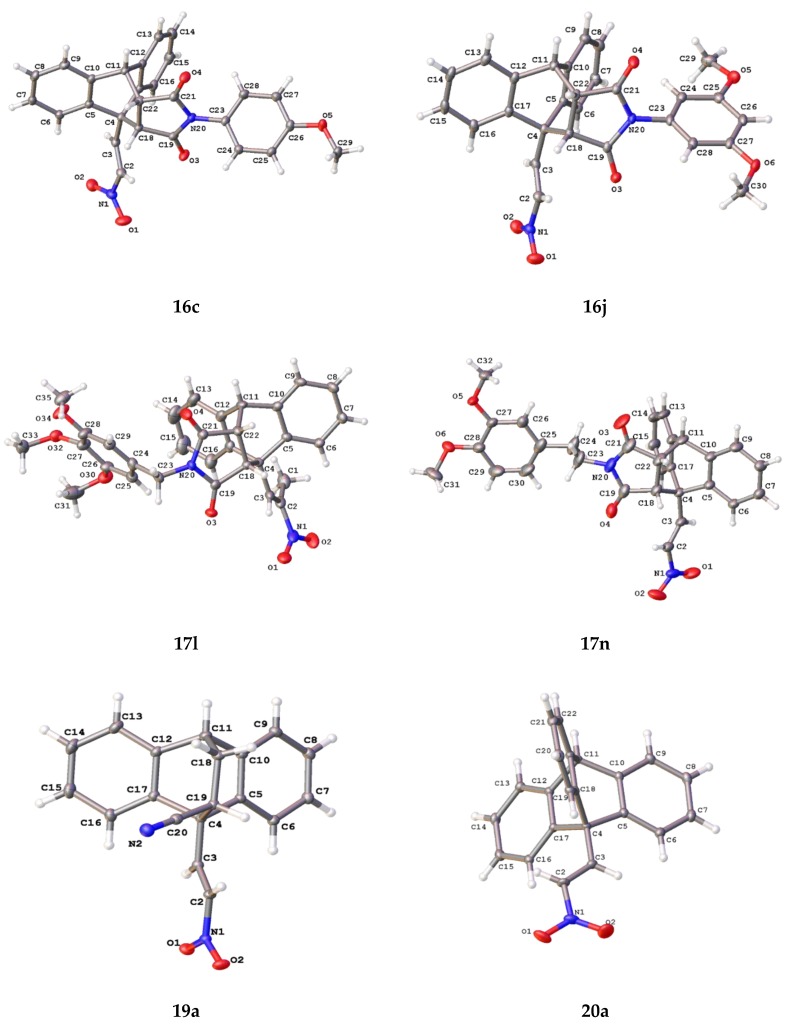
X-ray crystallographic molecular structures of **16c**, **16j**, **17l**, **17n**, **19a** and **20a** with atomic displacement shown at 50% probability. Only major disordered moiety shown for **17l**. Non-hydrogen atoms labelled where possible.

Single crystal X-ray structure determination was completed on (*E*)-9-(2-nitrovinyl)-9,10-dihydro-9,10-[3,4]epipyrroloanthracene-12,14-diones **16c**, **16j**, **17l** and **17n** (Figure 2) and selected data is summarised in Table 4 and Table 5. The core dihydroethanoanthracene moiety is rigid, possessing analogous conformations in the different structures; the packing structure displayed by the products is centrosymmetric and monoclinic. From the asymmetric unit, it was possible to confirm the (*E*)-configuration of the nitrovinyl unit. The angle A^o^ was calculated for each compound as the angle between the centroid of phenyl ring 1 and the centroid of phenyl ring 2—ranging from 122.05° to 129.35°. This angle is 119.46° in the crystal structure of maprotiline [52]. The flat succinimide ring is fused to the ethano bridge, so that it is tilted through almost exactly 60° with respect to the plane of the C- (4)-C(18)-C(22)-C(11) bridging atoms. The angle B^o^ (maleimide substituent centroid to centroid of main axis) was also calculated and ranged from 118.05–121.71°. The distance between the carbons in the ethano bridge (d_1_) ranged from 1.542–1.563 Å, comparing well to that of maprotiline 1.54 Å [52] and related inclusion complexes [53]. The distance between the carbon at C-10 and the nearest carbon of the ethano bridge (d_2_) for the series was 1.555–1.573 Å, while in maprotiline, this distance is 1.546 Å [52].

Reaction of the dienophile 4-phenyl-3*H*-1,2,4-triazole-3,5(4*H*)-dione **11t** with the (*E*)-9-(2-nitrovinyl)anthracene **12a** afforded the adduct **18** in 68% yield (Scheme 2) and allowed direct comparison of the nitrogen-nitrogen bridge system in **18** with the carbon-carbon bridge in compound **16a**. The substituted simple (*E*)-9-(2-nitrovinyl)-9,10-dihydro-9,10-ethanoanthracenes **19a**–**f**, (Series IV) (Scheme 4, Table 6) were prepared from **12a** and the corresponding dienophiles (1-cyanovinyl acetate, methyl acrylate, ethyl acrylate and cyanoacrylate) affording yields of 15–35% (**19a**–**d**) or via a ring opening reaction (**19e**,**f**) in yields of 15–32% (Scheme 4, Table 6). Two products are possible from the Diels-Alder addition of the above dienophiles to the anthracene diene: an ‘ortho’ and a ‘meta’ addition product, (Scheme 4). The Diels-Alder addition of cyanoacrylate to diene systems such as 9-anthraldehyde and (E)-3-(anthracen-9-yl)acrylonitrile has been previously documented as forming the ortho addition adduct only [54]. The preference for the ‘ortho’ adduct is attributed to the stabilising molecular orbital overlap between the nitrile and the carbonyl group. In the present work, we have confirmed the exclusive formation of the novel ortho adduct **19a** from the reaction of acrylonitrile and (E)-9-(2-nitrovinyl)anthracene **12a** using ^1^H NMR and X-ray crystallography, (Table 4, Figure 2). In the ^1^H NMR spectrum of **19a**, the diastereotopic protons H-**12a** and H-**12b** were identified as multiplets at 1.96 ppm and 2.30 ppm. The bridging protons H-11 and H-10 were identified at 3.69 ppm as a double doublet (J = 10.68, 3.97 Hz) and 4.63 ppm as a singlet respectively. Interestingly, although coupling was demonstrated for H-12a with H-10, (J = 2.44 Hz), this is not observed from its singlet signal. The assignments were confirmed from the C-H COSY and Distortionless Enhancement by Polarization Transfer (DEPT 90) NMR spectra, see Supplementary Information. The esters **19e** and **19f** were prepared from the anhydride **13a** using acid catalysed ring opening conditions Scheme 4, [55].

X-ray crystallographic analysis of the novel Diels-Alder adduct (*E*)-10-(2-nitrovinyl)-9,10-dihydro-9,10-ethanoanthracene-11-carbonitrile (**19a**) confirmed the regioisomer obtained, (Figure 2, Table 4 and Table 5), and the packing structure of the product was centrosymmetric and monoclinic. The angle A^o^ between the centroid of phenyl ring 1 and the centroid of phenyl ring 2 was determined as 126.94^o^. This angle is reported as 119.46^o^ in the crystal structure of maprotiline [52]. The distance between the carbons in the ethano bridge (d_1_) was 1.565 Å, comparing well to that of maprotiline 1.540 Å. The distance between the carbon at C-10 and the nearest carbon of the ethano bridge (d_2_) was 1.556 Å, in maprotiline this distance is 1.546 Å [52].

To investigate the effect of increased rigidity on the ethanoanthracene bridge, the (*E*)-9-(2-nitrovinyl)anthracenes (**12a**–**12d**) were converted to the corresponding (*E*)-9-(2-nitrovinyl)-9,10-dihydro-9,10-[1,2]benzenoanthracenes (**20a**–**d**) in modest yields (Scheme 5, Series V, Table 7). The Diels-Alder reaction was achieved with benzyne as the dienophile generated in situ by thermal decomposition of benzenediazonium-2-carboxylate, (prepared from anthranilic acid and isoamyl nitrite) [56,57,58,59,60]. Diphenyl and triphenyl side products have been reported using in situ benzyne generation [57], but these were not isolated during the current work. The benzyne adduct **20f** was similarly prepared from anthracene carboxaldehyde. Sodium borohydride reduction of **20a** and **20f** afforded the alcohol and nitroalkane analogues **20e** and **20g,** respectively. The library of novel triptycene analogues synthesised are summarised in Table 7. The ^1^H NMR spectrum of compound **20d** shows two multiplets occurring at 7.58–7.69 ppm and 7.72–7.82 ppm both integrating for three protons each. These signals were assigned to the two groups of three equivalent aromatic protons H-1, H-8, H-3″ and H-4, H-5, H-6″ respectively. The assignments were confirmed from the C-H COSY, HMBC and DEPT 90 NMR spectra, (see Supplementary Information).

The structure of **20a** was confirmed by X-ray crystallographic analysis (Figure 2, Table 4 and Table 5), showing that the packing structure assumed by the product was monoclinic and confirming the (*E*)-configuration of the nitrovinyl unit. The angles between the centroids of each of the three phenyl rings and the centroid of the central axis were calculated from the asymmetric unit, these were A^o^ (118.50^o^), B^o^ (121.24^o^) and C^o^ (120.18^o^). This angle is 119.46^o^ in the crystal structure of maprotiline. The distance between the carbons in the ethano bridge (d_1_) was 1.400 Å, in maprotiline this distance is 1.540 Å [52]. The distance between the carbon at C-10 and the nearest carbon of the ethano bridge (d_2_) was 1.518 Å, in maprotiline this distance is 1.546 Å [52], hence the X-ray structures indicate a comparable configuration for **20a** and maprotiline.

Diels-Alder cycloaddition reactions with substituted anthracenes using maleic anhydride and maleimide dienophiles were further investigated to determine how the replacement of the nitrovinyl group by other functionalities at C-9 would affect the potency of the products. The 9-substituted Diels-Alder adducts **21a**–**k** were obtained using with diene systems anthracene carboxylic acid, anthraldehyde and anthrone in yields of 35–90%, (Series VI, Scheme 5, Table 8). Similar Diels-Alder reaction with anthracene malononitrile (**22a**), acrylonitrile (**22b**) [27,61], hydrazines (**22c**, **22d**) and oxime (**22e**) dienes afforded the cycloaddition products **23a**–**k** in yields of 15–74%, (Series VII, Scheme 5, Table 9). We now report the first direct Diels-Alder reaction of (*E*)-(anthracen-9-ylmethylene)hydrazine (**22c**) and (*E*)-anthracene-9-carbaldehyde oxime (**22e**) with dienophiles maleic anhydride, maleimide and N-phenylmaleimide. Previous routes to these compounds required the reaction of Diels-Alder adducts of 9-anthraldehyde (e.g., **20f**) with hydroxylamine [27,62,63,64] or hydrazine [65,66]. In the current work, the synthesis of the novel 2-(anthracen-9-ylmethylene)malononitrile Diels-Alder adducts **23d**–**f** was also achieved. In the ^1^H NMR spectrum of the hydrazine **23g** the characteristic signals for H-12 and H-11 were identified as doublets at 3.23 ppm and 3.44 ppm (*J* = 8.55 Hz) while the singlet at 4.72 ppm accounted for H-9, (see Supplementary Information).

A preliminary stability study of the representative ethanoanthracene compound **16a** was carried out at acidic, neutral and basic conditions (pH 4, 7.4 and 9) using HPLC. The half-life (t_½_) was determined to be 11 h at pH 4, 10.5 h at pH 7.4 and greater than 24 h at pH 9. Based on this stability study the compound would be suitable for further preclinical investigation.

## 3. Biochemistry

### 3.1. Preliminary Evaluation of In Vitro Anti-Proliferative Activity of the Ethanoanthracenes in Burkitt Lymphoma EBV^−^MUTU-1 and EBV^+^DG-75 (Chemoresistant) Cell Lines

The panel of compounds synthesised (Series I–VII) based on the 9,10-dihydro-9,10-ethanoanthracene scaffold was then initially screened at two concentrations (1 μM and 10 μM) for antiproliferative activity in the BL EBV^−^MUTU-1 and EBV^+^DG-75 (chemoresistant) cell lines to determine the structure-activity relationship for these maprotiline analogues. The results obtained from this preliminary screen in the MUTU-I and DG-75 cell lines (at 10 µM and 1 µM) are displayed in Table 1, Table 2 and Table 3 and Table 6, Table 7, Table 8 and Table 9, with maprotiline and taxol used as the control drugs. Maprotiline induced a modest anti-proliferative effect at 10 µM in the MUTU-I and DG-75 BL cell lines, while taxol was more effective with 10% cell viability at 10 μM in both cell lines. The results obtained for these novel ethanoanthracene compounds (Series I-VII) are discussed by structural type.

#### 3.1.1. Series I and II, Compounds **13a**–**n**, **14a**–**c** and **15**

The initial lead 9-(2-nitrovinyl)anthracene **12a** demonstrated activity (< 14% cell viability) in both BL cell lines at 10 µM, (Table 1). The effects of the maleic anhydride adducts **13a**–**f** and maleimide adducts (**13g**–**n**) of the lead nitrovinyl anthracene compound **12a** on cell viability were first investigated (Table 1). The 9-(2-nitrovinyl)-9,10-dihydro-9,10-ethanoanthracenes **13a**, **13b** produced a significant anti-proliferative effect at both 1 and 10 μM concentrations in the MUTU-I cell line (7–24% cell viability), (Table 1). In the DG-75 cell line the maleic anhydride derivative **13a** gave slightly improved results compared to maprotiline at both concentrations. C-10 chloro substitution e.g., **13d** resulted in decreased activity in both cell lines. The anhydride **13b** and the imide **13g** demonstrated potent antiproliferative effects in DG-75 cell line (1% cell viability at 1 μM), with the imide **13g** more potent than the anhydride **13a**. The 9-chlorosubstituted imide **13j**, N-methylimide **13m** and alcohol **13n** demonstrated potent antiproliferative effects in the MUTU-1 cell line (< 1% cells remaining at 1 μM). Increasing alkyl chain length at the C-2 position decreased the anti-proliferative effect across most examples of the series (e.g., **13f**, **13i**, **13l**). Reduction of the C-9 nitrostyrenes **13a**, **13g** to afford the C-9 nitroalkane substituted **14a** and **14b** (Series II) resulted in significant reduction in activity, indicating the essential requirement of the nitrostyrene functional group for activity. The dimer compound **15** was identified as a potential lead compound with significant activity in both cell lines (< 5% viable cells at 1 μM).

#### 3.1.2. Series IIIA, Compounds **16a**–**n**

Compounds **16a**–**n** (Series IIIA, Table 2) were designed to investigate the effect of N-arylsubstitution (halogen, ether, phenol, ketone, ester, amine) on antiproliferative activity of the lead ethanoanthracene scaffold structure **13a**. All analogues in the series elicited a very potent anti-proliferative action in the MUTU-I cell line at 1 µM (<5% cell viability, except **16a**, 10%). In the DG-75 cell line, the most potent activity was produced by compounds **16a** and **16b**, with <6% viable cells remaining at 1 µM. The 9-chloro compound **16d** also produced a promising result at 1 µM with <14% cell viability. Significantly reduced activity was observed when comparing compounds **16b** (*p*-Cl) and **16k** (*m*-Cl) in the DG-75 cell line. In a study of para phenyl substitution of this series of compounds **16a**–**16n** in the DG-75 cell line, the unsubstituted compound **16a** and p-chloro compound **16b** were found to elicit more favourable antiproliferative effects than the bromo (**16g**), fluoro (**16e**), amino (**16n**), ketone (**16m**) and methoxy (**16c**) analogues (Table 2). Maleimide (**13g**) and phenyl maleimide (**16a**) adducts produce superior activity in the two BL cell lines than the maleic anhydride adduct **13a**. C-9 chloro substituted compounds **13j** and **16d** possessed significant anticancer effects in both cell lines (0.4–13.5% cell viability).

#### 3.1.3. Series IIIB, Compounds **17a**–**n**

Compounds **17a**–**n** were designed to investigate the effect of the alkyl substitution on the nitrostyrene group, and also to investigate the introduction of a benzyl or phenylethylamine substitution on the heterocyclic nitrogen (Table 3). The deactivating effect of extended alkyl chain length (methyl and ethyl) at C-2 on anticancer activity can once again be observed (70–90% cell viability). With the exception of compounds **17i** (N-benzyl) and **17n** (N-phenylethylamine), these analogues elicited poor anti-proliferative action in both MUTU-I and DG-75 cell lines confirming that alkyl substitution (methyl or ethyl) on the nitrostyrene group dramatically reduces activity e.g., comparing adducts **16a** with **17a** and **17b** in both DG-75 and MUTU-I cell lines at 10 µM, (Table 3). Compound **18** was less potent at the lower concentration in both BL lines than **16a**, indicating that the ethanoanthracene structure was more favourable than the triazole-anthracene bridged system for the desired anticancer effect.

#### 3.1.4. Series IV, Compounds **19a**–**f**

The effect of ethano-bridge substitution on antiproliferative effects was assessed in compounds **19a**–**f** using a range of different dienophiles for the Diels-Alder reaction, together with the ring opening reaction of the anhydride **13a** to introduce nitrile and ester groups on the ethano-bridge, Table 6. All but one of these simpler Diel-Alder adducts possessed enhanced anti-proliferative activity compared to the parent nitrovinylanthracene compound **12a**. The most promising compounds identified from this cohort were **19a**, **19c** and **19f** showing good anti-proliferative effects in both cell lines with cell viability of 0–14% at 10 μM. The inclusion of the ester group on the ethano-bridged system in **19d** reduced the potency observed for compound **19a**, (Table 6).

#### 3.1.5. Series V, Compounds **20a**–**g**

A series of triptycene based Diel-Alder adducts were prepared to assess the effect of the rigid triptycene moiety on the anti-proliferative activity (Table 7). The C-9 functionalities such as nitrovinyl **20a**, **20b**, **20c**, **20d**, nitroalkane **20e**, aldehyde **20f** and alcohol **20g** were included to assess their impact on the anticancer properties of the series. The most promising results were obtained by nitrovinyl compounds **20a** and **20d** (10-chloro) having potent effects (<6% cell viability) in the MUTU-I cell line. A potent effect was also observed for **20d** in DG-75 at 10 µM with no viable cells detected. Reduction of the nitrovinyl bond was once again detrimental to anti-proliferative effects in both cell lines (e.g., **20e**). Alkyl substituent at C-2 of the nitrovinyl unit resulted in diminished activity demonstrated for methyl (**20b**) and ethyl (**20c**) compounds. The aldehyde based triptycene (**20f**) exhibited good activity at the higher concentration in the MUTU-I cell line (<5%), while alcohol (**20g**) was inactive (Table 7).

#### 3.1.6. Series VI, Compounds **21a**–**21k**

The 9,10-dihydro-9,10-ethanoanthracenes **21a**–**k** which do not contain the nitrovinyl unit at C-9 demonstrated greatly reduced antiproliferative activity in both cell lines (>60% viability), indicating the C-9 nitrovinyl substitution was essential for enhanced anti-proliferative effect, Table 8. The 9,10-dihydro-9,10-ethanoanthracenes assessed include C-9 unsubstituted **21a**–**c**, C-9 substituted aldehydes **21d**–**f**, alcohols **21j**,**k** and acids **21g**–**i**, Table 8. Although these compounds possess anticancer activity exceeding the activity of maprotiline at 1 µM in the DG-75 cell line, the antiproliferative effect in the MUTU-I cell line was minimal (>80% cell viability).

#### 3.1.7. Series VII, Compounds **23a**–**k**

A group of C-9 substituted 9,10-dihydro-9,10-ethanoanthracenes **23a**–**k** derived from maleic anhydride, maleimide and phenyl maleimide were next investigated for antiproliferative activity, to assess the effect of variation of the C-9 unsaturated substituent, Table 9. Of the C-9 acrylonitrile compounds **23a**–**f**, the most promising was identified as compound **23b** which demonstrated activities comparable to maprotiline in the MUTU-I (79% cell viability) and DG-75 (54% cell viability) cell lines, Table 9. Further C-9 substituted 9,10-dihydro-9,10-ethanoanthracenes included hydrazones (**23g**–**i**) and oximes (**23j**,**k**). The most potent compound in the DG-75 cell line was identified as the hydrazone **23i**, (19% cell viability). In summary, introduction of alternative double bond functionality to replace the nitrovinyl e.g., cyanovinyl (**23a**–**f**), oxime (**23j**,**k**) and hydrazone (**23g**–**i**) were found to reduce the observed antiproliferative effects when compared with the C-9 nitrovinyl compounds **13a**, **13f** and **16a** respectively, (Table 9).

In this initial evaluation of the ethanoanthracenes (Series I–VII) for antiproliferative activity, a number of key structural requirements were identified. The nitrovinyl pharmacophore was demonstrated to be critical for antiproliferative effect, epipyrrolo bridgeheads were also found to exert a more potent effect than simple ethano and furan-based bridgeheads. Introduction of a chloro substituent at C-9 of the anthracene core also contributed to potency for many compounds.

### 3.2. In Vitro Anti-Proliferative Activity of Selected Potent Ethanoanthracenes

Based on the results obtained from the cell viability study above, the following potent compounds were identified for further investigation in the MUTU-I cell line: maleimide **13j**, N-hydroxymethylmaleimide **13n**, and maleimide dimer **15** (Series I), N-arylmaleimides **16a**–**16j**, **16m**, **16n** (Series IIIA), N-benzylmaleimide **17n** (series IIIB), acrylonitrile adduct **19a**, acrylate ester adduct **19c** and diester **19f** (Series IV) and triptycene **20d** (Series V). The IC_50_ values were determined in the sub-micromolar range (0.09–0.55 μM), with compound **15** identified as the most potent (IC_50_ = 0.09 μM), Table 10. The MUTU-1 IC_50_ values combined with the data provided by the preliminary screen in the DG-75 cell line were used to select the following compounds for subsequent IC_50_ determination in the chemoresistant DG-75 cell line: **13j**, **15** (Series I), **16a**, **16b**, **16c**, **16d** (Series II) and **19a** (Series IV), Table 11.

The IC_50_ values of all the selected compounds **13j**, **15**, **16a**–**d** and **19a** were not only more potent than the lead 9-nitrovinylanthracene based compound **12a** (MUTU-I; IC_50_ 2.57 µM, DG-75; 2.08 µM) and maprotiline (MUTU-I; 15.8 µM, DG-75; 37.5 µM) in both BL cell lines but also these compounds exerted a more potent effect than taxol (MUTU-I; 0.32 µM, DG-75; 1.32 µM) with submicromolar IC_50_ values of 0.09–0.38 µM in the MUTU-I cell line and 0.24–0.78 µM in the chemoresistant DG-75 cell line. (Table 11). These novel compounds were selected for further investigation also based on analysis of their drug-like properties (Lipinski) from a Tier-1 profiling screen, together with predictions of blood brain barrier partition, permeability, plasma protein binding, metabolic stability and human intestinal absorption properties which confirmed that these compounds are moderately lipophilic-hydrophilic drugs and are suitable candidates for further investigation (Tables S1 and S2, Supporting information). Compounds **12a**, **13j**, **16a**–**d** and **19a** were found to satisfy all the Lipinski rule of five criteria with logP values in the range 3.31–5.26, indicating their potential as lead compounds for further development. The dimeric compound **15** was identified as the most potent analogue evaluated with an IC_50_ value of 0.09 µM in the MUTU-I cell line and an IC_50_ of 0.24 µM in the DG-75 cell line.

**Figure 3:**
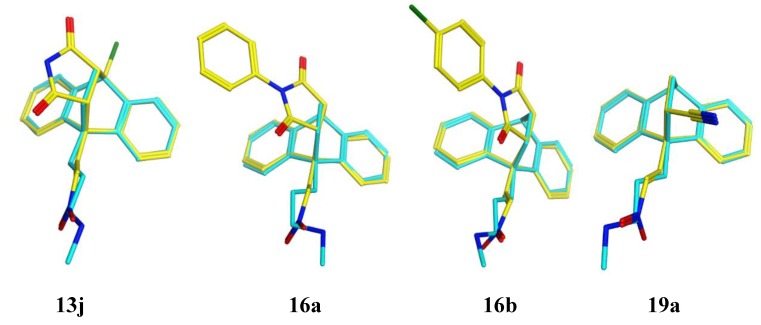
Overlay of 9,10-dihydroethanoanthracene compounds **13j**, **16a**, **16b** and **19a** (yellow) with maprotiline **8** (cyan). The atoms are coloured by element type: oxygen = red, nitrogen = blue, chlorine = green.

Examples of the potent compounds **13j**, **16a**, **16b** and **19a** (displayed as yellow in their respective overlays) were flexibly aligned with the lead compound maprotiline **8** (cyan) using MOE (Molecular Operating Environment) 2016.V8, (Figure 3). The close correspondence between overlays of these compounds with maprotiline highlights the presence of three main shared molecular features: the (*E*)-configuration nitrovinyl pharmacophore located at C-9, the 9,10-dihydroanthracene core structure and the presence of the 9,10-ethanoanthracene bridge, unsubstituted as in maprotiline, having a nitrile substituent as in the acrylonitrile adduct **19a** or forming part of the heterocyclic structure as in the maleimide adducts **13j**, **16a** and **16b**.

### 3.3. Investigations into the Pro-Apoptotic Effect of the Most Potent Ethanoanthracenes-FITC Annexin V/PI FACS Analysis

**Figure 4:**
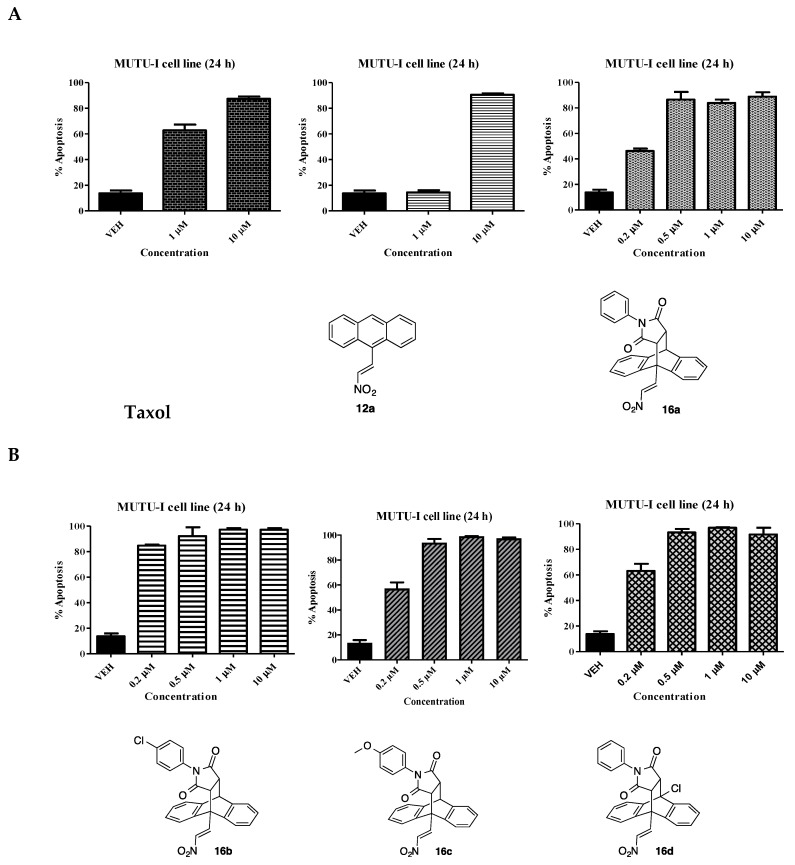

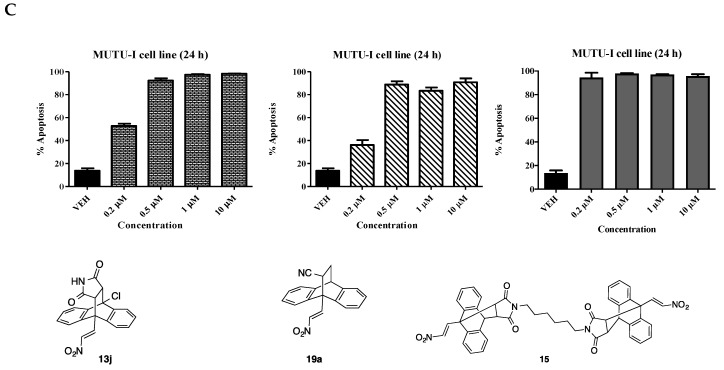
Ethanoanthracene nitrostyrene compounds **16a**–**d**, **13j**, **19a**, **15** induce apoptosis in Burkitt’s lymphoma MUTU-1 cell line. Induction of apoptosis in Burkitt’s lymphoma MUTU-1 cell line following treatment for 24 h with selected ethanoanthracene nitrostyrene compounds (0.2–10 μM) or a comparative control (taxol) in MUTU-1 cell line is determined using Annexin V and propidium iodide staining/FACS. (**A**) Taxol, **12**, **16a**; (**B**) **16b**, **16c**, **16d**; (**C**) **13j**, **19a**, **15**. Ethanol was used as the vehicle. Values represent the mean of three independent experiments.

The potential pro-apoptotic effects of the most potent maprotiline analogues were determined by Annexin V/PI FACS analysis of a subset of the most potent compounds identified e.g., compounds **12a**, **13j**, **15**, **16a**–**d** and **19a**. The study was carried out over a concentration range (0.2–10 µM) in both the BL cell lines (DG-75 and MUTU-I), Figure 4. Taxol was used as a positive control. Taxol was found to elicit a pro-apoptotic effect in the MUTU-I at both 10 µM (87%) and 1 µM (63%). The initial anthracene-nitrostyrene compound **12a** demonstrated potent apoptotic activity at 10 µM (90%), with little effect at 1 µM [27] (Figure 4A). Compound **16a** induced over 80% apoptosis at 10 µM, 1 µM and 0.5 µM with 46% apoptosis at 0.2 µM (Figure 4A). The *p*-chlorophenyl compound **16b** induced a more potent effect than the unsubstituted compound **16a** with a response of >80% apoptosis at all concentrations in the MUTU-I cell line. The *p*-methoxyphenyl compound **16c** induced >90% apoptosis at 0.5 µM. Similar activity was observed for the 10-chloro compound **16d** with 63% apoptosis at 0.2 µM, (Figure 4B). Compounds **13j** and **19a** were also found to possess potent apoptotic activity at 0.5 µM > 90%. The most potent compound investigated was the dimer **15**, demonstrating > 90% apoptosis at 0.2 μM, (Figure 4C). In summary, compounds **15** and **16b** not only possessed the most potent antiproliferative activity but were found to induce the most favourable pro-apoptotic response in the MUTU-I cell line.

**Figure 5:**
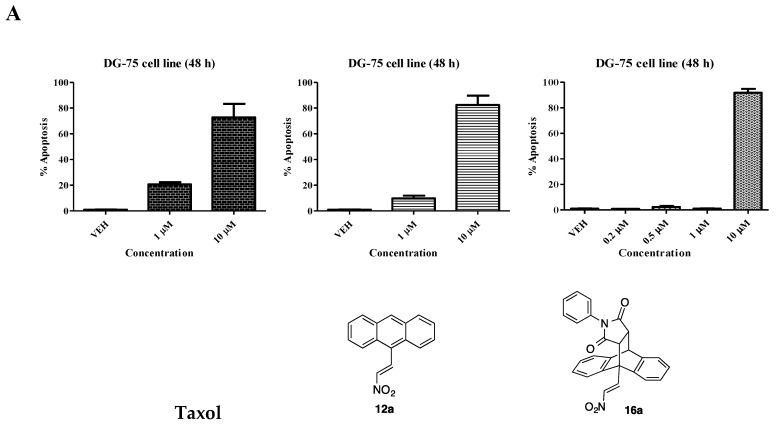

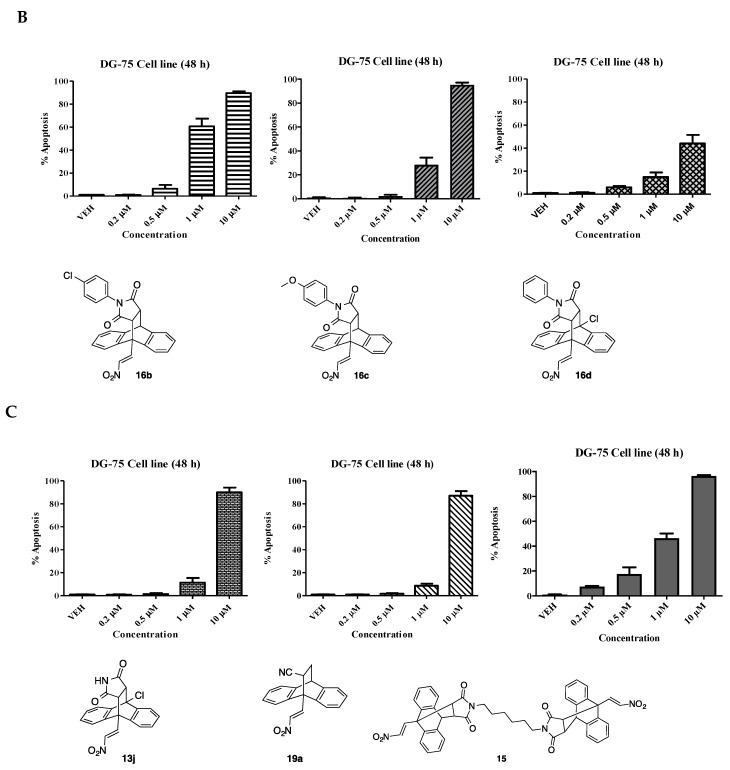
Ethanoanthracene nitrostyrene compounds **16a**–**d**, **13j**, **19a**, **15** induce apoptosis in Burkitt’s lymphoma DG-75 cell line. Induction of apoptosis in Burkitt’s lymphoma DG-75 cell line following treatment for 48 h with selected ethanoanthracene nitrostyrene compounds (0.2–10 μM) or a comparative control (taxol) in DG-75 cell line is determined by Annexin V and propidium iodide staining/FACS: (**A**) Taxol, **12**, **16a**; (**B**) **16b**, **16c**, **16d**; (**C**) **13j**, **19a**, **15**. Ethanol was used as the vehicle. Values represent the mean of three independent experiments.

In the chemoresistant DG-75 cell line, taxol was found to elicit a pro-apoptotic effect at both 10 µM (73%) and 1 µM (21%), (Figure 5A). Compound **12a** elicits similar effects to taxol at both 10 µM (82%) and 1 µM (10%). The unsubstituted maleimide adduct **16a** shows a potent apoptotic effect at 10 µM (92%) but is inactive at lower concentrations (<5% apoptosis). The *p-*chlorophenylmaleimide **16b** and *p*-methoxyphenylmaleimide compound **16c** exhibits improved pro-apoptotic activity when compared to the unsubstituted compound **16a** in the DG-75 cell line. Compound **16b** and **16c** induce 61% and 29% apoptosis at 1 µM respectively. The 10-chloro compound **16d** induced 44% apoptosis at 10 µM and a modest 15% at 1 µM (Figure 5B). The maleimide Diels-Alder adduct **13j** and acrylonitrile adduct **19a** induced 90% and 87% apoptosis at 10 µM. The leading compound **15** induced dose dependent apoptosis at all concentrations assayed with 95% (10 µM) in the DG-75 cell line, Figure 5C. Overall compounds **15**, **16b** and **16c** were shown to induce a superior potent pro-apoptotic response in both the MUTU-I and DG-75 BL cell lines than other selected compounds in this grouping and merit further study.

### 3.4. Evaluation of In Vitro Cytotoxicity of Ethanoanthracenes

**Figure 6:**
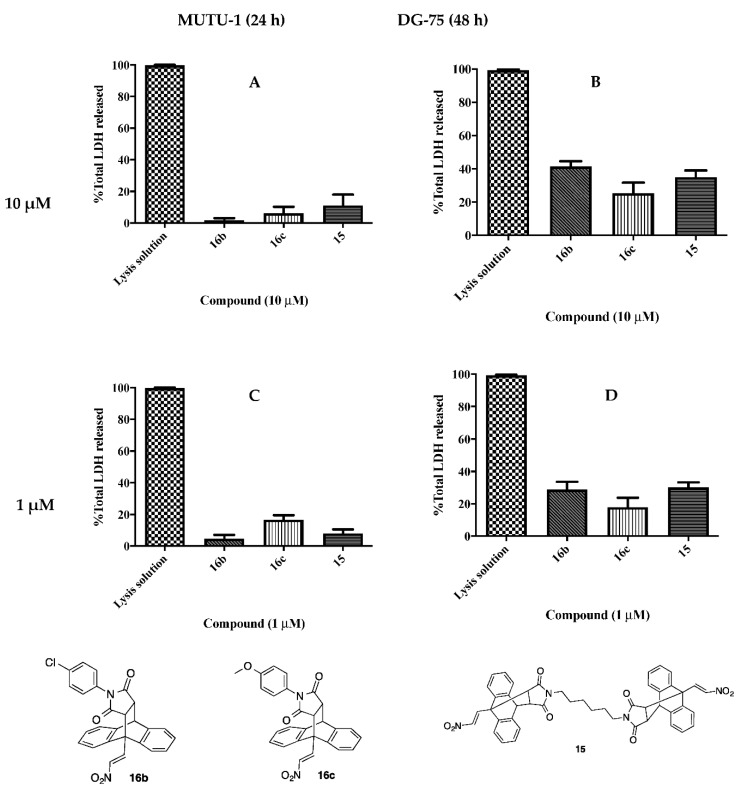
Lactate dehydrogenase (LDH) assay of ethanoanthracenes **15**, **16b** and **16c**. Cells were treated with ethanoanthracene compounds **15**, **16b** and **16c** (1 μM and 10 μM) for 24 or 48 h. Induction of necrotic cell death was determined by measuring LDH release in MUTU-I (**A**,**C**) and DG-75 cell lines (**B**,**D**), with control lysis solution (100% necrosis). Values are shown the mean of three independent experiments.

Compounds **15**, **16b** and **16c** were found to elicit the most potent anti-proliferative and pro-apoptotic activity of the series. In vitro cytotoxicity of these compounds was evaluated using a lactate dehydrogenase (LDH) assay. LDH is released during mechanisms of cell death associated with loss of cell membrane integrity (necrosis). The MUTU-I and DG-75 BL cell lines were treated at 10 µM and 1 µM for the desired treatment period and the results are presented as percentage of total LDH release, (Figure 6). In the MUTU-I cell line low levels of LDH release were obtained (2–17%) at 10 µM and 1 µM concentration, indicating low cytotoxicity. The lowest LDH release was observed by compound **16b** with 2% and 5% cytotoxicity (at 10 µM and 1 µM concentrations respectively). The compounds showed low to moderate cytotoxicity in the DG-75 cell line at 10 µM (25–41%) and 1 µM (27–30%). The lowest LDH release was observed for compound **16c** with 25% (10 µM) and 17% (1 µM).

### 3.5. Effect of Compounds ***15***, ***16b*** and ***16c*** on the Viability of PBMCs

**Figure 7:**
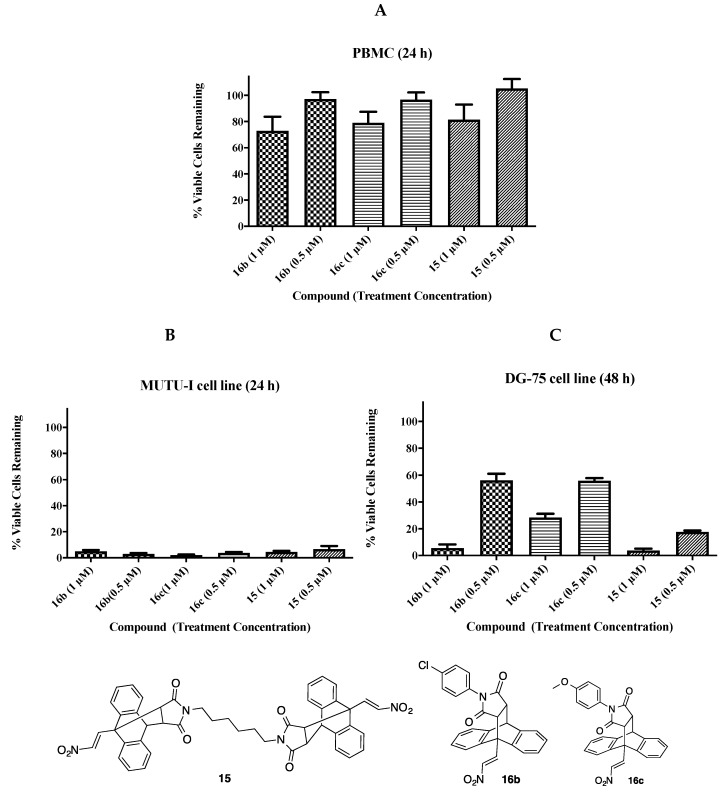
In Vitro antiproliferative effect of compounds **15**, **16b** and **16c** on (**A**) PBMCs (24 h), (**B**) MUTU-1 cell line (24 h) and (**C**) DG-75 cell line (48 h) at 1 μM and 0.5 μM.

The 2-nitrovinyl-9,10-dihydro-9,10-ethanoanthracenes compounds **15**, **16b** and **16c** were evaluated for their toxicity on *peripheral blood mononuclear cells* (PBMCs) to determine the selective toxicity of these compounds on malignant BL cell lines over normal lymphatic cells. Compounds were evaluated at 1 µM and 0.5 µM over a 24 h treatment time. Compounds **15**, **16b** and **16c** elicited minimal toxicity in the PBMCs at 0.5 µM (~99% viable cells) and low toxicity at 1 µM (72–81% viable cells), Figure 7. In comparison, compounds **15**, **16b** and **16c** induced a potent anti-proliferative effect in the MUTU-I at 1 µM and 0.5 µM, (2–7% viable cells remaining). Compounds **5**, **16b** and **16c** also induced a potent anti-proliferative effect in the DG-75 at 1 µM (6–28%) and 0.5 µM (18–57%). Comparing the results obtained from the BL cell lines and the PBMCs, the results suggest compounds **15**, **16b** and **16c** exert a selectively toxic effect on BL cell lines.

### 3.6. Effect of Pre-Treatment with Antioxidants on Cellular Viability

**Figure 8:**
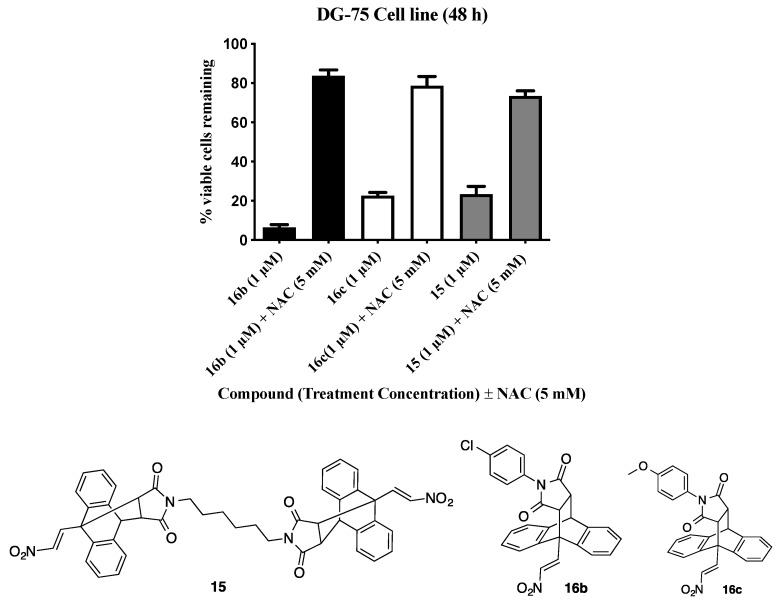
Effect of antioxidant pre-treatment on viability of DG-75 cells treated with compounds **15**, **16b** and **16c**.

Reactive oxygen species (ROS) are short lived diffusible entities containing oxygen such as hydroxy, nitroxyl, alkoxy, superoxide or peroxyl radicals. ROS are generated as metabolites of oxygen and are utilised for signalling events for essential cell functions. ROS are often associated with the induction of cell death and apoptosis. A potential role for ROS in the mechanism of cell death induced by selected potent compounds **15**, **16b** and **16c** was investigated. A viability assay was used to investigate the effects of pre-incubation with an antioxidant on ROS levels in DG-75 BL cells with the compound of interest. DG-75 cells were pre-treated with N-acetylcysteine (NAC), a known ROS scavenger and subsequently treated with the selected compounds **15**, **16b** and **16**. Viability was monitored using the alamarBlue assay, (Figure 8). From the results, obtained it is evident that in the presence of the reactive oxygen species inhibitor NAC—the anti-proliferative effects of compounds **15**, **16b** and **16** at 1 µM was reduced. Overall the anti-proliferative effects previously observed by compounds **16b**, **15** and **16c** increased from 6–23% viable cells to 73–83% viable cells in the presence of 5 mM NAC, indicating that ROS may be involved in the mechanism of cell death induced by these compounds, (Figure 8).

Cell proliferation of MUTU-1 and DG-75 cells was determined with an alamarBlue assay (seeding density 1–5 × 10^4^ cells/mL per well for 96-well plates). Compound concentrations of either 1 μM or 0.5 μM for 24 h (MUTU-1) or 48 h (DG-75) were used to treat the cells (in triplicate) with control wells containing vehicle ethanol (1% *v*/*v*). The mean value for three independent experiments is shown.

Cell proliferation of DG-75 cells was determined with an alamarBlue assay (seeding density 2 × 10^4^ cells/mL per well for 96-well plates), with vehicle control ethanol 1% (*v*/*v*). Cells were retained for 24 h and then pre-treated with NAC (5 mM) for 1 h, followed by compounds **15**, **16b** and **16c** at 1 μM for 48 h. Cell viability was measured by alamarBlue assay (mean of three independent experiments).

## 4. Conclusions

**Figure 9:**
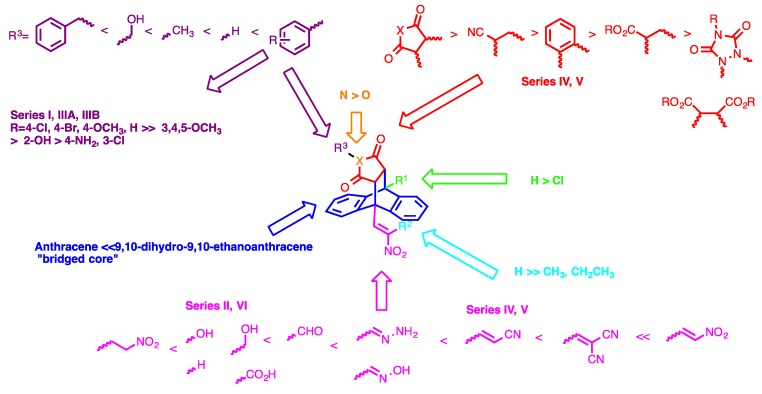
Summary of SAR for 9,10-dihydro-9,10-ethanoanthracenes.

A series of 9,10-dihydro-9,10-ethanoanthracene based maprotiline analogues were synthesised and evaluated for potential antiproliferative activity in the MUTU-I and chemoresistant DG-75 BL cell lines. Substitution at C-9 and C-10 of the 9,10-dihydro-9,10-ethanoanthracene compounds was achieved by modification of the diene system to include functionalities such as nitrovinyl, nitroalkyl, aldehyde, imine, carboxylic acid, alcohol, oxime, cyanovinyl and hydrazone on the anthracene scaffold. The effect of a number of 9,10-dihydro-9,10-ethanoanthracene structural modifications on activity was also investigated; these modifications included ethano bridge modifications, phenyl substitutions, maleimide substitutions and extension of alkyl chain length at C-2 of the nitrovinyl unit. The most promising 9,10-dihydro-9,10-ethanoanthracene based maprotiline analogues were identified and all included a nitrovinyl substituent at C-9. The structure-activity relationships for the series of ethanoanthracenes synthesised in this study are summarised in Figure 9. The preliminary screen of the 9,10-dihydro-9,10-ethanoanthracenes identified the maleimide compounds **15**, **16b** and **16c** as the lead compounds from this study. The dimer compound **15** displayed potent anticancer activity, in both BL lines with IC_50_ values of 0.09 µM (MUTU-I) and 0.24 µM (DG-75), while both compounds **16b** and **16c** elicited significant anti-proliferative activity in the BL cell lines. The present work has demonstrated the selectively toxic effect of compounds **15** and **16c** towards the MUTU-I and DG-75 cell lines over PBMCs. These compounds were shown to induce a significant pro-apoptotic effect in MUTU-I cell and DG-75 BL cell lines and may target the stress response to ROS in DG-75 BL cell lines. The results suggest that this class of compounds merits further investigation as antiproliferative agents for BL. Further studies are in progress to investigate the role of these compounds in ROS mediated cell death and in the reversal of drug efflux in multidrug resistant cancer cells.

## 5. Experimental Section

### 5.1. Chemistry

All commercially available reagents were used without further purification. Solvents were dried prior to use; tetrahydrofuran (THF) by distillation from sodium/benzophenone under nitrogen, toluene was distilled from sodium, dichloromethane was distilled from calcium hydride. Melting points were recorded on a Gallenkamp SMP 11 melting point apparatus and are uncorrected. Infra-red (IR) spectra were obtained on a Perkin Elmer FT-IR Spectrum 100 spectrometer. ^1^H and ^13^C NMR spectra were obtained on a Bruker Avance DPX 400 spectrometer operating at 400.13 MHz, (^1^H) and 100.61 MHz (^13^C) at 20 °C in either CDCl_3_ or DMSO-*d*_6_ with appropriate solvent peaks as reference standards. Mass spectrometry (ESI-MS) was performed on a Micromass LCT instrument with mass measurement accuracies of <±5 ppm. Low resolution mass spectra (LRMS) were obtained on a Hewlett-Packard 5973 MSD GC-MS system. Preparative separations were performed using flash column chromatography on silica gel (Merck Kieselgel 60, particle size 0.040–0.063 mm). Chromatographic separations were also performed on Biotage SP4 instrument. All reactions and products were monitored on thin layer chromatography (TLC) using Merck silica gel 60 F254. HPLC was used to determine the purity of the compounds (2487 Dual Wavelength Absorbance detector (Waters), 1525 binary HPLC pump, In-Line Degasser AF and Waters 717plus Autosampler), together with a Varian Pursuit XRs C18 reverse phase 150 × 4.6 mm chromatography column. Samples were detected using a wavelength of 254 nm. Details for the preparation of compounds **11a**–**11n**, **11p**–**11r**, **20a**, **20f**, **20g**, **21a**–**j**, **22a**–**c**, **22e** is contained in the Supplementary Information.

### 5.2. General Procedure 1: Preparation of Phenyl and Benzyl Maleimides *(**11a**–**11s**)*

To a solution of maleic anhydride (20 mmol) in diethyl ether (25 mL) was added the appropriate benzyl or aryl amine (20 mmol) in diethyl ether (10 mL). The reaction mixture was stirred under reflux at 20 °C for 1 h. The precipitated solid was filtered and washed with diethyl ether. This solid was immediately used in the next step and placed in a conical flask containing sodium acetate (8.5 mmol, 0.7 g) and acetic anhydride (10 mL). The mixture was heated at 90 °C for 0.5 h, then poured over ice water (100 mL). The solid was filtered and recrystallised from ethanol.

#### 5.2.1. 1-(3,4,5-Trimethoxyphenyl)-1*H*-pyrrole-2,5-dione (**11o**)

Compound **11o** was prepared from maleic anhydride (20 mmol) and 3,4,5-trimethoxyaniline (20 mmol) following the general procedure 1. The precipitated solid filtered and recrystallised from ethanol, to afford the product as yellow crystals, 3.17 g (60%), Mp. 120–128 °C. IR*_Vmax_* (KBr): 3085, 2964 (C-H), 1707 (C=O), 1509, 1470 (C=C), 1598 (C=C), 1127 (CN) cm^−1^. ^1^H NMR (400 MHz, CDCl_3_) δ 3.86 (s, 9 H, 3 x OCH_3_), 6.54 (s, 2 H, 2 x ArH), 6.84 (s, 2 H, 2 x =CH). ^13^C NMR (101 MHz, CDCl_3_) ppm 56.2 (OCH_3_), 60.8 (OCH_3_), 104.0 (CH), 126.6, 134.1 (CH), 137.7, 153.4, 169.5 (C=O). HRMS (APCI) calculated for C_13_H_14_NO_5_ [M^+^ + H] 264.0872: found 264.0867.

#### 5.2.2. 1,1′-(Hexane-1,6-diyl)bis(1*H*-pyrrole-2,5-dione) (**11s**)

Compound **11s** was prepared from maleic anhydride (20 mmol) and 1,4-diaminobenzene (20 mmol) following the general procedure 1. The product was obtained as yellow crystals (60%), Mp. 130–136 °C (lit. M.p. 136–141 °C [67]). IR*_Vmax_* (ATR): 3103, 2939 (C-H), 1688 (C=O), 1587, 1453 (C=C), 1227 (CN) cm^−1^. ^1^H NMR (400 MHz, CDCl_3_) δ 1.09–1.34 (m, 4 H, 2 x CH_2_), 1.34–1.56 (m, 4 H, 2 x CH_2_), 3.39 (t, *J =* 7.26 Hz, 4 H, 2 x CH_2_), 6.60 (s, 3 H, 2 x =CH). ^13^C NMR (101 MHz, CDCl_3_) ppm 25.9 (CH_2_), 28.1 (CH_2_), 37.4 (CH_2_), 133.9 (=CH), 169.7 (C=O), 170.7 (C=O). HRMS (APCI) calculated for C_14_H_17_N_2_O_4_ [M^+^ + H] 277.1188: found 277.1183.

### 5.3. General Procedure 2: Preparation of Nitrovinyl Anthracenes ***12a**–**12f***

To a solution of 9-anthraldehyde (2 g, 9.7 mmol) in the appropriate nitroalkane (nitromethane, nitroethane, nitropropane) (15 mL) was added piperidinium acetate (1.5 g, 10.3 mmol). (Piperidinium acetate was prepared from piperidine 6.6 mL and acetic acid 3 mL). The solution was heated at 90 °C for 1.5 h under nitrogen for 1 h, then cooled to room temperature and poured onto 100 mL of ice cold H_2_O. Following DCM extraction, the organic layers were combined, dried (Na_2_SO_4_) and solvent removed. The product was recrystallised from an appropriate solvent.

#### 5.3.1. (*E*)-9-(2-nitrovinyl)anthracene (**12a**)

Compound **12a** was prepared from 9-anthraldehyde (9.7 mmol, 2 g) and nitromethane (15 mL) as outlined in the general procedure 2. The product was recrystallized from methanol and diethyl ether as red crystals 2.41 g (99%), Mp. 145–147 °C (lit. M.p. 142 °C [68]). IR*_Vmax_* (KBr): 3050, 2948 (C-H), 1617, 1553 (C=C), 1498, 1330 (NO_2_), 1250 (C-N) cm^−1^. ^1^H NMR (400 MHz, CDCl_3_) δ 7.40–7.66 (m, 5 H, 4 x ArH, CH= x 1), 7.80–8.05 (m, 2 H, 2 x ArH), 8.05–8.25 (m, 2 H, 2 x ArH), 8.45 (br. s., 1 H, H10), 8.90 (d, *J* = 13.43 Hz, 1 H, CH= x 1). ^13^C NMR (101 MHz, CDCl_3_) ppm 124.3 (C9), 125.7 (CH), 127.5 (CH), 129.2 (CH), 129.2, 129.8, 130.4 (CH), 131.1, 135.6 (CH), 142.6 (C2′). HRMS (APCI) calculated for C_16_H_12_NO_2_ [M^+^ + H] 250.0868: found 250.0879.

#### 5.3.2. (*E*)-9-(2-Nitroprop-1-en-1-yl)anthracene (**12b**)

Compound **12b** was prepared from 9-anthraldehyde (9.7 mmol, 2 g) and nitroethane (15 mL) following the method in the general procedure 2. The product was recrystallized from ethanol and diethyl ether as orange crystals, 1.87 g (73%), Mp. 141–142 °C (lit. Mp. 142–143 °C [69]). IR*_Vmax_* (KBr): 3052, 2825 (C-H), 1510, 1326 (NO_2_), 1622.16, 1442.74 (C=C), 1483 (C=C), 385 (CH_3_) cm^−1^. ^1^H NMR (400 MHz, CDCl_3_) δ 1.98 (s, 1 H, CH_3_), 7.42–7.63 (m, 4 H, 4 x ArH), 7.86 (d, *J =* 7.93 Hz, 2 H, 2 x ArH), 7.93–8.12 (m, 2 H, 2 x ArH), 8.48 (s, 1 H, H10), 8.71 (s, 1 H, H1′). ^13^C NMR (101 MHz, CDCl_3_) ppm 14.4 (C3′), 124.8 (C9), 125.5 (CH), 126.7, 128.6, 128.8 (CH), 129.0 (CH), 131.0, 131.3 (CH), 151.0 (C2′). HRMS (ESI) calculated for C_17_H_14_NO_2_ [M^+^ + H] 264.1025: found 264.1035.

#### 5.3.3. (*E*)-9-(2-Nitrobut-1-en-1-yl)anthracene (**12c**)

Compound **12c** was prepared from 9-anthraldehyde (9.7 mmol, 2 g) and nitropropane (15 mL) as described in the general procedure 2. The product was recrystallized from ethanol and diethyl ether as gold crystals, 1.62 g (60%), Mp. 159–160 °C [70]. IR*_Vmax_* (KBr): 2982, 2937 C-H), 1622, 1427 (C=C), 1520, 1341 (NO_2_), 1444 (CH_2_), 1376 (CH_3_) cm^−1^. ^1^H NMR (400 MHz, CDCl_3_) δ 0.90 (t, *J* = 7.32 Hz, 3 H, CH_3_), 2.37 (q, *J* = 7.32 Hz, 2 H, CH2), 7.48–7.54 (m, 4 H,4 x ArH), 7.88–7.93 (m, 2 H, H4, H5), 8.01–8.05 (m, 2 H, H8, H1), 8.48 (s, 1 H, H10), 8.54 (s, 1 H, H1′). ^13^C NMR (101 MHz, CDCl_3_) ppm 11.6 (C3′), 21.4 (C2′), 124.9 (C9), 125.6 (CH), 125.7, 126.6 (CH), 128.3 (CH), 128.8, 128.9 (CH), 130.7 (CH), 131.1, 156.7 (C2′). HRMS (APCI) calculated for C_18_H_16_NO_2_ [M^+^ + H] 278.1181: found 278.1189.

#### 5.3.4. (*E*)-9-Chloro-10-(2-nitrovinyl)anthracene (**12d**)

Compound **12d** was prepared from 10-chloroanthracene-9-carbaldehyde (5 mmol, 1.2 g) and nitromethane (15 mL) as outlined in the general procedure 2. The product was recrystallized from methanol and diethyl ether as orange crystals, 1.01 g (71%), Mp. 232–234 °C. IR*_Vmax_* (ATR): 3066, 2973 (C-H), 1623 (C=C), 1439 (C=C), 1538, 1326 (NO_2_), 1110 (C-N) cm^−1^. ^1^H NMR (400 MHz, CDCl_3_) δ 7.52 (d, *J* = 14.04 Hz, 1 H, =CH), 7.60–7.77 (m, 4 H, 4 x ArH), 8.20 (d, *J* = 8.55 Hz, 2 H, 2 x ArH), 8.62 (d, *J* = 8.55 Hz, 2 H, 2 x ArH), 8.96 (d, *J* = 14.04 Hz, 1 H, =CH). ^13^C NMR (101 MHz, CDCl_3_) ppm 124.8, 125.8, 127.1, 127.6, 128.5, 130.1, 135.4 (C1), 143.3 (C2). HRMS (APCI) calculated for C_16_H_11_ClNO_2_ [M + H] 284.0478: found 284.0492.

#### 5.3.5. (*E*)-9-Chloro-10-(2-nitroprop-1-en-1-yl)anthracene (**12e**)

Compound **12e** was prepared from 10-chloroanthracene-9-carbaldehyde (5 mmol, 1.2 g) and nitroethane (15 mL) following general procedure 2. The product was recrystallized from methanol and diethyl ether as orange crystals 722 mg (50%), Mp. 158–160 °C. IR*_Vmax_* (ATR): 3023, 2977 (C-H), 1622 (C=C), 1480, 1438 (C=C), 1512, 1327 (NO_2_), 1171 (C-N) cm^−1^. ^1^H NMR (400 MHz, CDCl_3_) δ 1.99 (s, 3 H, CH_3_), 7.53–7.77 (m, 4 H, 4 x ArH), 7.92 (d, *J* = 8.55 Hz, 2 H, 2 x ArH), 8.60 (d, *J* = 9.16 Hz, 2 H, 2 x ArH), 8.70 (s, 1 H, H1′). ^13^C NMR (101 MHz*,* CDCl_3_) ppm 14.4 (C3′), 125.2, 125.6, 127.0, 127.0, 128.4, 129.2, 130.8, 131.0 (C1′), 151.5 (C2′). HRMS (APCI) calculated for C_17_H_13_ClNO_2_ [M^+^ + H] 289.0635: found 289.0637.

#### 5.3.6. (*E*)-9-Chloro-10-(2-nitrobut-1-en-1-yl)anthracene (**12f**)

Compound **12f** was prepared from 10-chloroanthracene-9-carbaldehyde (5 mmol, 1.2 g) and nitropropane (15 mL) as described in general procedure 2. The product was recrystallized from methanol and diethyl ether as orange crystals 998 mg (64%), Mp. 157–159 °C. IR*_Vmax_* (ATR): 3086, 2850 (C-H), 1621 (C=C), 1438, 1425 (C=C), 1553, 1328 (NO_2_), 1149 (C-N) cm^−1^. ^1^H NMR (400 MHz, CDCl_3_) δ 0.90 (t, *J* = 7.32 Hz, 3 H, CH_3_), 2.35 (q, *J* = 7.32 Hz, 2 H, CH_2_), 7.52–7.70 (m, 4 H, 4 x ArH), 7.94 (d, *J* = 8.55 Hz, 2 H, H4 & H6), 8.49 (s, 1 H, H1′), 8.57 (d, *J* = 8.55 Hz, 2 H, H1, H8). ^13^C NMR (101 MHz, CDCl_3_) ppm 11.5 (C4′), 21.4 (C3′) 125.3 (CH), 125.2 (CH), 125.4 (CH), 125.5, 126.9 (CH), 128.4, 129.1, 130.1 (C1′), 130.6, 157.0 (C2′). HRMS (APCI) calculated for C_18_H_15_ClNO_2_ [M^+^ + H] 312.0791: found 312.0797.

#### 5.3.7. 9-(2-Nitroethyl)anthracene (**12g**)

To a stirred solution of (*E*)-9-(2-nitrovinyl)anthracene (**12a**) (100 mg, 0.4 mmol) in dichloromethane (10 mL) and isopropanol (2 mL) was added sodium borohydride (60 mg, 1.6 mmol). After 24 h stirring at room temperature, the reaction mixture neutralised using 1 M HCl. The solution was extracted with CH_2_Cl_2_, dried with sodium sulphate and solvent removed *in vacuo*. The product was recrystallized from methanol and diethyl ether as orange crystals, 85 mg (85%), Mp. 147–149 °C. IR*_Vmax_* (ATR): 3053, 2974 (C-H), 1622, 1493 (C=C), 1546, 1377 (NO_2_), 1137 (C-N) cm^−1^. ^1^H NMR (400 MHz, CDCl_3_) δ 4.35–4.44 (m, 2 H, CH_2_), 4.70–4.78 (m, 2 H, CH_2_), 7.48–7.56 (m, 2 H, 2 x ArH), 7.57–7.65 (m, 2 H, 2 x ArH), 8.06 (d, *J* = 8.55 Hz, 2 H, 2 x ArH), 8.25 (d, *J* = 9.16 Hz, 2 H, 2 x ArH), 8.46 (s, 1 H, C10). ^13^C NMR (101 MHz, CDCl_3_) ppm 26.1 (C2′), 74.7 (C1′), 122.9 (CH), 125.2 (CH), 126.2, 126.9 (CH), 127.8, 129.6 (CH), 129.9, 131.5. HRMS (APCI) calculated for C_16_H_12_NO_2_ [M^+^ − H] 250.0874: found 250.0874.

### 5.4. General Procedure 3: Synthesis of Ethanoanthracenes

To a solution of the appropriate anthracene derivative (1 mmol) in toluene (2 mL) was added the required dienophile e.g., maleic anhydride, appropriate maleimide (1.3 mmol), 1-cyanovinyl acetate, methyl acrylate, ethyl acrylate and cyanoacrylate. The mixture was stirred and heated at 90 °C for 48 h, then cooled to RT and the product was obtained by filtration. The product was sequentially washed with toluene (2 mL) and diethyl ether (2 mL) and recrystallized from toluene.

#### 5.4.1. (*E*)-9-(2-Nitrovinyl)-9,10-dihydro-9,10-[3,4]furanoanthracene-12,14-dione (**13a**)

Compound **13a** was prepared from (*E*)-9-(2-nitrovinyl)anthracene **12a** (0.25 g, 1 mmol) and maleic anhydride (0.13 g, 1.3 mmol) following general procedure 3. The product was obtained as a colourless solid, 276 mg (80%), Mp. 244–245 °C. IR*_Vmax_* (KBr): 3030, 2966 (C-H), 1860, 1778 (C=O), 1662 (C=C), 1484, 1467 (C=C), 1525, 1353 (NO_2_) cm^−1^. ^1^H NMR (400 MHz, DMSO-*d*_6_) δ 3.81 (dd, *J* = 8.85, 3.36 Hz, 1 H, H11), 4.20 (d, *J* = 9.16 Hz, 1 H, H15), 4.98 (d, *J* = 3.66 Hz, 1 H, H10), 7.19–7.32 (m, 5 H, 5 x ArH), 7.32–7.40 (m, 1 H, ArH), 7.40–7.48 (m, 1 H,ArH), 7.57 (d, *J* = 6.71 Hz, 1 H, 1 x ArH), 8.10 (d, *J* = 14.04 Hz, 1 H, H1′), 8.28 (d, *J* = 13.43 Hz, 1 H, H2′). ^13^C NMR (101 MHz, DMSO-*d*_6_) ppm 44.2 (C10), 48.8 (C11), 49.2 (C9), 49.2 (C15), 123.1 (CH), 123.6 (CH), 124.6 (CH), 125.4 (CH), 126.7 (CH), 127.3 (CH), 127.4 (CH), 127.7 (CH), 136.3 (C2′), 138.2 (Cq), 138.4 (Cq), 140.5 (Cq), 140.8 (Cq), 145.3 (C1′), 170.1 (Cq, C=O, C12), 170.7 (Cq, C=O, C14). HRMS (APCI) calculated for C_20_H_12_NO_5_ [M^+^ − H] 346.0721: found 346.0721.

#### 5.4.2. (*E*)-9-(2-Nitroprop-1-en-1-yl)-9,10-dihydro-9,10-[3,4]furanoanthracene-12,14-dione (**13b**)

Compound **13b** was prepared from (*E*)-9-(2-nitroprop-1-en-1-yl)anthracene **12b** (0.26 g, 1 mmol) and maleic anhydride (0.13 g, 1.3 mmol) following general procedure 3. The product was obtained as a colourless solid 259 mg (72%), Mp. 256–258 °C. IR*_Vmax_* (KBr): 3009, 2960 (C-H), 1831, 1771 (C=O), 1641, 1521 (C=C), 1529, 1390 (NO_2_), 1333 (CH_3_) cm^−1^. ^1^H NMR (400 MHz, DMSO-*d*_6_) δ 1.81 (br. s., 3 H, -CH_3_), 3.65 (dd, *J* = 8.85, 2.75 Hz, 1 H, H11), 3.88 (d, *J* = 8.54 Hz, 1 H, H15), 4.95 (d, *J* = 2.44 Hz, 1 H, H10), 7.11–7.34 (m, 6 H, 6 x ArH), 7.41 (d, *J* = 6.71 Hz, 1 H, ArH), 7.56 (d, *J* = 7.32 Hz, 1 H, ArH), 8.16 (s, 1 H. H1′). ^13^C NMR (101 MHz, DMSO-*d*_6_) ppm 17.2 (C3′), 44.4 (C10), 48.5 (C11), 49.8 (C9), 51.8 (C15), 123.8 (CH), 124.0 (CH), 124.9 (CH), 125.4 (CH), 126.6 (CH), 127.2 (CH), 127.3 (CH), 127.8 (CH), 138.0 (C1′), 138.2 (Cq), 140.3 (Cq), 153.0 (C2′), 170.6 (C12), 170.7 (C14). HRMS (APCI) calculated for C_21_H_14_NO_5_ [M^+^ − H] 360.877: found 360.0865.

#### 5.4.3. (*E*)-9-(2-Nitrobut-1-en-1-yl)-9,10-dihydro-9,10-[3,4]furanoanthracene-12,14-dione (**13c**)

Compound **13c** was prepared from (*E*)-9-(2-nitrobut-1-en-1-yl)anthracene **12c** (0.27 g, 1 mmol) and maleic anhydride (0.13 g, 1.3 mmol) following general procedure 3. The product was obtained as a colourless solid, 336 mg (98%), Mp. 230–233 °C. IR*_Vmax_* (KBr): 2981, 2943 (C-H), 1837, 1775 (C=O), 1528, 1340 (NO_2_), 1479 (C=C), 1458 (CH_2_), 1386 (CH_3_) cm^−1^. ^1^H NMR (400 MHz, DMSO-*d*_6_) δ 0.92–1.04 (m, 3 H, -CH3) 2.19 (br. s., 2 H, CH2) 3.69 (d, *J* = 6.71 Hz, 1 H, H11) 3.93 (d, *J* = 8.55 Hz, 1 H, H15) 5.01 (d, *J* = 3.66 Hz, 1 H, H10) 7.19–7.35 (m, 6 H, 6 x ArH) 7.39–7.50 (m, 1 H, ArH) 7.52–7.66 (m, 1 H, ArH) 8.08 (s, 1 H, C1′). ^13^C NMR (101 MHz, DMSO-*d*_6_) ppm 9.5 (C4′), 23.4 (C3′), 44.6 (C10), 48.5 (C11), 49.9 (C9), 51.2 (C15), 123.3 (CH), 124.6 (CH), 125.4 (CH), 126.1 (CH), 126.5 (CH), 127.2 (CH), 127.3 (CH), 135.2 (C1′), 139.3 (Cq), 156.9 (C2′), 177.1 (C12), 177.3 (C14). HRMS (APCI) calculated for C_22_H_16_NO_5_ [M^+^ − H] 374.1034: found 374.1044.

#### 5.4.4. (*E*)-9-Chloro-10-(2-nitrovinyl)-9,10-dihydro-9,10-[3,4]furanoanthracene-12,14-dione (**13d**)

Compound **13d** was prepared from (*E*)-9-chloro-10-(2-nitrovinyl)anthracene **12d** (0.28 g, 1 mmol) and maleic anhydride (0.13 g, 1.3 mmol) following general procedure 3. The product was obtained as a colourless solid 58 mg (15%), Mp. 275–277 °C. IR*_Vmax_* (ATR): 3049, 2957 (C-H), 1706 (C=O), 1599 (C=C), 1456, 1420 (C=C), 1530, 1350 (NO_2_) cm^−1^. ^1^H NMR (400 MHz, DMSO-*d*_6_) δ 3.96 (d, *J* = 9.16 Hz, 1 H, H11), 4.44 (d, *J* = 9.16 Hz, 1 H, H15), 7.33–7.51 (m, 6 H, 6 x ArH), 7.71 (d, *J* = 7.32 Hz, 1 H, 1 x ArH), 7.83 (d, *J* = 7.94 Hz, 1 H, 1 x ArH), 8.12 (d, *J* = 14.04 Hz, 1 H, H1′), 8.28 (d, *J* = 14.04 Hz, 1 H, H2′). ^13^C NMR (101 MHz, DMSO-*d*_6_) ppm 48.3 (C10), 50.8 (C11), 55.0 (C15), 69.4 (C9), 122.5 (CH), 123.0 (CH), 123.4 (CH), 123.7 (CH), 127.8 (CH), 128.0 (CH), 128.3 (CH), 128.7 (CH), 135.1 (C2′), 136.7, 136.9, 139.4, 145.8 (C1′), 166.7 (C12), 168.8 (C14). HRMS (APCI) calculated for C_20_H_13_ClNO_5_ [M^+^ + H] 382.0482: found 382.0478.

#### 5.4.5. (*E*)-9-Chloro-10-(2-nitroprop-1-en-1-yl)-9,10-dihydro-9,10-[3,4]furanoanthracene-12,14-dione (**13e**)

Compound **13e** was prepared from (*E*)-9-chloro-10-(2-nitroprop-1-en-1-yl)anthracene (0.298 g, 1 mmol) **12e** and maleic anhydride (0.13 g, 1.3 mmol) following general procedure 3. The product was obtained as a colourless solid, 72 mg (18%), Mp. 270–272 °C. IR*_Vmax_* (ATR): 3012, 2977 (C-H), 1781 (C=O), 1656 (C=C), 1474, 1459 (C=C), 1516, 1334 (NO_2_) cm^−1^. ^1^H NMR (400 MHz, DMSO-*d*_6_) δ 1.82 (br. s., 3 H, CH_3_), 3.81 (d, *J* = 9.16 Hz, 1 H, H15), 4.18 (d, *J* = 8.55 Hz, 1 H, H11), 7.33–7.49 (m, 6 H, 6 x ArH), 7.74 (d, *J* = 7.32 Hz, 1 H, 1 x ArH), 7.86 (d, *J* = 7.94 Hz, 1 H, 1 x ArH), 8.20 (s, 1 H, H1′). ^13^C NMR (101 MHz, DMSO-*d*_6_) ppm 17.4 (C3′), 49.0 (C10), 53.1 (C11), 54.9 (C15), 69.7 (C9), 122.9 (CH), 123.1 (CH), 124.0 (CH), 127.8 (CH), 127.9 (CH), 128.3 (CH), 128.5 (CH), 136.7, 136.9, 139.0, 153.4 (C2′), 166.8 (C12, C14). HRMS (APCI) calculated for C_21_H_14_NClO_5_ [M^+^] 395.0561: found 395.0553.

#### 5.4.6. (*E*)-9-Chloro-10-(2-nitrobut-1-en-1-yl)-9,10-dihydro-9,10-[3,4]furanoanthracene-12,14-dione (**13f**)

Compound **13f** was prepared from (*E*)-9-chloro-10-(2-nitrobut-1-en-1-yl)anthracene **12f** (0.312 g, 1 mmol) and maleic anhydride (0.13 g, 1.3 mmol) following general procedure 3. The product was obtained as a colourless solid 224 mg (55%), Mp. 259–263 °C. IR*_Vmax_* (KBr): 3065, 2951 (C-H), 1711 (C=O), 1610 (C=C), 1454 (C=C), 1530, 1346 (NO_2_) cm^−1^. ^1^H NMR (400 MHz, DMSO-*d*_6_) δ 0.99 (br. s., 3 H, CH_3_) 2.02–2.23 (m, 2 H, CH_2_) 3.80 (br. s., 1 H, H15) 4.18 (d, *J* = 8.55 Hz, 1 H, H11) 7.29–7.50 (m, 6 H, 6 x ArH) 7.75 (d, *J* = 7.32 Hz, 1 H, 1 x ArH) 7.87 (d, *J* = 7.32 Hz, 1 H, 1 x ArH) 8.06 (br. s., 1 H, H1′). ^13^C NMR (101 MHz, DMSO-*d*_6_) ppm 55.0 (C10), 56.4 (C15, C11), 69.7 (C9), 115.6 (CH), 122.9 (CH), 123.1 (CH), 123.8 (CH), 127.9 (CH), 128.0 (CH), 128.5 (C1′), 137.1, 149.7 (C2′), 157.7 (C15, C11). HRMS (APCI) calculated for C_22_H_16_ClNO_5_ [M^+^] 409.0717: found 409.0717.

#### 5.4.7. (*E*)-9-(2-Nitrovinyl)-9,10-dihydro-9,10-[3,4]epipyrroloanthracene-12,14-dione (**13g**)

Compound **13g** was prepared from (*E*)-9-(2-nitrovinyl)anthracene **12a** (0.25 g, 1 mmol) and maleimide (0.13 g, 1.3 mmol) following general procedure 3. The product was obtained as a colourless solid 172 mg (50%), Mp. 176–178 °C. IR*_Vmax_* (KBr): 3551 (N-H), 3058, 2963 (Ar C-H), 1722 (C=O), 1527, 1354 (NO_2_), 1167 (N-C) cm^−1^. ^1^H NMR (400 MHz, DMSO-*d*_6_) δ 3.28 (br. s., 1 H, H11), 3.70 (d, *J* = 8.54 Hz, 1 H, H15), 4.79 (br. s., 1 H, H10), 6.99–7.36 (m, 7 H, 7 x ArH), 7.51 (d, *J* = 6.71 Hz, 1 H, ArH), 8.04 (d, *J* = 14.04 Hz, 1 H, H1′), 8.24 (d, *J* = 13.43 Hz, 1 H, H2′), 10.89 (br. s., 1 H, NH). ^13^C NMR (101 MHz, DMSO-*d*_6_) ppm 44.3 (C10), 48.6 (C11) 48.7 (C15), 49.2 (C9), 122.9 (CH), 123.3 (CH), 124.3 (CH), 125.3 (CH), 126.3 (CH), 126.8 (CH), 127.0 (CH), 127.2 (CH), 137.5 (C2′), 138.5 (Cq), 138.7 (Cq), 141.4 (Cq), 141.7 (Cq), 145.1 (C1′), 176.9 (C12), 177.1 (C14). HRMS (APCI) calculated for C_20_H_13_N_2_O_4_ [M^+^ − H] 345.0881: found 345.0896.

#### 5.4.8. (*E*)-9-(2-Nitroprop-1-en-1-yl)-9,10-dihydro-9,10-[3,4]epipyrroloanthracene-12,14-dione (**13h**)

Compound **13h** was prepared from (*E*)-9-(2-nitroprop-1-en-1-yl)anthracene **12b** (0.26 g, 1 mmol) and maleimide (0.13 g, 1.3 mmol) following general procedure 3. The product was obtained as a colourless solid, 348 mg (97%), Mp. 296–300 °C. IR*_Vmax_* (KBr): 3368 (N-H), 3090, 3008 (C-H), 1520, 1350 (NO_2_), 1771 (C=O), 1346 (CH_3_) cm^−1^. ^1^H NMR (400 MHz, DMSO-*d*_6_) δ 1.80 (br. s., 3 H, CH3), 3.17 (dd, *J* = 8.54, 3.05 Hz, 1 H, H11), 3.35 (d, *J* = 7.93 Hz, 1 H, H15), 4.79 (d, *J* = 3.66 Hz, 1 H, H10), 7.13–7.27 (m, 6 H, 6 x ArH), 7.33 (d, *J* = 6.71 Hz, 1 H, ArH), 7.52 (d, *J* = 7.32 Hz, 1 H, ArH), 8.28 (s, 1 H, H1′), 10.83 (s, 1 H, NH). ^13^C NMR (101 MHz, DMSO-*d*_6_) ppm 17.2 (C3′), 44.5 (C10), 48.3 (C11), 49.8 (C9), 51.1 (C15), 123.4 (CH), 124.6 (CH), 125.3 (CH), 126.2 (CH), 126.6 (CH), 127.0 (CH), 127.2 (CH), 131.9 (C1′), 138.4 (Cq), 139.0 (Cq), 141.2 (Cq), 152.4 (C2′), 177.1 (C12), 177.4 (C14). HRMS (APCI) calculated for C_21_H_16_N_2_O_4_ [M^+^ − H] 359.1037: found 359.1024.

#### 5.4.9. (*E*)-9-(2-Nitrobut-1-en-1-yl)-9,10-dihydro-9,10-[3,4]epipyrroloanthracene-12,14-dione (**13i**)

Compound **13i** was prepared from (*E*)-9-(2-nitrobut-1-en-1-yl)anthracene **12c** (0.27 g, 1 mmol) and maleimide (0.13 g, 1.3 mmol) according following general procedure 3. The product was isolated as a colourless solid, 283 mg (76%), Mp. 296–298 °C. IR*_Vmax_* (KBr): 3182 (N-H), 3015, 2988 (C-H), 1707 (C=O), 1523, 1348 (NO_2_), 1457 (CH_2_) cm^−1^. ^1^H NMR (400 MHz, DMSO-*d*_6_) δ 3.00 (br. s., 3 H, CH3), 2.12 (br. s., 2 H, CH2), 3.17 (br. s., 1 H, H11), 3.35 (br. s., 1 H, H15), 4.81 (d, *J* = 2.44 Hz, 1 H, H10), 7.09–7.27 (m, 6 H, 6 x ArH), 7.28–7.40 (m, 1 H, ArH), 7.53 (d, *J* = 6.10 Hz, 1 H, ArH), 8.13 (s, 1 H, H1′), 10.84 (s, 1 H). ^13^C NMR (101 MHz, DMSO-*d*_6_) ppm 9.5 (C4′), 23.4 (C3′), 44.6 (C10), 48.5 (C11), 49.8 (C9), 51.2 (C15), 123.3 (CH), 124.6 (CH), 125.4 (CH), 126.1 (CH), 126.5 (CH), 127.2 (CH), 127.3 (CH), 135.2, 139.2 (C1′), 156.9 (C2′), 177.1 (C12), 177.3 (C14). HRMS (APCI) calculated for C_22_H_17_N_2_O_4_ [M^+^ − H] 373.1194: found 373.1176.

#### 5.4.10. (*E*)-9-Chloro-10-(2-nitrovinyl)-9,10-dihydro-9,10-[3,4]epipyrroloanthracene-12,14-dione (**13j**)

Compound **13j** was prepared from (*E*)-9-chloro-10-(2-nitrovinyl)anthracene **12d** (0.28 g, 1 mmol) and maleimide (0.13 g, 1.3 mmol) as outlined in general procedure 3. The product was isolated as a colourless solid, 95 mg (25%), Mp. >300 °C. IR*_Vmax_* (ATR): 3050, 2963 (C-H), 1707 (C=O), 1599 (C=C), 1457, 1419 (C=C), 1518, 1349 (NO_2_) cm^−1^. ^1^H NMR (400 MHz, DMSO-*d*_6_) δ 3.51 (d, *J* = 8.55 Hz, 1 H, H11), 3.95 (d, *J* = 8.55 Hz, 1 H, H15), 7.25–7.49 (m, 6 H, 6 x ArH), 7.67 (d, *J* = 7.32 Hz, 1 H, 1 x ArH), 7.80 (d, *J* = 7.32 Hz, 1 H, 1 x ArH), 8.08 (d, *J* = 14.04 Hz, 1 H, H1′), 8.27 (d, *J* = 14.04 Hz, 1 H, H2′), 11.09 (br. s., 1 H, NH). ^13^C NMR (101 MHz, DMSO-*d*_6_) ppm 48.3 (C10), 50.4 (C11), 54.1 (C15), 70.2 (C9), 122.3 (CH), 123.0 (CH), 123.1 (CH), 123.3 (CH), 127.4 (CH), 127.6 (CH), 127.7, 128.1 (CH), 136.3 (C2′), 137.1, 137.3, 140.1, 140.4, 145.6 (C1′), 173.4 (C12), 175.5 (C14). HRMS (APCI) calculated for C_20_H_12_ClN_2_O_4_ [M^+^ − H] 379.0491: found 379.0478.

#### 5.4.11. (*E*)-9-Chloro-10-(2-nitroprop-1-en-1-yl)-9,10-dihydro-9,10-[3,4]epipyrroloanthracene-12,14-dione (**13k**)

Compound **13k** was prepared from (E)-9-chloro-10-(2-nitroprop-1-en-1-yl)anthracene **12e** (0.298 g, 1 mmol) and maleimide (0.13 g, 1.3 mmol) as described in general procedure 3. The product was isolated as a colourless solid 197 mg (50%), Mp. 295–297 °C. IR*_Vmax_* (ATR): 3368 (N-H), 3067, 2942 (C-H), 1711 (C=O), 1610 (C=C), 1455 (C=C), 1521, 1344 (NO_2_) cm^−1^. ^1^H NMR (400 MHz, DMSO-*d*_6_) δ 1.81 (br. s., 3 H, CH_3_), 3.38 (d, *J* = 8.55 Hz, 1 H, H15), 3.60 (d, *J* = 7.94 Hz, 1 H, H11), 7.28–7.47 (m, 6 H, 6 x ArH), 7.70 (d, *J* = 7.94 Hz, 1 H, 1 x ArH), 7.84 (d, *J* = 7.94 Hz, 1 H, 1 x ArH), 8.32 (s, 1 H, H1′), 11.04 (s, 1 H, NH). ^13^C NMR (101 MHz, DMSO-*d*_6_) ppm 17.4 (C3′), 49.0 (C10), 52.6 (C11), 53.9 (C15), 70.4 (C9), 122.7 (CH), 123.0 (CH), 123.5 (CH), 124.1, 127.5 (CH), 127.6 (CH), 127.7 (CH), 127.9 (CH), 130.8, 135.1, 137.1, 137.6, 140.1, 152.8 (C2′), 173.5 (C12), 175.9 (C14). HRMS (APCI) calculated for C_21_H_16_ClN_2_O_4_ [M^+^ + H] 395.0799: found 395.0787.

#### 5.4.12. (*E*)-9-Chloro-10-(2-nitrobut-1-en-1-yl)-9,10-dihydro-9,10-[3,4]epipyrroloanthracene-12,14-dione (**13l**)

Compound **13l** was prepared from (*E*)-9-chloro-10-(2-nitrobut-1-en-1-yl)anthracene **12f** (0.312 g, 1 mmol) and maleimide (0.13 g, 1.3 mmol) as described in general procedure 3. The product was isolated as a colourless solid 224 mg (55%), Mp. 294–298 °C. IR*_Vmax_* (ATR): 3369 (N-H), 3022, 2942 (C-H), 1710 (C=O), 1608 (C=C), 1455, 1426 (C=C), 1519, 1342 (NO_2_) cm^−1^. ^1^H NMR (400 MHz, DMSO-*d*_6_) δ 0.98 (br. s., 3 H, CH_3_), 2.11 (br. s., 2 H, CH_2_), 3.36 (d, *J* = 7.32 Hz, 1 H, H15), 3.48–3.76 (m, 1 H, H11), 7.26 (m, 1 H, 1 x ArH), 7.30–7.51 (m, 5 H, 5 x ArH), 7.71 (d, *J* = 7.32 Hz, 1 H, 1 x ArH), 7.85 (d, *J* = 7.94 Hz, 1 H, 1 x ArH), 8.16 (s, 1 H, H1′), 11.04 (br. s., 1 H, NH). ^13^C NMR (101 MHz, DMSO-*d*_6_) ppm 9.4 (C4′), 23.5 (C3′), 49.2 (C10), 52.6 (C11), 54.0 (C15), 70.4 (C9), 122.7 (CH), 123.1 (CH), 123.4 (CH), 127.5 (CH), 127.6 (CH), 127.8 (CH), 130.5, 137.8, 157.2 (C2′), 173.5 (C12), 175.9 (C14). HRMS (APCI) calculated for C_22_H_18_ClN_2_O_4_ [M^+^ − H] 409.0955: found 409.0974.

#### 5.4.13. (*E*)-13-Methyl-9-(2-nitrovinyl)-9,10-dihydro-9,10-[3,4]epipyrroloanthracene-12,14-dione (**13m**)

(*E*)-9-(2-nitrovinyl)anthracene **12a** (0.25 g, 1 mmol) was reacted with 1-methyl-1*H*-pyrrole-2,5-dione **11r** (0.144 g, 1.3 mmol) as described in general procedure 3 and isolated as a yellow solid 198 mg (55%), Mp. 267–268 °C. IR*_Vmax_* (ATR): 3070, 2951 (C-H), 1713 (C=O), 1594 (C=C), 1481, 1450 (C=C), 1531, 1349 (NO_2_) cm^−1^. ^1^H NMR (400 MHz, DMSO-*d*_6_) δ 2.35 (s, 3 H, CH_3_), 3.38 (dd, *J* = 8.24, 2.75 Hz, 1 H, H11), 3.78 (d, *J* = 8.55 Hz, 1 H, H15), 4.87 (d, *J* = 2.44 Hz, 1 H, H10), 7.15–7.36 (m, 7 H, 7 x ArH), 7.56 (d, *J* = 6.71 Hz, 1 H, 1 x ArH), 8.11 (d, *J* = 13.43 Hz, 1 H, H1′), 8.29 (d, *J* = 13.43 Hz, 1 H, H2′). ^13^C NMR (101 MHz, DMSO-*d*_6_) ppm 24.0 (CH_3_), 44.4 (C10), 47.5 (C11), 47.6 (C15), 49.3 (C9), 122.8 (CH), 123.4 (CH), 124.4 (CH), 125.1 (CH), 126.4 (CH), 126.8 (CH), 127.1 (CH), 127.2 (CH), 137.4 (CH), 138.2, 138.4, 141.1, 141.5, 145.1 (C1′), 175.4 (C12), 175.8 (C14). HRMS (APCI) calculated for C_21_H_17_N_2_O_4_ [M^+^ + H] 361.1188: found 361.1180.

#### 5.4.14. (*E*)-13-(Hydroxymethyl)-9-(2-nitrovinyl)-9,10-dihydro-9,10-[3,4]epipyrroloanthracene-12,14-dione (**13n**)

Compound **13n** was prepared from (*E*)-9-(2-nitrovinyl)anthracene **12a** 0.25 g (1 mmol) and 1-(hydroxymethyl)-1*H*-pyrrole-2,5-dione **11q** 0.165 g (1.3 mmol) as described in general procedure 3 and isolated as a colourless solid, 301 mg (80%), Mp. 230–232 °C. IR*_Vmax_* (KBr): 3463 (OH), 2964, 2899 (C-H), 1778 (C=O), 1698 (C=C), 1523 (NO), 1482, 1456 (C=C), 1198 (C-N stretch) cm^−1^. ^1^H NMR (400 MHz, DMSO-*d*_6_) δ 3.43 (dd, *J* = 8.55, 3.05 Hz, 1 H, H11), 3.85 (d, *J* = 8.55 Hz, 1 H, H15), 4.19–4.31 (m, 2 H, CH_2_), 4.88 (d, *J* = 3.05 Hz, 1 H, H10), 6.12 (t, *J* = 7.02 Hz, 1 H, OH), 7.14–7.35 (m, 7 H, 7 x ArH), 7.57 (d, *J* = 7.32 Hz, 1 H, 1 x ArH), 8.13 (d, *J* = 14.04 Hz, 1 H, H1′), 8.30 (d, *J* = 14.04 Hz, 1 H, H2′). ^13^C NMR (101 MHz, DMSO-*d*_6_) ppm 44.4 (C10), 47.4 (C11), 47.4 (C15), 49.3 (C9), 60.2 (CH_2_), 122.8 (CH), 123.3 (CH), 124.4 (CH), 125.2 (CH), 126.4 (CH), 126.8 (CH), 127.1 (CH), 127.2 (CH), 137.4 (C2′), 138.2, 138.4, 141.3, 141.7, 145.1 (C1′), 174.9 (C12), 175.3 (C14). HRMS (APCI) calculated for C_21_H_17_N_2_O_5_ [M^+^ + H] 377.1137: found 377.1138.

#### 5.4.15. 9-(2-Nitroethyl)-9,10-dihydro-9,10-[3,4]furanoanthracene-12,14-dione (**14a**)

Compound **14a** was prepared from 9-(2-nitroethyl)anthracene **12a** (0.25 g, 1 mmol) and maleic anhydride (0.13 g, 1.3 mmol) according to general procedure 3 to give the product as a colourless solid 150 mg (43%), Mp. 242–244 °C. IR*_Vmax_* (ATR): 3065, 2974 (C-H), 1773 (C=O), 1581 (C=C), 1469, 1457 (C=C), 1546, 1338 (NO_2_) cm^−1^. ^1^H NMR (400 MHz, DMSO-*d*_6_) δ 3.33–3.52 (m, 2 H, CH_2_), 3.60 (d, *J* = 9.16 Hz, 1 H, H11), 3.78 (d, *J* = 9.16 Hz, 1 H, H15), 4.88 (br. s., 1 H, H10), 5.06 (t, *J* = 14.65 Hz, 1 H, H1′a), 5.24 (t, *J* = 10.38 Hz, 1 H, H1′b), 7.25 (quin, *J* = 7.02 Hz, 4 H, 4 x ArH), 7.33–7.43 (m, 2 H, 2 x ArH), 7.53 (d, *J* = 6.71 Hz, 1 H, 1 x ArH), 7.49 (d, *J* = 7.32 Hz, 1 H, 1 x ArH). ^13^C NMR (101 MHz, DMSO-*d*_6_) ppm 25.6 (C1′), 44.4 (C10), 46.3 (C11), 48.4 (C9), 49.0 (C15), 72.8 (C2′), 122.6 (CH), 122.9 (CH), 125.3 (CH), 125.5 (CH), 127.1 (CH), 127.1, 127.7 (CH), 127.7, 139.7, 140.7, 141.3, 142.4, 170.9 (C12), 171.5 (C14). HRMS (APCI) calculated for C_20_H_16_NO_5_ [M^+^ + H] 350.1028: found 350.1030.

#### 5.4.16. 9-(2-Nitroethyl)-9,10-dihydro-9,10-[3,4]epipyrroloanthracene-12,14-dione (**14b**)

Compound **14b** was prepared from 9-(2-nitroethyl)anthracene **11b** (0.25 g, 1 mmol) and maleimide (0.13 g, 1.3 mmol) according to general procedure 3 to give the product as a colourless solid, 157 mg (45%), Mp. 295–297 °C. IR*_Vmax_* (ATR): 3049, 2936 (C-H), 1702 (C=O), 1599 (C=C), 1456, 1419 (C=C), 1548, 1349 (NO_2_) cm^−1^. ^1^H NMR (400 MHz, DMSO-*d*_6_) δ 3.16 (d, *J* = 8.55 Hz, 1 H, H2′a), 3.23–3.31 (m, 2 H, H2′b, H11), 3.44–3.62 (m, 1 H, H15), 4.72 (br. s., 1 H, C10), 5.15 (d, *J* = 7.32 Hz, 2 H, CH_2_), 7.11–7.37 (m, 6 H, 6 x ArH), 7.48 (t, *J* = 8.55 Hz, 2 H, 2 x ArH), 10.89 (br. s., 1 H, NH). ^13^C NMR (101 MHz, DMSO-*d*_6_) ppm 25.5 (C1′), 44.1 (C10), 45.6 (C11), 46.9 (C15), 48.3 (C9), 72.7 (C2′), 121.9, 122.1 (CH), 124.5 (CH), 124.9 (CH), 126.3 (CH), 126.3 (CH), 126.6 (CH), 126.7 (CH), 139.6, 140.8, 142.0, 142.8, 177.3 (C12), 177.6 (C14). HRMS (APCI) calculated for C_20_H_17_N_2_O_5_ [M^+^ + H] 349.1188: found 349.1181.

#### 5.4.17. 9-(2-Nitroethyl)-13-phenyl-9,10-dihydro-9,10-[3,4]epipyrroloanthracene-12,14-dione (**14c**)

Compound **14c** was prepared from 9-(2-nitroethyl)anthracene **12b** (0.25 g, 1 mmol) and phenylmaleimide (0.224 g, 1.3 mmol) according to general procedure 3, to give the product as a colourless solid, 127 mg (30%), Mp. 248–250 °C. IR*_Vmax_* (ATR): 2964, 2939 (C-H), 1710 (C=O), 1595 (C=C), 1491, 1456 (C=C), 1541, 1388 (NO_2_) cm^−1^. ^1^H NMR (400 MHz, DMSO-*d*_6_) δ 3.37 (d, *J* = 8.55 Hz, 2 H, H2′a), 3.49–3.60 (m, 2 H, H2′b, H15), 4.87 (br. s., 1 H, C10), 5.10–5.31 (m, 2 H, CH_2_), 6.44 (d, *J* = 5.49 Hz, 2 H, 2 x ArH), 7.20–7.42 (m, 9 H, 9 x ArH), 7.57 (d, *J* = 7.32 Hz, 1 H, 1 x ArH), 7.54 (d, *J* = 7.32 Hz, 1 H, 1 x ArH). ^13^C NMR (101 MHz, DMSO-*d*_6_) ppm 25.5 (C1′), 44.6 (C10), 46.2 (C11), 46.2 (C9), 47.4 (C15), 72.7 (C2′), 122.0 (CH), 122.3 (CH), 124.7 (CH), 125.0 (CH), 126.5 (CH), 126.5 (CH), 126.7 (CH), 126.8 (CH), 128.5 (CH), 128.9 (CH), 131.6, 139.4, 140.5, 141.6, 142.4, 175.0 (C12), 175.5 (C14). HRMS (APCI) calculated for C_26_H_21_NO_4_ [M^+^ + H] 425.1501: found 425.1512.

#### 5.4.18. 13,13′-(Hexane-1,6-diyl)bis(9-((*E*)-2-nitrovinyl)-9,10-dihydro-9,10[3,4]epipyrroloanthracene-12,14-dione) (**15**)

Compound **15** was prepared from (*E*)-9-(2-nitrovinyl)anthracene **12a** (0.25 g, 1 mmol) and the required maleimide **11s** (0.5 mmol) following the general procedure 3 to afford the product as a colourless solid, 39 mg (10%), Mp. 186–188 °C. IR*_Vmax_* (KBr): 3050, 2961 (Ar C-H), 1699 (C=O), 1533, 1358 (NO_2_), 1203 (N-C) cm^−1^. ^1^H NMR (400 MHz, DMSO-*d*_6_) δ 0.43 (br. s., 4 H, 2 x CH_2_), 0.47–0.57 (m, 4 H, 2 x CH_2_), 2.86–2.94 (m, 4 H, 2 x NCH_2_), 3.36 (dd, *J* = 8.44, 3.30 Hz, 2 H, 2 x H11), 3.76 (dd, *J* = 8.44, 1.83 Hz, 2 H, 2 x H15), 4.86–4.92 (m, 2 H, 2 x H10), 7.18–7.31 (m, 12 H, 12 x ArH), 7.31–7.38 (m, 2 H, 2 x ArH), 7.54–7.59 (m, 2 H, 2 x ArH), 8.12 (d, *J* = 13.94 Hz, 2 H, 2 x H1′), 8.27–8.34 (m, 2 H, 2 x H2′). ^13^C NMR (101 MHz, DMSO-*d*_6_) ppm 25.14 (CH_2_), 26.17 (CH_2_), 37.67 (NCH_2_), 44.45 (C10, C10′), 47.24 (C11, C11′), 47.36 (C15, C15′), 49.35 (C9, C9′), 122.87 (CH), 123.32 (CH), 124.35 (CH), 125.19 (CH), 126.39 (CH), 126.79 (CH), 127.07 (CH), 137.33 (C2′), 138.44, 138.54, 141.29, 141.61, 145.15 (C1′), 175.35 (C11), 175.73 (C15). HRMS (APCI) calculated for C_46_H_39_N_4_O_8_ [M^+^ + H] 775.2768: found 775.2771.

#### 5.4.19. (E)-9-(2-Nitrovinyl)-13-phenyl-9,10-dihydro-9,10-[3,4]epipyrroloanthracene-12,14-dione (**16a**)

Compound **16a** was prepared from (*E*)-9-(2-nitrovinyl)anthracene **12a** (0.25 g, 1 mmol) and phenylmaleimide (0.23 g, 1.3 mmol) as described in general procedure 3, to give the product as a colourless solid 126 mg (30%), Mp. 256–257 °C. IR*_Vmax_* (KBr): 3035, 2961 (C-H), 1709 (C=O), 1529, 1349 (NO_2_), 1596, 1457 (C=C), 1201 (C-N) cm^−1^. ^1^H NMR (400 MHz, DMSO-*d*_6_) δ 3.51 (m, *J* = 8.24, 2.75 Hz, 1 H, H11), 3.91 (d, *J* = 8.54 Hz, 1 H, H15), 4.86–4.98 (m, 1 H, H10), 6.28–6.46 (m, 2 H, H6″, H2″), 7.14–7.34 (m, 10 H, 10 x ArH), 7.58 (d, *J* = 6.71 Hz, 1 H, ArH), 8.08 (d, *J* = 14.04 Hz, 1 H, H1′), 8.28 (d, *J* = 14.04 Hz, 1 H, H2′). ^13^C NMR (101 MHz, DMSO-*d*_6_) ppm 44.8 (C10), 47.6 (C11), 47.9 (C15), 49.6 (C9), 123.1 (CH), 123.5 (CH), 124.5 (CH), 125.3 (CH), 126.5 (CH), 126.97 (CH), 127.16 (CH), 127.31 (CH), 128.6 (CH), 128.8 (CH), 131.5 (CH), 137.3 (C2′), 138.5 (Cq), 140.9 (Cq), 141.3 (Cq), 145.2 (C1′), 174.7 (C12), 175.1 (C14). HRMS (APCI) calculated for C_26_H_19_N_2_O_4_ [M^+^ + H] 423.1345: found 423.1364.

#### 5.4.20. (*E*)-13-(4-Chlorophenyl)-9-(2-nitrovinyl)-9,10-dihydro-9,10-[3,4]epipyrroloanthracene-12,14-dione (**16b**)

Compound **16b** was prepared from (*E*)-9-(2-nitrovinyl)anthracene (0.25 g, 1 mmol) and 1-(4-chlorophenyl)-1*H*-pyrrole-2,5-dione **11c** (0.27 g, 1.3 mmol) following general procedure 3, and afforded the product as a colourless solid 233 mg (51%), Mp. 238–241 °C. IR*_Vmax_* (KBr): 3035, 2946 (C-H), 1714 (C=O), 1659 (C=C), 1537, 1493 (C=C), 1191 (C-N) cm^−1^. ^1^H NMR (400 MHz, DMSO-*d*_6_) δ 3.55 (dd, *J* = 8.54, 3.05 Hz, 1 H, H11), 3.95 (d, *J* = 7.93 Hz, 1 H, H15), 4.97 (d, *J* = 3.05 Hz, 1 H, H10), 6.48 (d, *J* = 8.54 Hz, 2 H, H6″, H2″), 7.21–7.47 (m, 10 H, 10 x ArH), 7.61 (d, *J* = 6.71 Hz, 1 H, ArH), 8.12 (d, *J* = 14.04 Hz, 1 H, H1′), 8.32 (d, *J* = 14.04 Hz, 1 H, H2′). ^13^C NMR (101 MHz, DMSO-*d*_6_) ppm 44.8 (C10), 47.7 (C11), 48.0 (C15), 49.6 (C9), 123.0 (CH), 123.5 (CH), 124.5 (Cq), 125.3 (CH), 126.5 (CH), 127.0 (CH), 127.2 (Cq), 127.3 (CH), 128.1 (CH), 129.0 (CH), 130.3 (Cq), 133.1 (Cq), 137.2 (C2′), 138.4 (Cq), 138.5 (Cq), 140.9 (Cq), 141.2 (Cq), 145.2 (C1′), 174.5 (C12), 174.9 (C14). HRMS (APCI) calculated for C_26_H_18_ClN_2_O_4_ [M^+^ + H] 457.0955: found 457.0968.

#### 5.4.21. (*E*)-13-(4-Methoxyphenyl)-9-(2-nitrovinyl)-9,10-dihydro-9,10-[3,4]epipyrroloanthracene-12,14-dione (**16c**)

Compound **16c** was prepared from (*E*)-9-(2-nitrobut-1-en-1-yl)anthracene (0.26 g, 1 mmol) and 1-(4-methoxyphenyl)-1*H*-pyrrole-2,5-dione (0.243 g, 1.3 mmol) following general procedure 3, and afforded the product as a tan solid, 317 mg (70%), Mp. 240–242 °C. IR*_Vmax_* (KBr): 2988, 2938 (C-H), 1717 (C=O), 1515, 1349. (NO_2_), 1657, 1607 (C=C), 1250 (C-N) cm^−1^. ^1^H NMR (400 MHz, DMSO-*d*_6_) δ 3.51 (d, *J* = 6.71 Hz, 1 H, H11), 3.71 (br. s., 3 H, CH_3_), 3.91 (d, *J* = 7.93 Hz, 1 H, H15), 4.95 (br. s., 1 H, H10), 6.32 (d, *J* = 7.32 Hz, 2 H, H3″, H5″), 6.87 (d, *J* = 7.93 Hz, 2 H, H6″, H2″), 7.23–7.46 (m, 7 H, 7 x ArH), 7.61 (d, *J* = 6.10 Hz, 1 H, 1 x ArH), 8.11 (d, *J* = 13.43 Hz, 1 H, H2′), 8.31 (d, *J* = 14.04 Hz, 1 H, H1′). ^13^C NMR (101 MHz, DMSO-*d*_6_) ppm 44.8 (C10), 47.5 (C11), 47.8 (C15), 49.6 (C9), 55.3 (OCH_3_), 114.1, 123.1, 123.5, 124.0, 124.5, 125.3, 126.5, 126.9, 127.2, 127.3, 127.6, 137.3 (C2′), 138.5, 138.6, 141.0, 141.3, 145.2 (C1′), 159.0 (C4″), 174.9 (C12), 175.3 (C14). HRMS (APCI) calculated for C_27_H_21_N_2_O_5_ [M^+^ + H] 453.1450: found 453.1448.

#### 5.4.22. (*E*)-9-Chloro-10-(2-nitrovinyl)-13-phenyl-9,10-dihydro-9,10-[3,4]epipyrroloanthracene-12,14-dione (**16d**)

Compound **16d** was prepared from (*E*)-9-chloro-10-(2-nitrovinyl)anthracene **12d** (0.28 g, 1 mmol) and phenylmaleimide (0.22 g, 1.3 mmol) as described in general procedure 3, and gave the product as a colourless solid, 69 mg (15%). Mp. 263–264 °C. IR*_Vmax_* (KBr): 3125, 3068 (C-H), 1705 (C=O), 1596 (C=C), 1494, 1453 (C=C), 1528, 1347 (NO_2_) cm^−1^. ^1^H NMR (400 MHz, DMSO-*d*_6_) δ 3.71 (d, *J* = 8.55 Hz, 1 H, H11), 4.16 (d, *J* = 8.55 Hz, 1 H, H15), 6.38–6.49 (m, 2 H, 2 x ArH), 7.34 (br. s., 3 H, 3 x ArH), 7.37–7.52 (m, 6 H, 6 x ArH), 7.73 (d, *J* = 7.32 Hz, 1 H, 1 x ArH), 7.88 (d, *J* = 7.32 Hz, 1 H, 1 x ArH), 8.13 (d, *J* = 14.04 Hz, 1 H, H1′), 8.33 (d, *J* = 14.04 Hz, 1 H, H2′). ^13^C NMR (101 MHz, DMSO-*d*_6_) ppm 48.8 (C10), 49.7 (C11), 53.3 (C15), 70.4 (C9), 122.6 (CH), 123.0 (CH), 123.3 (CH), 123.6 (CH), 126.4 (CH), 127.6 (CH), 127.8 (CH), 127.9 (CH), 128.3 (CH), 128.7 (CH), 128.9 (CH), 129.0, 131.4, 136.2 (CH), 136.9 (C2′), 137.2, 139.7, 139.9, 145.7 (C1′), 171.4 (C12), 173.4 (C14). HRMS (APCI) calculated for C_20_H_18_ClN_2_O_4_ [M^+^ + H] 457.0955: found 457.0946.

#### 5.4.23. (*E*)-13-(4-Fluorophenyl)-9-(2-nitrovinyl)-9,10-dihydro-9,10-[3,4]epipyrroloanthracene-12,14-dione (**16e**)

Compound **16e** was prepared from (*E*)-9-(2-nitrovinyl)anthracene **12a** (0.25 g, 1 mmol) and 1-(4-fluorophenyl)-1*H*-pyrrole-2,5-dione **11m** (0.248 g, 1.3 mmol) following general procedure 3, and afforded the product as a colourless solid, 120 mg (27%), Mp. 242–243 °C. IR*_Vmax_* (KBr): 3099, 2892 (C-H), 1711 (C=O), 1688 (C=C), 1505, 1456 (C=C), 1156 (C-N) cm^−1^. ^1^H NMR (400 MHz, DMSO-*d*_6_) δ 3.55 (dd, *J* = 7.94, 3.05 Hz, 1 H, H11), 3.95 (d, *J* = 8.55 Hz, 1 H, H15), 4.97 (d, *J* = 3.05 Hz, 1 H, H10), 6.47 (dd, *J* = 8.55, 4.88 Hz, 2 H, 2 x ArH), 7.14–7.44 (m, 9 H, 9 x ArH), 7.61 (d, *J* = 7.32 Hz, 1 H, 1 x ArH), 8.12 (d, *J* = 14.04 Hz, 1 H, H1′), 8.33 (d, *J* = 14.04 Hz, 1 H, H2′). ^13^C NMR (101 MHz, DMSO-*d*_6_) ppm 45.2 (C10), 48.1 (C11), 48.4 (C15), 50.1 (C9), 116.0, 115.8, 123.5 (CH), 124.0 (CH), 125.0 (CH), 125.7 (CH), 127.0 (CH), 127.4 (CH), 127.6 (CH), 127.8 (CH), 128.9 (CH), 129.1 (CH), 135.1 (CH), 137.7 (C2′), 138.9, 139.0, 141.3, 141.7, 145.7 (C1′), 162.7, 160.3, 175.1 (C12), 175.5 (C14). HRMS (APCI) calculated for C_26_H_18_FN_2_O_4_ [M^+^ + H] 441.1251: found 441.1240.

#### 5.4.24. (*E*)-9-(2-Nitrovinyl)-13-(3,4,5-trimethoxyphenyl)-9,10-dihydro-9,10-[3,4]epipyrroloanthracene-12,14-dione (**16f**)

Compound **16f** was prepared from (*E*)-9-(2-nitrovinyl)anthracene **12a** (0.25 g, 1 mmol) and 1-(3,4,5-trimethoxyphenyl)-1*H*-pyrrole-2,5-dione **11o** (0.341 g, 1.3 mmol) as outlined in general procedure 3, to obtain the product as a yellow solid, 52 mg (10%), Mp. 270–272 °C. IR_Vmax_ (ATR): 3049, 2964 (C-H), 1707 (C=O), 1660 (C=C), 1457, 1420 (C=C), 1518, 1349 (NO_2_), 1196 (C-N) cm^−1^. ^1^H NMR (400 MHz, CDCl_3_) δ 3.43–3.56 (m, 2 H, H11, H15), 3.71 (s, 6 H, 2 x OCH_3_), 3.78 (s, 3 H, p-OCH_3_), 4.90–5.03 (m, 1 H, H10), 5.58 (s, 2 H, H6″, H2″), 7.27–7.37 (m, 6 H, 6 x ArH), 7.43–7.55 (m, 2 H, 2 x ArH), 7.91 (d, *J* = 14.04 Hz, 1 H, H1′), 8.30 (d, *J* = 14.04 Hz, 1 H, H2′). ^13^C NMR (101 MHz, CDCl_3_) ppm 45.8 (C10), 48.0 (C11), 48.2 (C15), 50.4 (C9), 56.1 (C3″, C5″), 60.7 (C4″), 103.9 (C6″, C2″), 123.3 (CH), 123.4 (CH), 124.6 (CH), 125.9 (CH), 126.4 (CH), 127.2 (CH), 127.4 (CH), 127.7 (CH), 127.9 (CH), 136.7 (C2′), 138.2, 138.5, 140.4, 140.8 (C1′), 144.7, 153.5, 174.3 (C12), 174.9 (C14). HRMS (APCI) calculated for C_29_H_25_N_2_O_7_ [M^+^ + H] 513.1662: found 513.1643.

#### 5.4.25. (*E*)-13-(4-Bromophenyl)-9-(2-nitrovinyl)-9,10-dihydro-9,10-[3,4]epipyrroloanthracene-12,14-dione (**16g**)

Compound **16g** was prepared from (*E*)-9-(2-nitrovinyl)anthracene **12a** (0.25 g, 1 mmol) and 1-(4-bromophenyl)-1*H*-pyrrole-2,5-dione **11n** (0.325 g, 1.3 mmol) as outlined in general procedure 3, to obtain the product as a tan solid, 330 mg (66%), Mp. 267–268 °C. IR*_Vmax_* (KBr): 3059, 2965 (C-H), 1701 (C=O), 1524, 1351 (NO_2_), 1537, 1493 (C=C), 1174 (C-N) cm^−1^. ^1^H NMR (400 MHz, DMSO-*d*_6_) δ 3.55 (dd, *J* = 8.55, 3.05 Hz, 1 H, H11), 3.95 (d, *J* = 8.55 Hz, 1 H, H15), 4.97 (d, *J* = 3.05 Hz, 1 H, H10), 6.42 (d, *J* = 8.55 Hz, 2 H, 2 x ArH), 7.20–7.45 (m, 7 H, 7 x ArH), 7.51–7.65 (m, 3 H, 3 x ArH), 8.12 (d, *J* = 14.04 Hz, 1 H, H1′), 8.32 (d, *J* = 13.43 Hz, 1 H, H2′). ^13^C NMR (101 MHz, DMSO-*d*_6_) ppm 44.8 (C10), 47.7 (C11), 48.0 (C15), 49.7 (C9), 121.6, 123.0 (CH), 123.5 (CH), 124.5 (CH), 125.3 (CH), 126.5 (CH), 127.0 (CH), 127.2 (CH), 127.3 (CH), 128.4 (CH), 130.7, 131.9 (CH), 137.2 (C2′), 138.4, 138.5, 140.8, 141.2, 145.2 (C1′), 174.5 (C12), 174.8 (C14). HRMS (APCI) calculated for C_26_H_18_BrN_2_O_4_ [M^+^ + H] 501.0450: found 501.0468.

#### 5.4.26. Methyl (*E*)-4-(9-(2-nitrovinyl)-12,14-dioxo-9,10-dihydro-9,10-[3,4]epipyrroloanthracen-13-yl)benzoate (**16h**)

Compound **16h** was prepared from (*E*)-9-(2-nitrovinyl)anthracene **12a** (0.25 g, 1 mmol) and methyl 4-(2,5-dioxo-2,5-dihydro-1*H*-pyrrol-1-yl)benzoate **11k** (0.3 g, 1.3 mmol) as outlined in general procedure 3, to obtain the product as a colourless solid, 288 mg (60%), Mp. 254–255 °C. IR*_Vmax_* (KBr): 2989, 2901 (C-H), 1705 (C=O), 1524 (NO), 1459, 1433 (C=C), 1115 (C-N) cm^−1^. ^1^H NMR (400 MHz, DMSO-*d*_6_) δ 3.58 (dd, *J* = 8.24, 3.36 Hz, 1 H, H11), 3.83 (s, 3 H, OCH_3_), 3.97 (d, *J* = 8.55 Hz, 1 H, H15), 4.98 (d, *J* = 3.05 Hz, 1 H, H10), 6.66 (m, *J* = 8.55 Hz, 2 H, H6″, H2″), 7.24–7.34 (m, 5 H, 5 x ArH), 7.39 (td, *J* = 8.24, 4.88 Hz, 2 H, 2 x ArH), 7.62 (d, *J* = 6.71 Hz, 1 H, 1 x ArH), 7.92 (m, *J* = 8.55 Hz, 2 H, H3″, H5″), 8.13 (d, *J* = 14.04 Hz, 1 H, H1′), 8.33 (d, *J* = 14.04 Hz, 1 H, H2′). ^13^C NMR (101 MHz, DMSO-*d*_6_) ppm 44.8 (C10), 47.7 (C11), 48.1 (C15), 49.7 (C9), 52.3 (OCH_3_), 123.1 (CH), 123.5 (CH), 124.5 (CH), 125.3 (CH), 126.5 (CH), 126.6 (CH), 127.0 (CH), 127.2 (CH), 127.4 (CH), 129.5, 129.7 (CH), 135.5, 137.2 (C2′), 138.4, 138.5, 140.8, 141.2, 145.3 (C1′), 165.3 (Ester C=O), 174.4 (C12), 174.8 (C14). HRMS (APCI) calculated for C_28_H_21_N_2_O_6_ [M^+^ + H] 481.1400: found 481.1384.

#### 5.4.27. (*E*)-13-(4-Benzoylphenyl)-9-(2-nitrovinyl)-9,10-dihydro-9,10-[3,4]epipyrroloanthracene-12,14-dione (**16i**)

Compound **16i** was prepared from (*E*)-9-(2-nitrovinyl)anthracene **12a** (0.25 g, 1 mmol) and 1-(4-benzoylphenyl)-1*H*-pyrrole-2,5-dione **11f** (0.36 g, 1.3 mmol) as outlined in general procedure 3, to obtain the product as a yellow solid, 210 mg (40%), Mp. 232–234 °C. IR*_Vmax_* (ATR): 3071, 2966 (C-H), 1708 (C=O), 1660 (C=C), 1458, 1448 (C=C), 1529, 1353 (NO_2_), 1195 (C-N) cm^−1^.m^1^H NMR (400 MHz, DMSO-*d*_6_) δ 3.60 (dd, *J* = 8.55, 3.05 Hz, 1 H, H11), 3.99 (d, *J* = 8.55 Hz, 1 H, H15), 5.00 (d, *J* = 3.05 Hz, 1 H, H10), 6.69 (d, *J* = 8.55 Hz, 2 H, H6″, H2″), 7.22–7.35 (m, 5 H, 5 x ArH), 7.35–7.46 (m, 2 H, 2 x ArH), 7.50–7.60 (m, 2 H, 2 x ArH), 7.60–7.74 (m, 6 H, 6 x ArH), 8.14 (d, *J* = 14.04 Hz, 1 H, H1′), 8.34 (d, *J* = 14.04 Hz, 1 H, H2′).m^13^C NMR (101 MHz, DMSO-*d*_6_) ppm 44.8 (C10), 47.8 (C11), 48.1 (C15), 49.7 (C9), 123.1 (CH), 123.6 (CH), 124.5 (CH), 125.3 (CH), 126.4 (CH), 126.5 (CH), 127.1 (CH), 127.2 (CH), 127.4 (CH), 128.2, 128.6 (CH), 129.6 (CH), 130.2 (CH), 132.9 (CH), 134.9, 136.5, 136.9, 137.2 (C2′), 138.4, 138.5, 140.9, 141.2, 145.3 (C1′), 174.5 (C12), 174.8 (C14), 194.8 (C=O). HRMS (APCI) calculated for C_33_H_23_N_2_O_5_ [M^+^ + H] 527.1607: found 527.1599.

#### 5.4.28. (*E*)-13-(3,5-Dimethoxyphenyl)-9-(2-nitrovinyl)-9,10-dihydro-9,10-[3,4]epipyrroloanthracene-12,14-dione (**16j**)

Compound **16j** was prepared from (*E*)-9-(2-nitrovinyl)anthracene **12a** (0.25 g, 1 mmol) and 1-(3,5-dimethoxyphenyl)-1*H*-pyrrole-2,5-dione **11l** (0.3 g, 1.3 mmol) following general procedure 3. The product was isolated as a colourless solid, 304 mg (63%), Mp. 268–271 °C. IR*_Vmax_* (KBr): 2966, 2960 (C-H), 1714 (C=O), 1601 (C=C), 1531 (NO), 1474, 1457 (C=C), 1159 (C-N) cm^−1^. ^1^H NMR (400 MHz, DMSO-*d*_6_) δ 3.52 (dd, *J* = 8.55, 3.05 Hz, 1 H, H11), 3.64 (s, 6 H, 2 x OCH_3_), 3.92 (d, *J* = 8.55 Hz, 1 H, H15), 4.96 (d, *J* = 3.05 Hz, 1 H, H10), 5.49 (d, *J* = 1.83 Hz, 2 H, H1″, H6″), 6.42–6.49 (m, 1 H, H4″), 7.19–7.45 (m, 7 H, 7 x ArH), 7.61 (d, *J* = 6.71 Hz, 1 H, 1 x ArH), 8.12 (d, *J* = 14.04 Hz, 1 H, H1′), 8.32 (d, *J* = 13.43 Hz, 1 H, H2′). ^13^C NMR (101 MHz, DMSO-*d*_6_) ppm 44.9 (C10), 47.6 (C11), 47.9 (C15), 49.7 (C9), 55.4 (OCH_3_), 100.5 (C2″, C6″), 105.0 (C4″), 123.2 (CH), 123.5 (CH), 124.5 (CH), 125.3 (CH), 126.5 (CH), 126.9 (CH), 127.2 (CH), 127.3 (CH), 133.2, 137.3 (CH), 138.5, 138.6, 140.9, 141.2, 145.2 (C1′), 160.3 (C3″, C5″), 174.5 (C12), 174.9 (C14). HRMS (APCI) calculated for C_28_H_23_N_2_O_6_ [M^+^ + H] 483.1556: found 483.1543.

#### 5.4.29. (*E*)-13-(3-Chlorophenyl)-9-(2-nitrovinyl)-9,10-dihydro-9,10-[3,4]epipyrroloanthracene-12,14-dione (**16k**)

Compound **16k** was prepared from (*E*)-9-(2-nitrovinyl)anthracene **12a** (0.25 g, 1 mmol) and 1-(3-chlorophenyl)-1*H*-pyrrole-2,5-dione **11h** (0.27 g, 1.3 mmol) following general procedure 3. The product was isolated as a tan solid 265 mg (58%), Mp. 258–261 °C. IR*_Vmax_* (ATR): 3069, 2966 (C-H), 1713 (C=O), 1594 (C=C), 1481, 1434 (C=C), 1537, 1351 (NO_2_) cm^−1^. ^1^H NMR (400 MHz, DMSO-*d*_6_) δ 3.57 (dd, *J* = 8.29, 3.32 Hz, 1 H, H11), 3.97 (d, *J* = 8.29 Hz, 1 H, H15), 4.98 (d, *J* = 3.32 Hz, 1 H, H10), 6.41–6.52 (m, 2 H, 2 x ArH), 7.23–7.42 (m, 9 H, 9 x ArH), 7.56–7.65 (m, 1 H, 1 x ArH), 8.13 (d, *J* = 13.68 Hz, 1 H, H1′), 8.34 (d, *J* = 13.68 Hz, 1 H, H2′). ^13^C NMR (101 MHz, DMSO-*d*_6_) ppm 44.8 (C10), 47.7 (C11), 48.1 (C15), 49.7 (C9), 123.1 (CH), 123.6 (CH), 124.5 (CH), 125.3 (CH), 126.4 (CH), 126.4 (CH), 126.5 (CH), 127.0 (CH), 127.2 (CH), 127.3 (CH), 128.7 (CH), 130.6 (CH), 132.8, 132.9, 134.7 (CH), 138.5, 138.5, 140.8, 141.2, 145.3 (C1″), 174.5 (C12), 174.8 (C14). HRMS (APCI) calculated for C_26_H_18_ClN_2_O_4_ [M^+^ + H] 457.0955: found 457.0951.

#### 5.4.30. (*E*)-13-(2-Hydroxyphenyl)-9-(2-nitrovinyl)-9,10-dihydro-9,10-[3,4]epipyrroloanthracene-12,14-dione (**16l**)

Compound **16l** was prepared from (*E*)-9-(2-nitrovinyl)anthracene (0.25 g, 1 mmol) and 1-(2-hydroxyphenyl)-1*H*-pyrrole-2,5-dione (0.245 g, 1.3 mmol) following general procedure 3. The product was isolated as a colourless solid, 175 mg (40%), Mp. 203–206 °C. IR*_Vmax_* (KBr): 3030, 2969 (C-H), 1710 (C=O), 1663 (C=C), 1527 (NO), 1495, 1456 (C=C), 1192 (C-N) cm^−1^. ^1^H NMR (400 MHz, DMSO-*d*_6_) δ 3.55 (dd, *J* = 8.55, 3.05 Hz, 1 H, H11), 3.96 (d, *J* = 8.55 Hz, 1 H, H15), 4.97 (d, *J* = 3.05 Hz, 1 H, H10), 6.41–6.50 (m, 2 H, 2 x ArH), 7.23–7.41 (m, 9 H, 9 x ArH), 7.61 (d, *J* = 7.32 Hz, 1 H, 1 x ArH), 8.12 (d, *J* = 14.04 Hz, 1 H, H1′), 8.32 (d, *J* = 14.04 Hz, 1 H, H2′). ^13^C NMR (101 MHz, DMSO-*d*_6_) ppm 44.8 (C10), 47.7 (C11), 48.1 (C15), 49.7 (C9), 123.1 (CH), 123.6 (CH), 124.5 (CH), 125.3 (CH), 126.4 (CH), 126.5 (CH), 127.0 (CH), 127.2 (CH), 127.3 (CH), 128.2 (CH), 128.7 (CH), 130.6 (CH), 132.8, 132.9, 137.2, 138.5, 138.5, 140.8, 141.2, 145.3 (C1′), 174.5 (C12), 174.8 (C14). HRMS (APCI) calculated for C_26_H_19_N_2_O_5_ [M^+^ + H] 439.1294: found 439.1306.

#### 5.4.31. (*E*)-3-(9-(2-Nitrovinyl)-12,14-dioxo-9,10-dihydro-9,10-[3,4]epipyrroloanthracen-13-yl)phenyl acetate (**16m**)

Compound **16m** was prepared from (*E*)-9-(2-nitrovinyl)anthracene (0.25 g, 1 mmol) and 3-(2,5-dioxo-2,5-dihydro-1*H*-pyrrol-1-yl)phenyl acetate **11i** (0.3 g, 1.3 mmol) following general procedure 3. The product was isolated as a colourless solid, 240 mg (50%), Mp. 246–248 °C. IR*_Vmax_* (ATR): 3085, 3065 (C-H), 1715 (C=O), 1606 (C=C), 1484, 1482 (C=C), 1538, 1338 (NO_2_) cm^−1^. ^1^H NMR (400 MHz, DMSO-*d*_6_) δ 2.25 (s, 3 H, CH_3_), 3.55 (dd, *J* = 8.24, 3.36 Hz, 1 H, H11), 3.95 (d, *J* = 8.55 Hz, 1 H, H15), 4.97 (d, *J* = 3.05 Hz, 1 H, H10), 6.25–6.36 (m, 2 H, C4″, C2″), 7.08–7.15 (m, 1 H, 1 x ArH), 7.21–7.43 (m, 8 H, 8 x ArH), 7.62 (d, *J* = 7.32 Hz, 1 H, 1 x ArH), 8.12 (d, *J* = 13.43 Hz, 1 H, H1′), 8.33 (d, *J* = 14.04 Hz, 1 H, H2′). ^13^C NMR (101 MHz, DMSO-*d*_6_) ppm 20.7 (CH_3_), 44.8 (C10), 47.7 (C11), 48.0 (C15), 49.7 (C9), 120.0 (CH), 122.2 (CH), 123.1 (CH), 123.5 (CH), 123.7 (CH), 124.5 (CH), 125.3 (CH), 126.5 (CH), 127.0 (CH), 127.2 (CH), 127.3 (CH), 128.2, 128.9, 129.6 (CH), 132.2, 137.2 (CH), 138.4, 138.5, 140.9, 141.2, 145.3 (C1′), 150.3 (C3″), 168.8 (Acetate C=O), 174.5 (C12), 174.8 (C14). HRMS (APCI) calculated for C_28_H_21_N_2_O_6_ [M^+^ + H] 481.1388: found 481.1394.

#### 5.4.32. (*E*)-13-(4-Aminophenyl)-9-(2-nitrovinyl)-9,10-dihydro-9,10-[3,4]epipyrroloanthracene-12,14-dione (**16n**)

Compound **16n** was prepared from (*E*)-9-(2-nitrovinyl)anthracene **12a** 0.25 g (1 mmol) and 1-(4-aminophenyl)-1*H*-pyrrole-2,5-dione **11p** 0.244 g (1.3 mmol) following general procedure 3. The product was isolated as a brown solid, 197 mg (45%), Mp. >300 °C. IR*_Vmax_*(ATR): 3070, 2953 (C-H), 1712 (C=O), 1595 (C=C), 1482, 1457 (C=C), 1518, 1349 (NO_2_) cm^−1^. ^1^H NMR (400 MHz, DMSO-*d*_6_) δ 3.53 (dd, *J* = 7.94, 3.05 Hz, 1 H, H11), 3.93 (d, *J* = 8.55 Hz, 1 H, H15), 4.90–5.00 (m, 1 H, H10), 6.42 (s, 2 H, NH_2_), 7.12–7.42 (m, 11 H, 11 x ArH), 7.60 (d, *J* = 7.32 Hz, 1 H, 1 x ArH), 8.10 (d, *J* = 14.04 Hz, 1 H, H1′), 8.30 (d, *J* = 13.43 Hz, 1 H, H2′). ^13^C NMR (101 MHz, DMSO-*d*_6_) ppm 43.6 (C10), 44.7 (C11), 47.9 (C15), 49.7 (C9), 123.0 (CH), 123.5 (CH), 124.5 (CH), 125.2 (CH), 125.3 (CH), 126.5 (CH), 126.8 (CH), 127.2 (CH), 127.4 (CH), 128.2 (CH), 128.9 (CH), 131.3, 134.8 (C1′), 137.2, 138.4, 138.4, 140.8, 141.2, 145.2 (C1′), 174.5 (C12), 174.9 (C14). HRMS (APCI) calculated for C_26_H_20_N_3_O_4_ [M + H] 438.1454: found 438.1461.

#### 5.4.33. (*E*)-9-(2-Nitroprop-1-en-1-yl)-13-phenyl-9,10-dihydro-9,10-[3,4]epipyrroloanthracene-12,14-dione (**17a**)

Compound **17a** was prepared from (*E*)-9-(2-nitroprop-1-en-1-yl)anthracene **12b** (0.26 g, 1 mmol) and phenylmaleimide (0.22 g, 1.3 mmol) following general procedure 3. The product was isolated as a colourless solid, 384 mg (88%), Mp. 263–266 °C. IR*_Vmax_* (KBr): 3045, 2934 (C-H), 1700 (C=O), 1598, 1460 (C=C), 1519 (C=C), 1523, 1390 (NO_2_), 1328 (CH_3_) cm^−1^. ^1^H NMR (400 MHz, DMSO-*d*_6_) δ 1.86 (br. s., 3 H, CH3), 3.38–3.51 (m, 1 H, H11), 3.61 (d, *J* = 7.32 Hz, 1 H, H15), 4.97 (d, *J* = 3.66 Hz, 1 H, H10), 6.33–6.51 (m, 2 H, H6″, H2″), 7.19–7.41 (m, 10 H, 10 x ArH), 7.63 (d, *J* = 7.32 Hz, 1 H, ArH), 8.37 (s, 1 H, H1′). ^13^C NMR (101 MHz, DMSO-*d*_6_) ppm 17.2 (C3′), 44.9 (C11), 47.4 (C15), 50.2 (C9), 50.3 (C10), 123.7 (CH), 123.9 (CH), 124.8 (CH), 125.3 (CH), 125.4 (CH), 126.4 (CH), 126.8 (CH), 127.2 (CH), 127.4 (CH), 128.2 (CH), 128.5 (CH), 128.8 (CH), 131.5 (C1″), 131.8, 138.3 (C1′), 138.6, 140.7, 152.5 (C2′), 175.1 (C14, C12). HRMS (APCI) calculated for C_27_H_21_N_2_O_4_ [M^+^ + H] 437.1501: found 437.1513.

#### 5.4.34. (*E*)-9-(2-Nitrobut-1-en-1-yl)-13-phenyl-9,10-dihydro-9,10-[3,4]epipyrroloanthracene-12,14-dione (**17b**)

Compound **17b** was prepared from (*E*)-9-(2-nitrobut-1-en-1-yl)anthracene **12c** (0.27 g, 1 mmol) and phenylmaleimide (0.22 g, 1.3 mmol) following general procedure 3. The product was isolated as a colourless solid, 368 mg (82%), Mp. 259–263 °C. IR*_Vmax_* (KBr): 2968, 2939 (C-H), 1707 (C=O), 1597 (C=C), 1500, 1456 (C=C), 1519, 1390 (NO_2_), 1464 (CH_2_), 1340 (CH_3_) cm^−1^. ^1^H NMR (400 MHz, CDCl_3_) δ 1.03 (br. s., 3 H, CH_3_), 2.28 (br. s., 2 H, CH_2_), 3.34 (br. s., 2 H, H15, H11), 4.95 (s, 1 H, H10), 6.47 (dd, *J* = 6.41, 2.75 Hz, 2 H, H6″, H2″), 7.19–7.45 (m, 11 H, 11 x ArH), 8.20 (s, 1 H, H1′). ^13^C NMR (101 MHz, CDCl_3_) ppm 9.8 (C4′), 23.9 (C3′), 46.0 (C10), 47.8 (C11), 49.7 (C9), 50.3 (C15), 123.5 (CH), 124.8 (CH), 125.8 (CH), 126.0, 126.2 (CH), 126.7 (CH), 127.1 (CH), 127.8 (CH), 127.9, 128.0 (CH), 128.8 (CH), 129.0 (C1′), 129.1, 129.9, 130.9, 134.2, 137.8, 139.0 (C2′), 174.9 (C12, C14). HRMS (APCI) calculated for C_28_H_21_N_2_O_4_ [M^+^ − H] 449.1507: found 449.1490.

#### 5.4.35. (*E*)-13-(4-Chlorophenyl)-9-(2-nitroprop-1-en-1-yl)-9,10-dihydro-9,10-[3,4]epipyrroloanthracene-12,14-dione (**17c**)

Compound **17c** was prepared from (*E*)-9-(2-nitroprop-1-en-1-yl)anthracene **12b** (0.26 g, 1 mmol) and 1-(4-chlorophenyl)-1*H*-pyrrole-2,5-dione **11c** (0.27 g, 1.3 mmol) following general procedure 3. The product was isolated as a colourless solid, 282 mg (60%), Mp. 298–300 °C. IR*_Vmax_* (KBr): 3003, 2967 (C-H), 1700 (C=O), 1524, 1390 (NO_2_), 1494 (C=C), 1460 (CH_3_), 1198 (C-N), 778.31 (Cl-C) cm^−1^. ^1^H NMR (400 MHz, CDCl_3_) δ 1.96 (br. s., 3 H, CH_3_), 3.35 (br. s., 2 H, H11, H15), 4.94 (br. s., 1 H, H10), 6.44 (d, *J* = 7.32 Hz, 2 H, H6″, H2″), 7.14–7.34 (m, 11 H, 11 x ArH), 8.45 (br. s., 1 H, H1′). ^13^C NMR (101 MHz, CDCl_3_) ppm 18.2 (C3′), 46.0 (C10), 47.7 (C11), 48.6 (C15), 50.6 (C9), 123.4, 124.9, 125.9, 126.9, 127.3, 127.4, 127.9, 128.0, 129.3, 137.2 (C2′), 174.7 (C14, C12). HRMS (APCI) calculated for C_27_H_20_ClN_2_O_4_ [M^+^ + H] 471.1112: found 471.1099.

#### 5.4.36. (*E*)-13-(4-Chlorophenyl)-9-(2-nitrobut-1-en-1-yl)-9,10-dihydro-9,10-[3,4]epipyrroloanthracene-12,14-dione (**17d**)

Compound **17d** was prepared from (*E*)-9-(2-nitrobut-1-en-1-yl)anthracene **12c** (0.26 g, 1 mmol) and 1-(4-chlorophenyl)-1*H*-pyrrole-2,5-dione **11c** (0.27 g, 1.3 mmol) following general procedure 3. The product was isolated as a colourless solid, 212 mg (44%), Mp. 275–279 °C. IR*_Vmax_* (KBr): 3005, 2941 (C-H), 1702 (C=O), 1598 (C=C), 1458, 1429 (C=C), 1520, 1383.10 (NO_2_), 1341 (CH_2_), 1310 (CH_3_), 762 (Cl-C) cm^−1^. ^1^H NMR (400 MHz, DMSO-*d*_6_) δ 0.99 (br. s., 3 H, CH3), 2.17 (br. s., 2 H, CH_2_), 3.42 (br. s., 1 H, H11), 3.62 (d, *J* = 6.10 Hz, 1 H, H15), 4.98 (d, *J* = 3.05 Hz, 1 H, H10), 6.47 (d, *J* = 9.16 Hz, 2 H, H6″, H2″), 7.17–7.48 (m, 10 H, 10 x ArH), 7.64 (d, *J* = 6.71 Hz, 1 H, ArH), 8.19 (s, 1 H, H1′). ^13^C NMR (101 MHz, DMSO-*d*_6_) ppm 8.4 (C3′), 23.4 (C4′), 39.8 (C10), 45.0 (C11), 47.5 (C15), 50.4 (C9), 123.6 (CH), 124.9 (CH), 125.4 (CH), 126.4 (CH), 126.8 (CH), 127.4 (CH), 128.1 (CH), 129.0 (CH), 130.2 (Cq), 133.1 (Cq), 138.8 (Cq), 142.6 (Cq), 157.0 (C2′), 174.8 (C14, C12). HRMS (APCI) calculated for C_28_H_20_ClN_2_O_4_ [M^+^ − H] 483.1117: found 483.1101.

#### 5.4.37. (*E*)-13-(4-Methylphenyl)-9-(2-nitroprop-1-en-1-yl)-9,10-dihydro-9,10-[3,4]epipyrroloanthracene-12,14-dione (**17e**)

Compound **17e** was prepared from (*E*)-9-(2-nitrobut-1-en-1-yl)anthracene **12c** (0.26 g, 1 mmol) and 1-(4-methylphenyl)-1*H*-pyrrole-2,5-dione **11d** (0.243 g, 1.3 mmol) as described in general procedure 3. The product was obtained as a colourless solid, 293 mg (65%), Mp. 276–277 °C. IR*_Vmax_* (KBr): 3071, 3010 (C-H), 1700 (C=O), 1518, 1391 (NO_2_), 1198 (C-N) cm^−1^. ^1^H NMR (400 MHz, DMSO-*d*_6_) δ 1.85 (br. s., 3 H, CH_3_), 2.25 (s, 3 H, CH_3_), 3.41 (dd, *J* = 8.24, 2.75 Hz, 1 H, H11), 3.59 (d, *J* = 7.32 Hz, 1 H, H15), 4.96 (d, *J* = 3.05 Hz, 1 H, H10), 6.30 (d, *J* = 8.54 Hz, 2 H, H5″, H3″), 7.12 (d, *J* = 7.93 Hz, 2 H, H6″, H2″), 7.20–7.43 (m, 7 H, 7 x ArH), 7.63 (d, *J* = 6.71 Hz, 1 H,1 x ArH), 8.35 (s, 1 H, H1′). ^13^C NMR (101 MHz, DMSO-*d*_6_) ppm 21.0 (C3′), 45.4 (C10), 47.8 (C11), 49.9 (C15), 51.0 (C9), 55.0 (OCH_3_), 124.1, 125.2, 125.8, 126.6, 126.8, 127.2, 127.6, 127.7, 129.3, 129.7, 138.5, 139.1, 159.7 (C2′), 175.6 (C12, C14). HRMS (APCI) calculated for C_28_H_23_N_2_O_4_ [M + H] 451.1658: found 451.1666.

#### 5.4.38. (*E*)-13-(4-Methylphenyl)-9-(2-nitrobut-1-en-1-yl)-9,10-dihydro-9,10-[3,4]epipyrroloanthracene-12,14-dione (**17f**)

Compound **17f** was prepared from (*E*)-9-(2-nitrobut-1-en-1-yl)anthracene **12c** (0.26 g, 1 mmol) and 1-(4-methyoxyphenyl)-1*H*-pyrrole-2,5-dione (0.243 g, 1.3 mmol) as described in general procedure 3. The product was obtained as a colourless solid, 251 mg (54%), Mp. 254–256 °C. IR*_Vmax_* (KBr): 3006, 2962 (C-H), 1700 (C=O), 1523, 1387 (NO_2_), 1192 (C-N) cm^−1^. ^1^H NMR (400 MHz, DMSO-*d*_6_) δ 0.99 (br. s., 3 H, CH_2_-CH_3_), 2.17 (br. s., 2 H, CH_2_), 2.25 (s, 3 H, CH_3_), 3.40 (br. s., 1 H, H11), 3.58 (br. s., 1 H, H15), 4.97 (d, *J* = 3.05 Hz, 1 H, H10), 6.30 (d, *J* = 7.93 Hz, 2 H, H5″, H3″), 7.11 (d, *J* = 8.54 Hz, 2 H, H6″, H2″), 7.21–7.47 (m, 7 H, 7 x ArH), 7.63 (d, *J* = 6.71 Hz, 1 H, 1 x ArH), 8.20 (s, 1 H, H1′). ^13^C NMR (101 MHz, DMSO-*d*_6_) ppm 9.5 (C3′), 20.6 (OCH_3_), 23.4 (C4) 45.0 (C11), 47.4 (C15), 50.3 (C9), 109.5, 123.6, 124.8, 125.4, 126.1, 126.3, 126.7, 127.3, 127.5, 128.8, 129.2, 138.1, 138.9, 156.9 (C2′), 175.1 (C12, C14). HRMS (APCI) calculated for C_29_H_25_N_2_O_5_ [M^+^ + H] 465.1814: found 465.1831.

#### 5.4.39. (*E*)-9-Chloro-10-(2-nitroprop-1-en-1-yl)-13-phenyl-9,10-dihydro-9,10-[3,4]epipyrroloanthracene-12,14-dione (**17g**)

Compound **17g** was prepared from (*E*)-9-chloro-10-(2-nitroprop-1-en-1-yl)anthracene **12e** (0.298 g, 1 mmol) and phenylmaleimide (0.224 g, 1.3 mmol) as described in general procedure 3. The product was obtained as a colourless solid, 183 mg (39%), Mp. 268–271 °C. IR*_Vmax_* (ATR): 3072, 2981 (C-H), 1713 (C=O), 1625 (C=C), 1499, 1455 (C=C), 1520, 1332 (NO_2_) cm^−1^. ^1^H NMR (400 MHz, CDCl_3_) δ 1.94 (br. s., 3 H, CH_3_), 3.46 (br. s., 2 H, H11, H15), 6.49 (dd, *J* = 6.10, 3.66 Hz, 2 H, 2 x ArH), 7.14–7.29 (m, 5 H, 5 x ArH), 7.29–7.45 (m, 4 H, 4 x ArH), 7.89 (d, *J* = 7.93 Hz, 1 H, 1 x ArH), 8.01 (d, *J* = 7.32 Hz, 1 H, 1 x ArH), 8.49 (s, 1 H, H1′). ^13^C NMR (101 MHz, CDCl_3_) ppm 17.8 (C3′), 52.0 (C10), 53.4 (C15, C11), 70.1 (C9), 122.8 (CH), 124.0 (CH), 124.2 (CH), 126.1 (CH), 127.9 (CH), 128.1 (CH), 128.2 (CH), 128.4 (CH), 128.9 (CH), 129.0 (CH), 130.8, 136.9, 137.3, 140.8 (C2′), 171.2 (C12, C14). HRMS (APCI) calculated for C_27_H_20_ClN_2_O_4_ [M^+^ + H] 471.1112: found 471.1101.

#### 5.4.40. (*E*)-9-Chloro-10-(2-nitrobut-1-en-1-yl)-13-phenyl-9,10-dihydro-9,10-[3,4]epipyrroloanthracene-12,14-dione (**17h**)

Compound **17h** was prepared from (*E*)-9-chloro-10-(2-nitrobut-1-en-1-yl)anthracene **11f** (0.312 g, 1 mmol) and phenyl maleimide (0.224 g, 1.3 mmol) as described in general procedure 3. The product was obtained as a colourless solid, 184 mg (38%), Mp. 261–262 °C. IR*_Vmax_* (ATR): 3075, 2981, 2942 (C-H), 1712 (C=O), 1623 (C=C), 1498, 1455 (C=C), 1520, 1348 (NO_2_) cm^−1^. ^1^H NMR (400 MHz, CDCl_3_) δ 0.94–1.10 (m, 3 H, CH_3_), 2.26 (dd, *J* = 19.23, 7.02 Hz, 2 H, CH_2_), 3.44 (br. s., 2 H, H11, H15), 6.50 (dd, *J* = 5.80, 3.36 Hz, 2 H, 2 x ArH), 7.20–7.50 (m, 9 H, 9 x ArH), 7.90 (d, *J* = 7.94 Hz, 1 H, 1 x ArH), 8.02 (d, *J* = 7.93 Hz, 1 H, 1 x ArH), 8.21 (s, 1 H, H1′). ^13^C NMR (101 MHz, CDCl_3_) ppm 10.0 (C4′), 24.4 (C3′), 52.2 (C10), 53.5 (C15, C11), 70.1 (C9), 123.0 (CH), 123.9 (CH), 124.1 (CH), 126.1 (CH), 127.7 (CH), 128.1, 128.2 (CH), 128.4, 128.9 (CH), 129.0 (C1′), 130.7, 137.5 (C2′), 171.1 (C12, C14). HRMS (APCI) calculated for C_28_H_22_ClN_2_O_4_ [M^+^ − H] 485.1268: found 485.1248.

#### 5.4.41. (*E*)-13-Benzyl-9-(2-nitrovinyl)-9,10-dihydro-9,10-[3,4]epipyrroloanthracene-12,14-dione (**17i**)

Compound **17i** was prepared from (*E*)-9-(2-nitrovinyl)anthracene **12a** (0.25 g, 1 mmol) and 1-benzyl-1*H*-pyrrole-2,5-dione **11a** (0.24 g, 1.3 mmol) as described in general procedure 3. The product was obtained as a colourless solid 323 mg (74%), Mp 232–236 °C. IRVmax (KBr): 3033 2933 (C-H), 1702 (C=O), 1657 (C=C), 1496, 1464 (C=C), 1533, 1390 (NO_2_), 1464 (CH_2_), 1189 (C-N) cm^−1^. ^1^H NMR (400 MHz, DMSO-*d*_6_) δ 3.47 (d, *J* = 5.49 Hz, 1 H, H11), 3.86 (d, *J* = 7.93 Hz, 1 H, H15), 4.22 (br. s., 2 H, CH_2_), 4.90 (br. s., 1 H, H10), 6.31 (d, *J* = 6.71 Hz, 2 H, H2″, H6″), 7.01–7.39 (m, 10 H, 10 x ArH), 7.57 (d, *J* = 6.10 Hz, 1 H, 1 x ArH), 8.12 (d, *J* = 14.04 Hz, 1 H, H2′), 8.28 (d, *J* = 14.04 Hz, 1 H, H1′). ^13^C NMR (101 MHz, DMSO-*d*_6_) ppm 41.3 (C10), 43.6 (C11), 44.2 (C15), 47.5 (C16), 49.2 (C9), 109.5, 122.9, 123.3, 124.3, 125.3, 126.1, 126.4, 126.8, 127.0, 127.4, 128.2, 134.9, 137.2 (C1′), 138.5, 138.6, 141.4, 141.8, 145.2 (C2′), 175.3 (C12), 175.6 (C14). HRMS (APCI) calculated for C_27_H_21_N_2_O_4_ [M^+^ + H] 437.1501: found 437.1505.

#### 5.4.42. (*E*)-13-Benzyl-9-(2-nitroprop-1-en-1-yl)-9,10-dihydro-9,10-[3,4]epipyrroloanthracene-12,14-dione (**17j**)

Compound **17j** was prepared from (*E*)-9-(2-nitroprop-1-en-1-yl)anthracene **12b** (0.26 g, 1 mmol) and 1-benzyl-1*H*-pyrrole-2,5-dione **11a** (0.24 g, 1.3 mmol) as described in general procedure 3. The product was obtained as a colourless solid, 279 mg (62%), Mp 232–236 °C. IR*_Vmax_* (KBr): 3007, 2960 (C-H), 1692 (C=O), 1586 (C=C), 1631, 1431 (Ar C=C), 1520, 1399 (NO_2_), 1457 (CH_2_), 1362 (CH_3_), 1175 (C-N) cm^−1^. ^1^H NMR (400 MHz, DMSO-*d*_6_) δ 1.80 (br. s., 3 H, CH3), 3.49 (d, *J* = 7.32 Hz, 1 H11), 4.13 (s, 2 H, CH2), 4.37 (d, *J* = 5.49 Hz, 1 H15), 4.88 (d, *J* = 3.05 Hz, 1 H, H10), 6.30 (d, *J* = 6.71 Hz, 2 H, H6′, H2′), 6.97–7.37 (m, 10 H, 10 x ArH), 7.55 (d, *J* = 6.71 Hz, 1 H, ArH), 8.33 (s, 1 H, H1′). ^13^C NMR (101 MHz, DMSO-*d*_6_) ppm 17.3 (C3′), 41.3 (C10), 42.6 (C11), 44.4 (C15), 47.2 (C16), 50.0 (C9), 123.5, 124.7, 125.3, 126.2, 126.3, 126.8, 126.8, 127.1, 127.2, 127.4, 127.6, 128.2, 128.4, 131.5, 132.4, 135.0, 137.9, 138.3, 139.1, 141.2, 152.6 (C2′), 165.2, 165.8, 175.7 (C12), 175.8 (C14). HRMS (APCI) calculated for C_28_H_23_N_2_O_4_ [M^+^ + H] 451.1658: found 451.1671.

#### 5.4.43. (*E*)-13-Benzyl-9-(2-nitrobut-1-en-1-yl)-9,10-dihydro-9,10-[3,4]epipyrroloanthracene-12,14-dione (**17k**)

Compound **17k** was prepared from (*E*)-9-(2-nitrobut-1-en-1-yl)anthracene **12c** (0.26 g, 1 mmol) and 1-benzyl-1*H*-pyrrole-2,5-dione **11a** (0.24 g, 1.3 mmol) as described in general procedure 3. The product was obtained as a colourless solid 232 mg (50%), Mp. 188–199 °C. IR*_Vmax_* (KBr): 3002, 2943 (C-H), 1695 (C=O), 1496, 1456 (C=C), 1518, 1339 (NO_2_), 1400 (CH_2_), 1362 (CH_3_), 1175 (C-N) cm^−1^. ^1^H NMR (400 MHz, DMSO-*d*_6_) δ 0.98 (br. s., 3 H, CH3), 2.15 (br. s., 2 H, CH_2_), 3.52 (br. s., 1 H, H11), 4.20 (d, *J* = 2.44 Hz, 2 H, CH_2_ benzyl), 4.41 (d, *J* = 6.10 Hz, 1 H, H15), 4.93 (d, *J* = 3.05 Hz, 1 H, H10), 6.35 (s, 2 H, H2″, H6″), 7.07–7.32 (m, 10 H, 10 x ArH), 7.59 (d, *J* = 6.10 Hz, 1 H, ArH), 8.21 (s, 1 H, H1′). ^13^C NMR (101 MHz, DMSO-*d*_6_) ppm 9.9 (C4′), 23.9 (C3′) 41.3 (C10), 42.7 (C11), 44.4 (C15), 47.3 (C16), 49.9 (C9), 123.4, 124.7, 125.4, 126.2, 126.3, 126.8, 126.8, 127.2, 127.2, 127.6, 127.6, 128.2, 128.4, 131.5, 132.45, 135.0, 139.4, 157.1 (C2′), 165.2, 165.7, 175.6 (C12, C14). HRMS (APCI) calculated for C_29_H_27_N_2_O_4_ [M^+^ + H] 465.1814: found 465.1795.

#### 5.4.44. (*E*)-9-(2-Nitroprop-1-en-1-yl)-13-(3,4,5-trimethoxybenzyl)-9,10-dihydro-9,10-[3,4]epipyrroloanthracene-12,14-dione (**17l**)

Compound **17l** was prepared from (*E*)-9-(2-nitroprop-1-en-1-yl)anthracene **12c** (0.26 g, 1 mmol) and 1-(3,4,5-trimethoxybenzyl)-1*H*-pyrrole-2,5-dione **11b** (0.36 g, 1.3 mmol) as described in general procedure 3. The product was obtained as yellow crystals, 243 mg (45%), Mp. 227–229 °C. IR*_Vmax_* (KBr): 2961, 2997 (C-H), 1697 (C=O), 1592 (C=C), 1462, 1426 (C=C), 1522, 1330 (NO_2_), 1241, 1103 (C-O), 1126 (C-N) cm^−1^. ^1^H NMR (400 MHz, DMSO-*d*_6_) δ 3.21–3.29 (m, 1 H, H11), 3.45 (br. s., 1 H, H15), 3.60 (s, 3 H, p-OCH3), 3.66 (s, 6 H, 2 x OCH_3_), 3.95 (s, 2 H, CH_2_), 4.82 (d, *J* = 3.05 Hz, 1 H, H10), 6.18 (s, 2 H, H2″, H6″), 6.78–7.05 (m, 3 H, 3 x ArH), 7.10–7.32 (m, 4 H, 4 x ArH), 7.53 (d, *J* = 6.71 Hz, 1 H, ArH), 8.30 (s, 1 H, H1′). ^13^C NMR (101 MHz, DMSO-*d*_6_) ppm 17.6 (C3′), 41.5 (C10), 44.6 (C11), 47.0 (C15), 50.2 (C9), 55.9 (2 x OCH_3_), 59.8 (*p*-OCH_3_), 106.1, 123.6, 124.6, 124.9, 126.3, 126.8, 127.1, 130.9, 138.9, 141.0, 152.4 (C2′), 175.6 (C12), 175.7 (C14). HRMS (APCI) calculated for C_31_H_29_N_2_O_7_ [M^+^ + H] 541.1975: found 541.1953.

#### 5.4.45. (*E*)-9-(2-Nitrobut-1-en-1-yl)-13-(3,4,5-trimethoxybenzyl)-9,10-dihydro-9,10-[3,4]epipyrroloanthracene-12,14-dione (**17m**)

Compound **17m** was prepared from (*E*)-9-(2-nitrobut-1-en-1-yl)anthracene **12b** (0.26 g, 1 mmol) and 1-(3,4,5-trimethoxybenzyl)-1*H*-pyrrole-2,5-dione **11b** (0.36 g, 1.3 mmol) as described in general procedure 3. The product was obtained as a tan powder, 299 mg (54%), Mp. 224–229 °C. IR*_Vmax_* (KBr): 2940, 2836 (C-H), 1700 (C=O), 1593 (C=C), 1508, 1426 (C=C), 1521, 1340 (NO_2_), 1460 (CH_2_), 1401 (CH_3_), 1127 (C-N) cm^−1^. ^1^H NMR (400 MHz, DMSO-*d*_6_) δ 0.93 (br. s., 3 H, CH_3_), 2.09 (br. s., 2 H, CH_2_), 3.23–3.27 (m, 1 H, H11), 3.47 (d, *J* = 7.32 Hz, 1 H, H15), 3.60 (s, 3 H, *p*-OCH_3_), 3.67 (s, 6 H, 2 x OCH_3_), 3.94 (s, 2 H, CH_2_ benzyl), 4.84 (d, *J* = 3.05 Hz, 1 H, H10), 6.18 (s, 2 H, H2″, H6″), 6.93 (d, *J* = 6.10 Hz, 3 H, 3 x ArH), 7.12–7.30 (m, 4 H, 4 x ArH), 7.54 (d, *J* = 6.71 Hz, 1 H, ArH), 8.14 (s, 1 H, H1′). ^13^C NMR (101 MHz, DMSO-*d*_6_) ppm 9.5 (C4′), 23.4 (C3′), 41.5 (C10), 44.6 (C11), 47.1 (C15), 50.0 (C9), 55.9 (OCH_3_ x 2), 59.8 (*p*-OCH_3_), 106.1 (CH), 123.4, 124.7, 124.9, 126.2, 127.0, 127.3, 130.9, 137.1, 139.1, 152.5 (C2′), 156.9 (C4″), 175.6 (C12, C14). HRMS (APCI) calculated for C_32_H_30_N_2_O_7_ [M^+^ + H] 555.2131: found 555.2122.

#### 5.4.46. (*E*)-13-(3,4-Dimethoxyphenethyl)-9-(2-nitrovinyl)-9,10-dihydro-9,10-[3,4]epipyrroloanthracene-12,14-dione (**17n**)

Compound **17n** was prepared from (*E*)-9-(2-nitrovinyl)anthracene **12a** (0.25 g, 1 mmol) and 1-(3,4-dimethoxyphenethyl)-1*H*-pyrrole-2,5-dione **11j** (0.34 g, 1.3 mmol) as described in general procedure 3. The product was obtained as a colourless solid, 393 mg (77%), Mp. 152–156 °C. IR*_Vmax_* (KBr): 2941, 2837 (C-H), 1771 (C=O), 1698 (C=C), 1528 (NO), 1494, 1450 (C=C), 1155 (C-N) cm^−1^. ^1^H NMR (400 MHz, DMSO-*d*_6_) δ 1.62–1.81 (m, 2 H, CH_2_), 3.00–3.19 (m, 2 H, CH_2_), 3.40 (dd, *J* = 8.55, 3.05 Hz, 1 H, H11), 3.71 (s, 3 H, OCH_3_), 3.69 (s, 3 H, OCH_3_), 3.81 (d, *J* = 7.94 Hz, 1 H, H15), 4.91 (d, *J* = 3.05 Hz, 1 H, H10), 6.50 (d, *J* = 7.94 Hz, 1 H, H6″), 6.59 (s, 1 H, H2″), 6.80 (d, *J* = 8.55 Hz, 1 H, H5″), 7.21–7.39 (m, 7 H, 7 x ArH), 7.58 (d, *J* = 7.32 Hz, 1 H, 1 x ArH), 8.14 (d, *J* = 14.04 Hz, 1 H, H1′), 8.32 (d, *J* = 14.04 Hz, 1 H, H2′). ^13^C NMR (101 MHz, DMSO-*d*_6_) ppm 21.0 (CH_2_), 32.1 (CH_2_-N), 44.6 (C10), 47.3 (C11), 47.5 (C15), 49.5 (C9), 55.4 (OCH_3_), 55.5 (OCH_3_), 111.9 (CH), 111.9 (CH), 120.1 (CH), 123.0 (CH), 123.4 (CH), 124.4 (CH), 125.3 (CH), 126.5 (CH), 126.8 (CH), 127.1 (CH), 127.2 (CH), 128.2 (CH), 128.9 (CH), 129.9, 137.4 (C2′), 138.3, 138.5, 141.1, 141.4, 145.2 (C1′), 147.5 (C3″), 148.7 (C4″), 175.2 (C12), 175.5 (C14). HRMS (APCI) calculated for C_30_H_27_N_2_O_6_ [M^+^ + H] 511.1869: found 511.1881.

#### 5.4.47. (*E*)-9-(2-Nitrovinyl)-14-phenyl-9,10-dihydro-9,10-[1,2]epitriazoloanthracene-13,15-dione (**18**)

Compound **18** was prepared from (*E*)-9-(2-nitrovinyl)anthracene **12a** (0.25 g, 1 mmol) and 4-phenyl-3*H*-1,2,4-triazole-3,5(4*H*)-dione **11t** (1.3 mmol) as outlined in general procedure 3, to give the product as a brown solid, 289 mg (68%), Mp. 168–169 °C. IR*_Vmax_* (ATR): 3048, 2998 (C-H), 1730 (C=O), 1625 (C=C), 1555, 1486 (C=C) cm^−1^. ^1^H NMR (400 MHz, CDCl_3_) δ 6.39 (s, 1 H, H10), 7.10 (d, *J* = 7.93 Hz, 2 H, 2 x ArH), 7.34–7.45 (m, 9 H, 9 x ArH), 7.58 (d, *J* = 6.71 Hz, 2 H, 2 x ArH), 7.70 (d, *J* = 14.04 Hz, 1 H, H1′), 8.46 (d, *J* = 14.04 Hz, 1 H, H2′). ^13^C NMR (101 MHz, DMSO-*d*_6_) ppm 60.0 (C10), 67.1 (C9), 123.1 (CH), 124.5 (CH), 125.9 (CH), 128.3 (CH), 128.8 (CH), 129.0 (CH), 129.1 (CH), 130.4, 131.8 (C1′), 135.6, 136.3, 145.4 (C2′), 155.8 (C12/C14), 156.4 (C12/C14). HRMS (APCI) calculated for C_24_H_17_N_4_O_4_ [M^+^ + H] 425.1250: found 425.1251.

#### 5.4.48. (*E*)-10-(2-Nitrovinyl)-9,10-dihydro-9,10-ethanoanthracene-11-carbonitrile (**19a**)

Compound **19a** was prepared from (*E*)-9-(2-nitrovinyl)anthracene **12a** (0.25 g, 1 mmol) and acrylonitrile (3 mmol) as outlined in general procedure 3, to give the product as orange crystals, 91 mg (30%), Mp. 223–225 °C. IR*_Vmax_* (ATR): 3113, 3072, 2952 (C-H), 1658 (C=C), 1485, 1457 (C=C), 1526, 1354 (NO_2_), 1190 (CN) cm^−1^. ^1^H NMR (400 MHz, DMSO-*d*_6_) δ 1.93 (dt, *J* = 12.82, 3.36 Hz, 1 H, H12a), 2.27 (ddd, *J* = 12.67, 10.53, 2.44 Hz, 1 H, H12b), 3.66 (dd, *J* = 10.68, 3.97 Hz, 1 H, H11), 4.60 (s, 1 H, C9), 7.11–7.31 (m, 5 H, 5 x ArH), 7.34–7.49 (m, 3 H, 3 x ArH), 8.04 (d, *J* = 13.43 Hz, 1 H, H1′), 8.33 (d, *J* = 14.04 Hz, 1 H, H2′). ^13^C NMR (101 MHz, DMSO-*d*_6_) ppm 31.2 (C11), 34.0 (C12), 41.7 (C9), 48.9 (C10), 121.0, 123.1 (CH), 123.2 (CH), 123.8 (CH), 124.2 (CH), 126.1 (CH), 126.3 (CH), 127.3 (CH), 127.4 (CH), 138.1 (C1′), 138.7, 139.9, 142.1, 142.5, 144.0 (C2′). HRMS (APCI) calculated for C_19_H_14_N_2_O_2_ [M^+^] 302.1055: found 302.1055.

#### 5.4.49. Ethyl (*E*)-10-(2-nitrovinyl)-9,10-dihydro-9,10-ethanoanthracene-11-carboxylate (**19b**)

Compound **19b** was prepared from (*E*)-9-(2-nitrovinyl)anthracene **12a** (0.25 g, 1 mmol) and ethyl acrylate (1.3 mmol) following the general procedure 3. Purification by column chromatography (hexane: ethyl acetate 9:1) afforded the product as a yellow oil, 63 mg (18%). IR*_Vmax_* (ATR): 3123, 2981 (C-H), 1731 (C=O), 1626 (C=C), 1486, 1443 (C=C), 1510, 1361 (NO_2_), 1120 (CN) cm^−1^. ^1^H NMR (400 MHz, CDCl_3_) δ 1.12 (t, *J* = 7.32 Hz, 3 H, CH_3_), 1.99 (d, *J* = 12.21 Hz, 1 H, H12a), 2.31 (t, *J* = 11.60 Hz, 1 H, H11), 3.01 (dd, *J* = 10.68, 4.58 Hz, 1 H, H12b), 4.00 (dd, *J* = 16.18, 7.63 Hz, 2 H, OCH_2_), 4.43 (br. s., 1 H, H9), 7.11–7.26 (m, 5 H), 7.28–7.42 (m, 3 H), 7.49 (d, *J* = 14.04 Hz, 1 H, H1′), 8.35 (d, *J* = 14.04 Hz, 1 H, H2′). ^13^C NMR (101 MHz, CDCl_3_) ppm 14.0 (C2″, CH_3_), 34.2 (C11), 43.7 (C9), 46.3 (C12), 49.3 (C10), 60.9 (C1″, OCH_2_), 122.2 (CH), 123.6 (CH), 123.6 (CH), 123.9 (CH), 126.0 (CH), 126.0 (CH), 126.8 (CH), 127.1 (CH), 139.0, 140.2 (C1′), 142.0, 142.5 (C2′), 142.8, 142.9, 172.7 (C=O). HRMS (APCI) calculated for C_21_H_20_NO_4_ [M^+^ + H] 350.1392: found 350.1389.

#### 5.4.50. Methyl (*E*)-10-(2-nitrovinyl)-9,10-dihydro-9,10-ethanoanthracene-11-carboxylate (**19c**)

Compound **19c** was prepared from (*E*)-9-(2-nitrovinyl)anthracene **12a** (0.25 g, 1 mmol) and methyl acrylate (1.3 mmol) as described in general procedure 3. Purification by column chromatography (hexane: ethyl acetate 9:1) afforded the product as a gold oil, 50 mg (15%). IR*_Vmax_* (ATR): 3086, 2990 (C-H), 1730 (C=O), 1625 (C=C), 1466, 1444 (C=C), 1555, 1361 (NO_2_), 1120 (CN) cm^−1^. ^1^H NMR (400 MHz, CDCl_3_) δ 1.97 (d, *J* = 12.21 Hz, 1 H, H12a), 2.33 (t, *J* = 11.29 Hz, 1 H, H11), 3.03 (dd, *J* = 10.07, 4.58 Hz, 1 H, H12b), 3.56 (s, 3 H, OCH_3_), 4.43 (br. s., 1 H, H9), 7.05–7.30 (m, 6 H, 6 x ArH), 7.30–7.48 (m, 4 H, 4 x ArH, H1′), 8.34 (d, *J* = 14.65 Hz, 1 H, H2′). ^13^C NMR (101 MHz, CDCl_3_) ppm 34.26 (C11), 43.68 (C9), 46.06 (C12), 49.24 (C10), 52.01 (OCH_3_), 122.19 (CH), 123.58 (CH), 123.73 (CH), 123.92 (CH), 126.05 (CH), 126.81 (CH), 127.13 (CH), 139.01, 140.29 (CH), 141.98, 142.41 (C2′), 142.76, 142.86, 173.20 (C=O). HRMS (APCI) calculated for C_20_H_18_NO_4_ [M^+^ + H] 336.1236: found 336.1233.

#### 5.4.51. (*E*)-11-Cyano-10-(2-nitrovinyl)-9,10-dihydro-9,10-ethanoanthracen-11-yl acetate (**19d**)

Compound **19d** was prepared from (*E*)-9-(2-nitrovinyl)anthracene **12a** (0.25 g, 1 mmol) and 1-cyanovinyl acetate (1.3 mmol) following the general procedure 3. The crude product was purified by flash column chromatography (hexane: ethyl acetate 9:1), and afforded a yellow solid, 127 mg (35%), Mp.168–170 °C. IR*_Vmax_* (ATR): 3062, 2956 (C-H), 1753 (C=O), 1650 (C=C), 1484, 1422 (C=C), 1534, 1351 (NO_2_), 1193 (CN) cm^−1^. ^1^H NMR (400 MHz, CDCl_3_) δ 2.08 (dd, *J* = 14.65, 2.44 Hz, 1 H, H12a), 2.94 (dd, *J* = 14.65, 3.05 Hz, 1 H, H12b), 4.40 (t, *J* = 2.75 Hz, 1 H, C9), 7.22–7.30 (m, 5 H, 5 x ArH), 7.33–7.41 (m, 3 H, 3 x ArH), 7.60 (d, *J* = 14.65 Hz, 1 H, H1′), 8.11 (d, *J* = 14.04 Hz, 1 H, H2′). ^13^C NMR (101 MHz, CDCl_3_) ppm 20.7 (CH_3_), 42.8 (C10), 45.2 (C12), 55.1 (C9), 74.8 (C11), 117.1 (CN), 123.8 (CH), 124.2 (CH), 124.8 (CH), 124.9 (CH), 126.8 (CH), 127.2 (CH), 128.1 (CH), 128.6 (CH), 134.9 (C1′), 135.9, 136.0, 141.8, 142.0, 144.5 (C2′), 168.5 (C=O). HRMS (APCI) calculated for C_21_H_17_N_2_O_4_ [M^+^ + H] 361.1188: found 361.1191.

#### 5.4.52. Dimethyl (*E*)-9-(2-nitrovinyl)-9,10-dihydro-9,10-ethanoanthracene-11,12-dicarboxylate (**19e**)

(*E*)-9-(2-Nitrovinyl)-9,10-dihydro-9,10-[3,4]furanoanthracene-12,14-dione **13a** (150 mg, 0.5 mmol) was heated at reflux in methanol (10 mL) and concentrated hydrochloric acid (2 drops) for 6 h. Following cooling to room temperature and removal of the solvent, the residue was taken up in dichloromethane, washed with 10% NaOH solution, water and dried (anhydrous sodium sulphate). The solvent was evaporated and the residue purified by column chromatography (hexane: ethyl acetate 9:1). Recrystallization from ethanol yielded a white solid 126 mg (32%), Mp. 141–143 °C. IR*_Vmax_* (ATR): 3031, 2948 (C-H), 1746 (C=O), 1662 (C=C), 1467, 1422 (C=C), 1539, 1353 (NO_2_), 1211 (CN) cm^−1^. ^1^H NMR (400 MHz, CDCl_3_) δ 3.30 (d, *J* = 10.99 Hz, 1 H, H11), 3.43 (d, *J* = 10.99 Hz, 1 H, H12), 3.46–3.50 (m, 3 H, OCH_3_), 3.54 (s, 3 H, OCH_3_), 4.71 (s, 1 H, H10), 7.05–7.31 (m, 6 H, 6 x ArH), 7.36 (d, *J* = 7.32 Hz, 1 H, 1 x ArH), 7.41–7.54 (m, 2 H, 1 x ArH, H1′), 8.24 (d, *J* = 14.04 Hz, 1 H, H2′). ^13^C NMR (101 MHz, CDCl_3_) ppm 45.7 (C10), 49.2 (C11), 49.6 (C12), 49.7 (C9), 51.9 (OCH_3_), 52.0 (OCH_3_), 122.6 (CH), 123.3 (CH), 123.9 (CH), 126.0 (CH), 126.3 (CH), 126.5 (CH), 126.8 (CH), 127.4 (CH), 139.2 (C1′), 139.2, 139.6, 141.7, 142.3, 142.7 (C2′), 170.6 (C=O), 170.9 (C=O). HRMS (APCI) calculated for C_22_H_20_NO_6_ [M^+^ + H] 394.1291: found 394.1285.

#### 5.4.53. Diethyl (*E*)-9-(2-nitrovinyl)-9,10-dihydro-9,10-ethanoanthracene-11,12-dicarboxylate (**19f**)

(*E*)-9-(2-nitrovinyl)-9,10-dihydro-9,10-[3,4]furanoanthracene-12,14-dione **13a** (150 mg, 0.5 mmol) was refluxed in ethanol (10 mL) and concentrated hydrochloric acid (2 drops) for 6 h. Following cooling to room temperature and removal of the solvent, the residue was taken up in dichloromethane, washed with 10% NaOH solution, water and dried (anhydrous sodium sulphate). The solvent was evaporated and the residue purified by column chromatography (hexane: ethyl acetate 9:1). Recrystallization from ethanol yielded a white solid, 64 mg (15%), Mp. 151–152 °C. IR*_Vmax_* (ATR): 2980, 2928 (C-H), 1745 (C=O), 1655 (C=C), 1467, 1442 (C=C), 1522, 1347 (NO_2_), 1175 (CN) cm^−1^. ^1^H NMR (400 MHz, CDCl_3_) δ 1.04 (t, *J* = 7.32 Hz, 3 H, CH_3_), 1.14 (t, *J* = 7.02 Hz, 3 H, CH_3_), 3.26 (d, *J* = 10.99 Hz, 1 H, H12), 3.43 (d, *J* = 10.99 Hz, 1 H, H11), 3.79–4.09 (m, 4 H, 2 x OCH_2_), 4.71 (s, 1 H, H10), 7.04–7.30 (m, 6 H, 6 x ArH), 7.35 (d, *J* = 6.71 Hz, 1 H, 1 x ArH), 7.46 (d, *J* = 6.71 Hz, 1 H, 1 x ArH), 7.58 (d, *J* = 14.65 Hz, 1 H, H1′), 8.24 (d, *J* = 14.04 Hz, 1 H, H2′). ^13^C NMR (101 MHz, CDCl_3_) ppm 13.7 (CH_3_), 13.9 (CH_3_), 45.6 (C10), 49.2 (C11), 49.8 (C12), 49.8 (C9), 60.9 (OCH_2_), 61.0 (OCH_2_), 122.6 (CH), 123.2 (CH), 123.8 (CH), 126.1 (CH), 126.2 (CH), 126.5 (CH), 126.7 (CH), 127.3 (CH), 129.8, 139.1 (C1′), 139.5, 139.8, 141.7, 142.6, 142.9 (C2′), 169.9 (C=O), 170.6 (C=O). HRMS (APCI) calculated for C_24_H_24_NO_6_ [M^+^ + H] 422.1604: found 422.1596.

### 5.5. General Procedure 4: Preparation of Triptycene Compounds *(**20a**–**20d**, **20f**)*

(i) Preparation of benzenediazonium-2-carboxylate: A solution of anthranilic acid (5.4 g, 0.04 mmol) and trichloroacetic acid (0.06 g) in tetrahydrofuran (60 mL) in a 250 mL conical flask was stirred and cooled on an ice-water bath. Isoamyl nitrite (10 mL) was added portion-wise over 1 min and the mixture warmed to RT over 1.5 h. The mixture was cooled and the tan solid was washed with ice cold tetrahydrofuran. The yield of air dried benzenediazonium-2-carboxylate was 78–80%. The benzenediazonium-2-carboxylate was washed with toluene and stored in solution with toluene (60 mL). (ii) Preparation of Triptycene compounds. To a boiling solution of the appropriate anthracene derivative (4 mmol) in toluene (60 mL) was slowly added a slurry of benzenediazonium-2-carboxylate (prepared from 5.4 g (0.04 mmol) of anthranilic acid) in toluene over the course of 1 h. The mixture was heated to reflux for 1 h, then cooled and the solvent removed. The residual oil was purified by column chromatography (dichloromethane: hexane 1:1) and recrystallised from dichloromethane-hexane gave pure product.

#### 5.5.1. (*E*)-9-(2-Nitroprop-1-en-1-yl)-9,10-dihydro-9,10-[1,2]benzenoanthracene (**20b**)

Compound **20b** was prepared from (*E*)-9-(2-nitroprop-1-en-1-yl)anthracene **12b** (1.05 g, 4 mmol) and benzenediazonium-2-carboxylate (prepared from anthranilic acid (5.4 g, 0.04 mmol)) according to general procedure 4, yielded the product as a yellow solid, 311 mg (23%), Mp. 226–228 °C. IR*_Vmax_* (ATR): 3071, 3016, (C-H), 1594 (C=C), 1482, 1455 (C=C), 1526, 1328 (NO_2_) cm^−1^. ^1^H NMR (400 MHz, DMSO-*d*_6_) δ 1.97 (s, 3 H, CH_3_), 5.75 (s, 1 H, C10), 6.97–7.14 (m, 6 H, 6 x ArH), 7.42 (d, *J* = 6.71 Hz, 3 H, 3 x ArH), 7.50–7.58 (m, 3 H, 3 x ArH), 8.51 (s, 1 H, H1′). ^13^C NMR (101 MHz, DMSO-*d*_6_) ppm 18.0 (C3′), 52.6 (C10), 54.0 (C9), 122.2 (CH), 124.3 (CH), 124.9 (CH), 125.7 (C1′), 126.8, 145.4, 154.3 (C2′). HRMS (APCI) calculated for C_23_H_17_NO_2_ [M^+^] 339.1259: found 339.1259.

#### 5.5.2. (*E*)-9-(2-Nitrobut-1-en-1-yl)-9,10-dihydro-9,10-[1,2]benzenoanthracene (**20c**)

Compound **20c** was prepared from (*E*)-9-(2-nitrobut-1-en-1-yl)anthracene **12c** (1.1 g, 4 mmol) and benzenediazonium-2-carboxylate (prepared from anthranilic acid (5.4 g, 0.04 mmol)) as outlined in general procedure 4 yielded the product as a colourless solid 453 mg (32%), Mp. 210–211 °C. IR*_Vmax_* (ATR): 3071, 2952 (C-H), 1713 (C=O), 1594 (C=C), 1481, 1459 (C=C), 1527, 1349 (NO_2_) cm^−1^. ^1^H NMR (400 MHz, DMSO-*d*_6_) δ 1.00 (t, *J* = 7.32 Hz, 3 H, CH_3_), 2.25 (q, *J* = 7.12 Hz, 2 H, CH_2_), 5.72 (s, 1 H, C10), 6.99–7.10 (m, 6 H, 6 x ArH), 7.30–7.40 (m, 3 H, 3 x ArH), 7.46–7.53 (m, 3 H, 3 x ArH), 8.28 (s, 1 H, H1′). ^13^C NMR (101 MHz, DMSO-*d*_6_) ppm 9.4 (C4′), 24.1 (C3′), 49.7 (C9), 52.5 (C10), 122.0 (CH), 124.3 (CH), 124.8 (CH), 125.8 (CH), 126.1 (C1′), 142.8, 145.2, 158.9 (C2′). HRMS (APCI) calculated for C_24_H_20_NO_2_ [M^+^ + H] 354.1494: found 354.1490.

#### 5.5.3. (*E*)-9-Chloro-10-(2-nitrovinyl)-9,10-dihydro-9,10-[1,2]benzenoanthracene (**20d**)

Compound **20d** was prepared from (*E*)-9-chloro-10-(2-nitrovinyl)anthracene **12d** (1.13 g, 4 mmol) and benzenediazonium-2-carboxylate (prepared from anthranilic acid (5.4 g, 0.04 mmol)) according to general procedure 4, yielded the product as a colourless solid, 430 mg (30%), Mp. 259–261 °C. IR_Vmax_ (ATR): 3070, 2953 (C-H), 1713 (C=O), 1648 (C=C), 1481, 1481 (C=C), 1540, 1350 (NO_2_) cm^−1^. ^1^H NMR (400 MHz, DMSO-*d*_6_) δ 7.15–7.29 (m, 6 H, 6 x ArH), 7.58–7.69 (m, 3 H, 3 x ArH), 7.72–7.82 (m, 3 H, 3 x ArH), 8.12 (d, *J* = 14.04 Hz, 1 H, H1′), 8.50 (d, *J* = 14.65 Hz, 1 H, H2′). ^13^C NMR (101 MHz, DMSO-*d*_6_) ppm 52.9 (C10), 74.6 (C9), 121.4 (CH), 122.1 (CH), 126.2 (CH), 126.5 (CH), 128.9, 131.7 (C1′), 142.5, 143.4, 148.0 (C2′). HRMS (APCI) calculated for C_22_H_14_NO_2_Cl [M^+^] 359.0713: found 359.0712.

#### 5.5.4. 9-(2-Nitroethyl)-9,10-dihydro-9,10-[1,2]benzenoanthracene (**20e**)

Sodium borohydride (60 mg, 1.6 mmol) was added to a stirred solution of (*E*)-9-(2-nitrovinyl)-9,10-dihydro-9,10-[1,2]benzenoanthracene **20a** (100 mg, 0.3 mmol) in dichloromethane (10 mL) and isopropanol (2 mL). After 24 h at room temperature, the solution was neutralised (1 M HCl) and then extracted with CH_2_Cl_2_, dried (sodium sulphate) and solvent removed *in vacuo* to give the product as a colourless solid, 79 mg (80%), Mp. 185–187 °C. IR*_Vmax_* (ATR): 3069, 2951 (C-H), 1594 (C=C), 1482, 1450 (C=C), 1523, 1349 (NO_2_) cm^−1^. ^1^H NMR (400 MHz, CDCl_3_) **δ** 3.75–3.84 (m, 2 H, CH_2_), 5.25–5.36 (m, 2 H, CH_2_), 5.44 (s, 1 H, H10), 7.02–7.13 (m, 6 H, 6 x ArH), 7.35 (d, *J* = 7.32 Hz, 3 H, 3 x ArH), 7.46 (d, *J* = 6.71 Hz, 3 H, 3 x ArH). ^13^C NMR (101 MHz, CDCl_3_) ppm 25.7 (C1′), 52.3 (C9), 54.3 (C10), 72.8 (C2′), 121.2, 124.0, 125.2, 125.5, 146.5. HRMS (APCI) calculated for C_22_H_17_NO_2_ [M^+^] 327.1259: found 327.1255.

#### 5.5.5. 9-Hydroxy-9,10-dihydro-9,10-[3,4]epipyrroloanthracene-12,14-dione (**21k**)

To a solution of anthrone (0.97 g, 5 mmol) in xylene (10 mL) was added maleimide (0.5 g, 5 mmol). The solution was heated at reflux for 1 h, then was cooled to room temperature, filtered and the precipitate was washed with toluene and hexane. The solid dried at room temperature and was recrystallized from toluene affording the product as a colourless solid, 846 mg (58%), Mp. 253–255 °C. IR*_Vmax_* (ATR): 3474 (O-H), 3125, 2981 (C-H), 1752 (C=O), 1665 (C=C), 1571, 1478 (C=C) cm^−1^. ^1^H NMR (400 MHz, DMSO-*d*_6_) δ 3.12 (d, *J* = 8.55 Hz, 1 H, H11), 3.32 (dd, *J* = 8.24, 2.75 Hz, 1 H, H10), 4.60–4.79 (m, 1 H, H15), 6.64 (s, 1 H, 1 x ArH), 7.13–7.29 (m, 4 H, 4 x ArH), 7.44 (d, *J* = 7.32 Hz, 1 H, 1 x ArH), 7.56 (t, *J* = 6.71 Hz, 2 H, 2 x ArH), 10.76 (br. s., 1 H, NH). ^13^C NMR (101 MHz, DMSO-*d*_6_) ppm 43.5 (C11), 49.0 (C10), 51.5 (C15), 76.6 (C9), 120.7 (CH), 121.2 (CH), 123.5 (CH), 124.3 (CH), 126.0 (CH), 126.2 (CH), 126.3 (CH), 126.4 (CH), 137.6, 140.2, 141.5, 145.1, 176.5 (C=O), 177.7 (C=O). HRMS (APCI) calculated for C_18_H_14_NO_3_ [M^+^ + H] 292.0974: found 292.0974.

#### 5.5.6. (*E*)-((10-Chloroanthracen-9-yl)methylene)hydrazine (**22d**)

To a stirred solution of 10-chloro-9-anthraldehyde (1.16 g, 4.85 mmol) in DCM (20 mL) and ethanol 10 mL, was added dropwise hydrazine (1 g, 20 mmol). After 24 h at RT, the solvent evaporated and the residual solid was recrystallized from dichloromethane/hexane. The product was isolated as an orange solid 1.05 g (85%), Mp. 205–207 °C. IR*_Vmax_* (KBr): 3351 (N-H), 3038, 2904 (C-H), 1621 (C=C), 1483, 1438 (C=C) cm^−1^. ^1^H NMR (400 MHz, DMSO-*d*_6_) δ 7.30 (s, 2 H, NH_2_), 7.58–7.67 (m, 2 H, 2 x ArH), 7.67–7.76 (m, 2 H, 2 x ArH), 8.45 (d, *J* = 8.55 Hz, 2 H, 2 x ArH), 8.63 (d, *J* = 8.55 Hz, 2 H, 2 x ArH), 8.82 (s, 1 H, C1′). ^13^C NMR (101 MHz, DMSO-*d*_6_) ppm 109.6 (C10), 124.3 (CH), 126.2 (CH), 126.3 (CH), 127.0, 127.4 (CH), 127.9, 128.7, 129.4, 135.5 (C1′). HRMS (APCI) calculated for C_15_H_12_N_2_Cl [M^+^ + H] 255.0689: found 255.0684.

#### 5.5.7. (*E*)-3-(12,14-Dioxo-9,10-[3,4]furanoanthracen-9(10*H*)-yl)acrylonitrile (**23a**)

Compound **23a** was prepared from (*E*)-3-(anthracen-9-yl)acrylonitrile **22a** (0.23 g, 1 mmol) and maleic anhydride (0.13 g, 1.3 mmol) according to general procedure 3, to yield the product as a colourless solid, 241 mg (74%), Mp. 215–219 °C. IR*_Vmax_* (KBr): 3054, 2965 (C-H), 2218 (CN), 1863, 1700 (C=O), 1626 (C=C), 1458, 1436, 1403 (C=C), 1235 (C-O) cm^−1^. ^1^H NMR (400 MHz, DMSO*-d*_6_) δ 3.77 (dd, *J* = 8.85, 3.36 Hz, 1 H, H11), 4.06 (d, *J* = 8.55 Hz, 1 H, H15), 4.94 (d, *J* = 3.05 Hz, 1 H, H10), 6.54 (d, *J* = 17.09 Hz, 1 H, H2′), 7.15–7.32 (m, 5 H, 5 x ArH), 7.33–7.47 (m, 2 H, 2 x ArH), 7.55 (d, *J* = 6.10 Hz, 1 H, 1 x ArH), 7.95 (d, *J* = 17.09 Hz, 1 H, H1′). ^13^C NMR (101 MHz, DMSO*-d*_6_) ppm 44.3 (C10), 48.8 (C11), 49.6 (C9), 51.6 (C15), 106.6 (CH), 117.6 (CN), 123.1 (CH), 123.5 (CH), 124.6 (CH), 125.3 (CH), 126.6 (CH), 127.2 (CH), 127.3 (CH), 127.6 (CH), 138.3, 138.6, 140.6, 141.0, 149.2 (C2′), 169.9 (C12), 170.8 (C14). HRMS (APCI) calculated for C_21_H_12_NO_3_ [M^+^ − H] 326.0823: found 326.0817.

#### 5.5.8. (*E*)-3-(12,14-Dioxo-9,10-[3,4]epipyrroloanthracen-9(10*H*)-yl)acrylonitrile (**23b**)

Compound **23b** was prepared from (*E*)-3-(anthracen-9-yl)acrylonitrile **22c** (0.23 g, 1 mmol) and maleimide anhydride (0.13 g, 1.3 mmol) according to general procedure 3, to give the product as a colourless solid, 123 mg (38%), Mp. 289–300 °C. IR*_Vmax_* (KBr): 3238 (N-H), 3064, 2970 (C-H), 2222 (CN), 1717 (C=O), 1636 (C=C), 1482, 1457.43 (C=C), 1341 (C-N) cm^−1^. ^1^H NMR (400 MHz, DMSO-*d*_6_) δ 3.26–3.38 (m, 1 H, H11), 3.63 (d, *J* = 8.54 Hz, 1 H, H15), 4.78 (d, *J* = 3.05 Hz, 1 H, H10), 6.51 (d, *J* = 17.09 Hz, 1 H, H1′), 6.88 (s, 1 H, H10), 7.17–7.27 (m, 3 H, 3 x ArH), 7.27–7.40 (m, 2 H, 2 x ArH), 7.52 (d, *J* = 5.49 Hz, 1 H, ArH), 7.91 (d, *J* = 17.09 Hz, 1 H, H2′), 10.88 (s, 1 H, NH). ^13^C NMR (101 MHz, DMSO-*d*_6_) ppm 44.4 (C10), 48.3 (C11), 48.7 (C15), 48.8 (C9), 106.1, 117.9 (CN), 122.9 (CH), 123.2 (CH), 124.2 (CH), 125.2 (CH), 126.2 (CH), 126.6 (CH), 126.8 (CH), 127.0 (CH), 135.2 (CH), 138.7, 139.0, 141.6, 141.9, 150.3 (C2′), 172.7 (C14*/C12*), 176.8 (C12), 177.1 (C14). HRMS (APCI) calculated for C_21_H_13_N_2_O_2_ [M^+^ − H] 325.0983: found 325.0975.

#### 5.5.9. (*E*)-3-(12,14-Dioxo-13-phenyl-9,10-[3,4]epipyrroloanthracen-9(10*H*)-yl)acrylonitrile (**23c**)

Compound **23c** was prepared from (*E*)-3-(anthracen-9-yl)acrylonitrile **22c** (0.23 g, 1 mmol) and phenylmaleimide (0.22 g, 1.3 mmol) according to general procedure 3, to obtain the product as a colourless solid, 81 mg (20%), Mp. 274–281 °C. IR*_Vmax_* (KBr): 3024, 2940 (C-H), 2221 (CN), 1714 (C=O), 1638, 1500 (C=C), 1595 (C=C), 1383 (CN) cm^−1^. ^1^H NMR (400 MHz, DMSO-*d*_6_) δ 3.51 (dd, *J* = 8.54, 3.05 Hz, 1 H, H11), 3.82 (d, *J* = 8.54 Hz, 1 H, H15), 4.92 (d, *J* = 3.05 Hz, 1 H, H10), 6.38–6.45 (m, 2 H, H6″, H2″), 6.55 (d, *J* = 17.09 Hz, 1 H, H1′), 7.23–7.44 (m, 10 H, 10 x ArH), 7.59 (d, *J* = 5.49 Hz, 1 H, ArH), 7.95 (d, *J* = 17.09 Hz, 1 H, H2′). ^13^C NMR (101 MHz, DMSO-*d*_6_) ppm 45.1 (C10), 47.6 (C11), 47.6 (C15), 52.3 (C9), 106.6 (CN), 123.3 (CH), 123.6 (CH), 124.6 (CH), 125.4 (CH), 126.6 (CH), 126.7 (CH), 127.0 (CH), 127.2 (CH), 127.3 (CH), 128.7 (CH), 129.1 (C1′), 131.8, 138.8, 139.0, 141.3, 141.7, 150.3 (C2′), 174.8 (C12), 175.4 (C14). HRMS (APCI) calculated for C_27_H_17_N_2_O_2_ [M^+^ − H] 401.1296: found 401.1285.

#### 5.5.10. 2-((12,14-Dioxo-9,10-[3,4]furanoanthracen-9(10*H*)-yl)methylene)malononitrile (**23d**)

Compound **23d** was prepared from 2-(anthracen-9-ylmethylene)malononitrile **22a** (0.35 g, 1 mmol) and maleic anhydride (0.13 g, 1.3 mmol) according to general procedure 3, to isolate the product as a colourless solid, 53 mg (15%), Mp. 196–198 °C. IR*_Vmax_* (KBr): 2941, 2970 (C-H), 2231 (CN), 1864, 1776 (C=O), 1623 (C=C), 1551, 1457.45, (C=C), 1078 (C-O) cm^−1^. ^1^H NMR (400 MHz, DMSO-*d*_6_) δ 3.74 (br. s., 1 H, H11), 4.12 (br. s., 1 H, H15), 5.00 (d, *J* = 3.05 Hz, 1 H, H10), 7.20–7.38 (m, 4 H, 4 x ArH), 7.44 (t, *J* = 6.41 Hz, 2 H, 2 x ArH), 7.52–7.65 (m, 2 H, 2 x ArH), 8.80 (br. s., 1 H, H1′). ^13^C NMR (101 MHz, DMSO-*d*_6_) ppm 44.3 (C10), 48.5 (C11), 50.2 (C15), 51.1 (C9), 113.1 (CN), 123.5 (CH), 125.3 (CH), 125.6 (CH), 126.6 (CH), 127.1 (CH), 127.7 (CH), 128.1 (CH), 163.6 (C1′), 170.4 (C14, C12). HRMS (APCI) calculated for C_22_H_11_N_2_O_3_ [M^+^ − H] 351.0775: found 351.0788.

#### 5.5.11. 2-((12,14-Dioxo-9,10-[3,4]epipyrroloanthracen-9(10*H*)-yl)methylene)malononitrile (**23e**)

Compound **23e** was prepared from 2-(anthracen-9-ylmethylene)malononitrile **22a** (0.35 g, 1 mmol) and maleimide (0.13 g, 1.3 mmol) according to general procedure 3, to obtain the product as a colourless solid, 147 mg (42%), Mp. 281–284 °C. IR*_Vmax_* (KBr): 3338 (N-H), 3063, 3010 (C-H), 2239 (CN), 1719 (C=O), 1467, 1457 (C=C), 1336 (C-N) cm^−1^. ^1^H NMR (400 MHz, DMSO-*d*_6_) δ 3.20–3.32 (m, 1 H, H11), 3.67 (br. s., 1 H H15), 4.83 (d, *J* = 2.44 Hz, 1 H, H10), 7.17–7.43 (m, 6 H, 6 x ArH), 7.43–7.69 (m, 2 H, 2 x ArH), 8.84 (br. s., 1 H, H1′), 10.95 (s, 1 H, NH).^13^C NMR (101 MHz, DMSO-*d*_6_) ppm 44.4 (C10), 48.1 (C11), 50.4 (C15), 52.3 (C9), 111.8 (CN), 113.3 (CN), 123.2 (CH), 123.4 (CH), 125.0 (CH), 125.5 (CH), 126.2 (CH), 126.5 (CH), 127.4 (CH), 127.5 (CH), 138.1, 140.9, 165.3 (C1′), 177.1 (C14, C12). HRMS (APCI) calculated for C_22_H_12_N_3_O_2_ [M^+^ − H] 350.0935: found 350.0925.

#### 5.5.12. 2-((12,14-Dioxo-13-phenyl-9,10-[3,4]epipyrroloanthracen-9(10*H*)-yl)methylene)malononitrile (**23f**)

Compound **23f** was prepared from 2-(anthracen-9-ylmethylene)malononitrile **22a** (0.35 g, 1 mmol) and phenylmaleimide (0.22 g, 1.3 mmol) according to general procedure 3, to give the product as a colourless solid, 188 mg (44%), Mp. 255–258 °C. IR*_Vmax_* (KBr): 3039, 2970 (C-H), 2240 (CN), 1712 (C=O), 1597 (C=C), 1470, 1457 (C=C), 1387 (C-N) cm^−1^. ^1^H NMR (400 MHz, DMSO-*d*_6_) δ 3.48 (br. s., 1 H, H11), 3.84 (br. s., 1 H, H15), 4.95 (br. s., 1 H, H10), 6.32–6.54 (m, 2 H, H2″, H6″), 7.20–7.70 (m, 11 H, 11 x ArH), 8.87 (br. s., 1 H, H1′). ^13^C NMR (101 MHz, DMSO-*d*_6_) ppm 44.9 (C10), 47.2 (C11), 49.6 (C15), 52.6 (C9), 113.2 (CN), 123.4 (CH), 125.2 (CH), 125.3 (CH), 125.6 (CH), 126.4 (CH), 126.7 (CH), 127.5 (CH), 127.6 (CH), 128.2, 128.6 (CH), 128.8 (CH), 131.4, 165.0 (C1′), 175.0 (C14, C12). HRMS (APCI) calculated for C_28_H_18_N_3_O_2_ [M^+^ + H] 428.1399: found 428.1382.

#### 5.5.13. (*E*)-9-(Hydrazonomethyl)-9,10-dihydro-9,10-[3,4]epipyrroloanthracene-12,14-dione (**23g**)

Compound **23g** was prepared from (*E*)-(anthracen-9-ylmethylene)hydrazine **22c** (0.25 g, 1 mmol) and maleimide (0.13 g, 1.3 mmol) according to general procedure 3, to yield the product as a colourless solid, 200 mg (63%), Mp. 245–247 °C. IR*_Vmax_* (ATR): 3369 (NH), 3062, 2972 (C-H), 1710 (C=O), 1609 (C=C), 1483, 1457 (C=C), 1213 (C-N) cm^−1^. ^1^H NMR (400 MHz, DMSO-*d*_6_) δ 3.23 (d, *J* = 8.55 Hz, 1 H, H11), 3.44 (d, *J* = 8.55 Hz, 1 H, H15), 4.72 (br. s., 1 H, H10), 6.82 (br. s., 2 H, NH_2_), 7.16 (br. s., 4 H, 4 x ArH), 7.25–7.38 (m, 2 H, 2 x ArH), 7.46 (d, *J* = 5.49 Hz, 1 H, 1 x ArH), 7.53 (d, *J* = 6.10 Hz, 1 H, 1 x ArH), 8.06 (s, 1 H, H1′), 10.75 (br. s., 1 H, NH). ^13^C NMR (101 MHz, DMSO-*d*_6_) ppm 44.7 (C10), 48.6 (C11), 49.8 (C15), 50.2 (C9), 123.6 (CH), 123.9 (CH), 124.9 (CH), 125.7 (CH), 126.1 (CH), 126.2 (CH), 126.3 (CH), 137.9 (C1′), 139.1, 140.3, 142.0, 144.0, 177.0 (C12), 177.6 (C14). HRMS (APCI) calculated for C_19_H_16_N_3_O_2_ [M^+^ + H] 318.1243: found 318.1237.

#### 5.5.14. (*E*)-9-(Hydrazonomethyl)-13-phenyl-9,10-dihydro-9,10-[3,4]epipyrroloanthracene-12,14-dione (**23h**)

Compound **23h** was prepared from (*E*)-(anthracen-9-ylmethylene)hydrazine **22c** (0.25 g, 1 mmol) and phenylmaleimide (0.224 g, 1.3 mmol) according to general procedure 3, to give the product as a colourless solid, 171 mg (54%), Mp. 275–277 °C. IR*_Vmax_* (ATR): 3630 (NH), 3072, 2981 (C-H), 1711 (C=O), 1628 (C=C), 1498, 1455 (C=C), 1203 (C-N) cm^−1^. ^1^H NMR (400 MHz, DMSO-*d*_6_) δ 3.44 (d, *J* = 7.94 Hz, 1 H, H11), 3.64 (d, *J* = 7.94 Hz, 1 H, H15), 4.85 (br. s., 1 H, H10), 6.42 (d, *J* = 5.49 Hz, 2 H, 2 x ArH), 6.84 (s, 2 H, NH_2_), 7.15–7.40 (m, 10 H, 10 x ArH), 7.58 (d, *J* = 6.71 Hz, 1 H, 1 x ArH), 7.53 (d, *J* = 6.10 Hz, 1 H, 1 x ArH), 8.09 (s, 1 H, H1′). ^13^C NMR (101 MHz, DMSO-*d*_6_) ppm 45.1 (C10), 47.6 (C11), 48.9 (C15), 50.6 (C9), 123.7 (CH), 124.0 (CH), 124.1 (CH), 124.9 (CH), 125.9 (CH), 126.3 (CH), 126.4 (CH), 126.4 (CH), 126.5 (CH), 128.4 (CH), 128.8 (CH), 131.8, 137.5 (C1′), 139.0, 140.1, 141.5, 143.7, 174.7 (C12), 175.5 (C14). HRMS (APCI) calculated for C_19_H_16_N_3_O_2_ [M^+^ + H] 318.1243: found 318.1237.

#### 5.5.15. (*E*)-9-Chloro-10-(hydrazonomethyl)-9,10-dihydro-9,10-[3,4]epipyrroloanthracene-12,14-dione (**23i**)

Compound **23i** was prepared from (*E*)-((10-chloroanthracen-9-yl)methylene)hydrazine **22c** (0.25 g, 1 mmol) and maleimide (0.13 g, 1.3 mmol) according to general procedure 3, yielding the product as a colourless solid 119 mg (34%), Mp. 241–244 °C. IR*_Vmax_* (ATR): 3635 (NH), 3066, 2980 (C-H), 1713 (C=O), 1597 (C=C), 1498, 1455 (C=C), 1185 (C-N) cm^−1^. ^1^H NMR (400 MHz, DMSO-*d*_6_) δ 3.41 (d, *J* = 8.55 Hz, 1 H, H11), 3.63 (d, *J* = 8.55 Hz, 1 H, H15), 6.90 (br. s., 2 H, NH_2_), 7.24–7.36 (m, 4 H, 4 x ArH), 7.40 (d, *J* = 7.32 Hz, 1 H, 1 x ArH), 7.62 (br. s., 2 H, 2 x ArH), 7.74 (d, *J* = 7.32 Hz, 1 H, 1 x ArH), 8.03 (s, 1 H, H1′), 10.91 (br. s., 1 H, NH). ^13^C NMR (101 MHz, DMSO-*d*_6_) ppm 49.2 (C10), 51.5 (C11), 54.1 (C15), 70.7 (C9), 122.0 (CH), 122.6 (CH), 123.6 (CH), 126.7 (CH), 126.8 (CH), 127.0 (CH), 127.4 (CH), 136.7 (C1′), 137.9, 138.7, 141.0, 142.6, 173.9 (C12), 175.6 (C14). HRMS (APCI) calculated for C_19_H_15_ClN_3_O_2_ [M^+^ + H] 352.0853: found 352.0848.

#### 5.5.16. (*E*)-12,14-Dioxo-9,10-[3,4]epipyrroloanthracene-9(10*H*)-carbaldehyde oxime (**23j**)

Compound **23j** was prepared from (*E*)-anthracene-9-carbaldehyde oxime **22e** (0.22 g, 1 mmol) and maleimide (0.13 g, 1.3 mmol) following general procedure 3. The product was obtained as a colourless solid, 137 mg (43%), Mp. 272–273 °C. IR*_Vmax_* (ATR): 3043, 2939 (C-H), 1710 (C=O), 1595 (C=C), 1491, 1456 (C=C), 1192 (C-N) cm^−1^. ^1^H NMR (400 MHz, DMSO-*d*_6_) δ 3.28 (dd, *J* = 8.55, 3.05 Hz, 1 H, H11), 3.54 (d, *J* = 8.55 Hz, 1 H, H15), 4.77 (d, *J* = 3.05 Hz, 1 H, H10), 7.06–7.26 (m, 4 H, 4 x ArH), 7.33 (d, *J* = 4.88 Hz, 2 H, 2 X ArH), 7.45–7.62 (m, 2 H, 2 x ArH), 8.44 (s, 1 H, H1′), 10.83 (s, 1 H, NH), 11.49 (s, 1 H, =N-OH). ^13^C NMR (101 MHz, DMSO-*d*_6_) ppm 44.7 (C10), 48.4 (C11), 49.4 (C9), 49.9 (C15), 123.6 (CH), 124.1 (CH), 125.1 (CH), 125.9 (CH), 126.3 (CH), 126.6 (CH), 126.6 (CH), 139.0, 139.3, 141.8, 142.9, 147.5 (C1′), 177.0 (C12), 177.5 (C14). HRMS (APCI) calculated for C_19_H_15_N_2_O_3_ [M^+^ + H] 319.1083: found 319.1077.

#### 5.5.17. (*E*)-12,14-Dioxo-13-phenyl-9,10-[3,4]epipyrroloanthracene-9(10*H*)-carbaldehyde oxime (**23k**)

Compound **23k** was prepared from (*E*)-anthracene-9-carbaldehyde oxime **22e** (0.22 g, 1 mmol) and phenylmaleimide (0.22 g, 1.3 mmol) following general procedure 3, The product was obtained as a colourless solid, 260 mg (66%), Mp. 238–242 °C. IR*_Vmax_* (ATR): 3380 (OH), 3053, 2972 (C-H), 1760 (C=O), 1598 (C=C), 1483, 1457 (C=C), 1210 (C-N) cm^−1^. ^1^H NMR (400 MHz, DMSO-*d*_6_) δ 3.48 (dd, *J* = 8.55, 3.05 Hz, 1 H, H11), 3.75 (d, *J* = 8.55 Hz, 1 H, H15), 4.90 (d, *J* = 3.05 Hz, 1 H, H10), 6.34–6.51 (m, 2 H, 2 x ArH), 7.14–7.43 (m, 9 H, 9 x ArH), 7.50–7.65 (m, 2 H, 2 x ArH), 8.49 (s, 1 H, H1′), 11.51 (s, 1 H, =N-OH). ^13^C NMR (101 MHz, DMSO-*d*_6_) ppm 45.0 (C10), 47.3 (C11), 49.0 (C9), 49.9 (C15), 123.7 (CH), 124.3 (CH), 125.1 (CH), 126.1 (CH), 126.5 (CH), 126.5 (CH), 126.7 (CH), 126.8 (CH), 128.5 (CH), 128.8 (CH), 131.7, 138.8, 139.0, 141.3, 142.5, 147.3 (C1′), 174.7 (C12), 175.4 (C14). HRMS (APCI) calculated for C_25_H_19_N_2_O_3_ [M^+^ + H] 395.1396: found 395.1390.

### 5.6. Stability Study of Compound ***16a***

The HPLC stability studies for compound **16a** were performed using a Symmetry^®^ column (C_18_, 5 µm, 4.6 × 150 mm), detector Waters 2487 Dual Wavelength Absorbance, HPLC pump: Waters 1525 binary and Autosampler: Waters 717 plus (Waters Corporation, Milford, MA, USA). Samples were detected at wavelength of 254 nm. The chromatography was performed with the following optimised conditions: mobile phase: (acetonitrile (80%): water (20%)), run time: 10 min, flow rate: 1 mL/min, with detection at 254 nm. Compound **16a** (5 mg) was dissolved in the mobile phase (10 mL) to provide the stock solution for the experiment. The phosphate buffers were prepared following the British Pharmacopoeia monograph 2019 at the selected pH 4, pH 7.4 and pH 9. To a volume of the appropriate buffer (1 mL), stock solution (30 μL) was added, and the solution was shaken and then used immediately for the experiment. Samples of the solution were then analysed over the following 48 h period at selected time intervals (*t* = 0 min, 5 min, 30 min, 60 min, 90 min, 120 min, 24 h and 48 h).

### 5.7. Molecular Modelling

Using MOE (Molecular Operating Environment) 2016.V8 [71], the structures of interest compounds **13j**, **16a**, **16b** and **19a** were flexibly aligned with maprotiline **1** for structural comparison. **13j**, **16a**, **16b** and **19a** (displayed as yellow in their respective overlays) were flexibly aligned with the lead compound Maprotiline **1** (cyan), Figure 8. The molecular structures were processed using the MMFF94s force field, commonly used for small molecule modelling. Flexible alignment was conducted on each compound at 1000 iterations per run. The chirality of the stereogenic centres of the compounds was not defined. Default parameters were utilised for other settings. The proposed alignments featured as the top ranked alignment of generated poses, ranked in order of ascending S score (flexible alignment score comprising of both molecular strain energy and configuration similarity inputs).

### 5.8. X-ray Crystallography

Data for samples **16c**, **16j**, **17l**, **17n**, **19a** and **20a** were collected on a Bruker APEX DUO Kappa system with Mo Kα (λ = 0.71073 Å). Samples were mounted on a MiTeGen microloop and data collected at 100(2) K using an Oxford Instruments Cobra low temperature device. Bruker APEX [72] software was used for collection and reducing data and determination of the space group. Structures were solved employing direct methods (XT [73]) and subsequently refined using least squares minimization procedures (XL [74]) in Olex2 [75]. SADABS [76] was used to apply absorption corrections. Details of the crystal data, data collection and refinement are presented in Table S1. The trimethoxyphenyl group are disordered in **17l**. The phenyl ring was modelled in two positions with the ipso carbon constrained by EXYZ/EADP. Occupancies are 65:35%. Two of the methoxy groups were further disordered and modelled in three positions in total. O30/O30c were constrained to the same position using EXYZ/EADP with O30/C31 34%; O30c/C31c 31% and O30b/C31b 34% occupied and restrained with DFIX, SIMU. O32/C32 34%; O32c/C32c 32%; O32b/C32b 34% occupied and restrained with DFIX and SIMU. The disordered trimethoxyphenyl atoms were restrained by SIMU. In **17n**, the diffuse contribution of approximately 4 MeOH molecules per unit cell have been removed from the overall scattering by using SQUEEZE/PLATON. [77] CCDC 1938150–1938155 contains the supplementary crystallographic data for this paper. These data can be obtained free of charge from The Cambridge Crystallographic Data Centre via www.ccdc.cam.ac.uk/data_request/cif.

### 5.9. Biochemistry

#### 5.9.1. Materials

The DG-75 BL cell line was kindly provided by Dr. Dermot Walls (School of Biotechnology, Dublin City University, Ireland). The MUTU-I (c179) cell line was provided by Professor Martin Rowe, (Division of Cancer Studies, The University of Birmingham, Birmingham, UK). alamarBlue was obtained from BioSource, Belgium and Fetal Bovine Serum (FBS) was sourced from Invitrogen, U.K. RPMI 1640 medium, HEPES and sodium pyruvate were sourced from Biosciences, Ireland. Cell culture consumables were purchased from Greiner Bio-One Ltd., U.K., while all reagents used were obtained from Sigma-Aldrich, Arklow, Ireland.

#### 5.9.2. Cell Culture

The DG-75 Burkitt’s lymphoma cell line used in these experiments is a B-lymphocyte cell line which is derived from a metastatic pleural effusion (lung) isolated from a sporadic case of Burkitt’s lymphoma. The MUTU-I (c179) cell line is an isogenic stable group I BL cell line derived from a BL biopsy. MUTU-I and chemoresistant DG-75 cell lines were cultured in RPMI 1640 (Glutamax) medium which contains phenol red and is supplemented with 10% (*v*/*v*) foetal bovine serum (FBS), L-glutamine (2 mM) and 50 µg/mL penicillin/streptomycin. The MUTU-I cell line is supplemented with alpha-thioglycerol (5 mM in phosphate buffered saline (PBS) with 20 μM bathocuprione disulfonic acid), sodium pyruvate (100 mM) and HEPES (1 mM). The cells were grown in a humidified environment (95% O_2_ with 5% CO_2_) and passaged twice weekly as required depending on their levels of confluency.

#### 5.9.3. AlamarBlue Viability Assay

BL cells were seeded in a 96-well plate (1–5 × 10^4^ cells per 200 µL in well). Cells were treated with the desired drug concentration for a predetermined time frame and incubated as required. alamarBlue (20 µL) was added to each well and the cells were incubated at 37 °C in the dark for 4 h. Plates were then recorded on a fluorescence plate reader (SpectraMax Gemini, Molecular Devices) at 544 nm (excitement) and 590 nm (emission). Experiments were performed in triplicate using controls of reagent, medium and vehicle (100% viability). Taxol (10 μM) was used as the control drug (90% cytotoxicity) for both cell lines. The experiments were performed in triplicate and the mean value for three independent experiments was calculated.

#### 5.9.4. Flow Cytometry

BL cells DG-75 and MUTU-1 (750,000) were treated with the specified concentration of the selected compound and incubated for 24 h (MUTU-1) or 48 h (DG-75) with taxol as the positive control. Samples were harvested by centrifugation and the pellets were rinsed with 0.5 mL of Ca^2+^ Annexin V binding buffer (0.1 M HEPES, pH 7.4; 1.4 M NaCl; 25 mM CaCl_2_) each and pelleted again by centrifugation. Supernatants were decanted and the pellets were suspended again in 50 µL FITC Annexin V (diluted 1:33 in Ca^2+^ Annexin V binding buffer), the samples were then placed on ice and incubated for 10 min in the dark. After the incubation period 0.5 mL of Ca^2+^ Annexin V binding buffer was added and samples were pelleted by centrifugation. The supernatant was decanted and the pellet was suspended in PI solution (500 µL; 0.5 µg/mL). After 1 h, the samples were recorded using a CyAn ADP flow cytometer (Beckman Coulter Inc., Brea, CA, USA). 10,000 cells were analysed with the Flowjo software package. Taxol was used as a positive control for cell death.

#### 5.9.5. Cytotoxicity Assay

The CytoTox 96 non-radioactive cytotoxicity assay (Promega Corporation; 2800 Woods Hollow Road, Madison, WI, USA) was used to determine the cyctotoxicity of selected compounds. BL cells were incubated for 24 hr and then treated with the selected compounds (at 10 μM concentration) **15**, **16b** and **16c** as in the cell viability assay. After 24 or 48 h as appropriate, the lysis solution (10X), (20 μL) was added. The cells were incubated for a further 1 h to ensure complete death. The supernatant (50 μL) was removed and transferred to a new 96-well plate. “Substrate mix″ (50 μL) was added and the plate was retained in the dark at RT for 30 min. “Stop solution″ (50 μL) was added to each well and the absorbance was recorded at 490 nm with a Dynatech MR5000 plate reader and the cell death was calculated.

#### 5.9.6. Generation of Human Peripheral Blood Mononuclear Cells (PBMCs)

Blood was obtained from healthy donors (*n* = 2) after informed consent was received. The blood was then placed into a 50 mL falcon tube and diluted with phosphate-buffered saline (PBS), 1:2. The blood was separated into red blood cells, white blood cell ring and serum using LymphoPrep. The blood was slowly added to Ficoll pague plus (20 mL). The tubes containing the blood were then centrifuged for 30 min at 1700 g. Following transfer of the white blood cell ring to a new 50 mL tube, the volume was adjusted to 50 mL. The samples were again centrifuged for 10 min at 1700 g. The, supernatant liquid was removed; and this step was repeated. The resultant pellet was then resuspended in 10 mL of complete Iscove’s Modified Dulbecco’s (IMDM) media (10% FBS, 0.1% Ciprofloxacin (10 mg/mL)) for use in the cell viability experiments.

#### 5.9.7. Inhibitor Studies: Reactive Oxygen Species

N-Acetylcysteine (Sigma) was dissolved in sterile water (100 mM). Fresh solutions were prepared for each experiment. A 96 well plate was seeded with 2 × 10^4^ cells/well for DG-75 cells. After 23 h, the DG-75 cells were then pre-treated with NAC (5 mM) for 1 h before compound treatment. The wells were then treated with the desired drug concentration (1 µM) for a predetermined time frame (48 h). After treatment and incubation, each well was treated with 20 µL of alamarBlue (37 °C) and the plate was incubated in the dark at 37 °C for 4–6 h. Fluorescence of the 96 well plates was then recorded at 590 nm (excitation 544 nm), as described for ‘alamarBlue viability assay’ (above). The percentage of viable cells remaining with NAC pre-treatment was compared to the percentage of viable cells remaining without NAC pre-treatment in order to determine the implication of ROS in the mechanism of cell death induced.

## Figures and Tables

**Table 1:** Yields and preliminary cell viability data for compounds **13a**–**n**, **15** (Series I) and **14a**–**c** (Series II) in MUTU-1 and DG-75 Burkitt lymphoma cell lines ^a^.

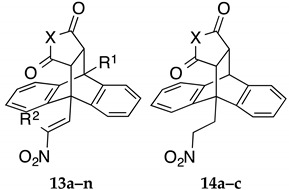					MUTU-1 Cell Viability %		DG-75 Cell Viability %	
Compound	X	R^1^	R^2^	Yield (%)	10 μM	1 μM	10 μM	1 μM
**13a**	O	H	H	80	12.5	24.4	62.2	78.5
**13b**	O	H	CH_3_	72	7.2	11.7	0.47	1.1
**13c**	O	H	CH_2_CH_3_	98	74.1	64.1	80.7	68.0
**13d**	O	Cl	H	15	43.5	35.6	102.2	98.7
**13e**	O	Cl	CH_3_	18	101.1	83.3	98.2	78.2
**13f**	O	Cl	CH_2_CH_3_	55	91.6	85.3	95.1	79.9
**13g**	NH	H	H	50	7.2	0.47	11.7	1.1
**13h**	NH	H	CH_3_	97	82.6	99.7	101.4	106.5
**13i**	NH	H	CH_2_CH_3_	76	73.0	91.1	89.4	96.3
**13j**	NH	Cl	H	25	0.62	0.70	5.9	12.1
**13k**	NH	Cl	CH_3_	50	51.6	83.3	101.4	78.2
**13l**	NH	Cl	CH_2_CH_3_	55	88.3	88.6	102.0	90.0
**13m**	N(CH_3_)	H	H	55	7.4	0	0.93	38.1
**13n**	N(CH_2_)OH	H	H	80	1.8	0	20.4	74.6
**14a**	O	-	-	43	99.3	76.7	102.1	83.0
**14b**	NH	-	-	45	95.0	58.5	101.1	79.3
**14c**	N(C_6_H_5_)	-	-	30	79.2	75.5	81.4	75.7
**15**	Dimer	-	-	10	2.1	4.5	0	3.8
**Maprotiline 8**	-	-	-	-	73.0	93.1	65.1	102.0
**12a**	-	-	-	99	5.9	81.9	14.0	94.6
**Taxol**		-	-	-	9.8	30.4	9.9	48.8

**^a^** Cell proliferation of MUTU-1 and DG-75 cells was determined with an alamarBlue assay (seeding density 1–5 × 10^4^ cells/mL per well for 96-well plates). Compound concentrations of either 1 μM or 10 μM for 24 h (MUTU-1) or 48 h (DG-75) were used to treat the cells (in triplicate) with control wells containing vehicle ethanol (1% *v*/*v*). The mean value for three experiments is shown.

**Table 2:** Yields and preliminary cell viability data for compounds **16a**–**n** (Series IIIA) in MUTU-1 and DG-75 Burkitt lymphoma cell lines.

	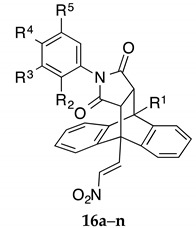		MUTU-1 Cell Viability %		DG-75 Cell Viability %	
Compound	Substitution	Yield (%)	10 μM	1 μM	10 μM	1 μM
**16a**	R^1^=R^2^=R^3^=R^4^=R^5^=H	30	1.4	10.3	1.1	1.1
**16b**	R^1^=R^2^=R^3^=R^5^=H, R^4^=Cl	51	0.38	5.0	0.52	5.5
**16c**	R^1^=R^2^=R^3^=R^5^=H, R^4^=OCH_3_	70	1.1	0.19	2.1	28.4
**16d**	R^1^=Cl, R^2^=R^3^=R^4^=R^5^=H	15	0.41	0.77	5.1	13.5
**16e**	R^1^=R^2^=R^3^=R^5^=H, R^4^=F	27	1.4	0.07	2.3	35.4
**16f**	R^1^=R^2^=H, R^3^=R^4^=R^5^=OCH_3_	10	1.1	1.8	0.46	25.6
**16g**	R^1^=R^2^=R^3^=R^5^=H, R^4^=Br	66	1.1	2.1	0.55	28.2
**16h**	R^1^=R^2^=R^3^=R^5^=H, R^4^=(C=O)OCH_3_	60	0.79	2.3	0.18	20.8
**16i**	R^1^=R^2^=R^3^=R^5^=H, R^4^=(C=O)C_6_H_5_	40	1.2	2.0	2.7	77.5
**16j**	R^1^=R^2^=R^4^=H, R^3^=R^5^=OCH_3_	63	5.4	4.2	0.16	33.5
**16k**	R^1^=R^2^=R^4^=R^5^=H, R^3^=Cl	58	0.84	2.7	0	69.0
**16l**	R^1^=R^3^=R^4^=R^5^=H, R^2^=OH	40	0.61	2.3	0	59.2
**16m**	R^1^=R^2^=R^4^=R^5^=H, R^3^=O(C=O)CH_3_	50	1.2	2.0	0	35.9
**16n**	R^1^=R^2^=R^3^=R^5^=H, R^4^=NH_2_	45	3.7	1.7	0.18	20.8
**Maprotiline 8**	-	-	73.0	93.1	65.1	102.0
**Taxol**	-	-	9.8	30.4	9.9	48.8

Cell proliferation of MUTU-1 and DG-75 cells was determined with an alamarBlue assay (seeding density 1–5 × 10^4^ cells/mL per well for 96-well plates). Compound concentrations of either 1 μM or 10 μM for 24 h (MUTU-1) or 48 h (DG-75) were used to treat the cells (in triplicate) with control wells containing vehicle ethanol (1% *v*/*v*). The mean value for three experiments is shown.

**Table 3:** Yields and preliminary cell viability data for compounds **17a**–**n**, **18** (Series IIIB) in MUTU-1 and DG-75 Burkitt lymphoma cell lines ^a^.

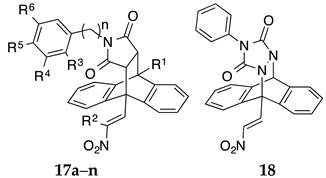				MUTU-1 Cell Viability %		DG-75 Cell Viability %	
Compound	n	Substitution	Yield (%)	10 μM	1 μM	10 μM	1 μM
**17a**	0	R^1^=R^3^=R^4^=R^5^=R^6^=H, R^2^=CH_3_	88	68.6	79.8	88.9	78.3
**17b**	0	R^1^=R^3^=R^4^=R^5^=R^6^=H, R^2^=CH_2_CH_3_	82	83.4	92.6	99.9	96.4
**17c**	0	R^1^=R^3^=R^4^=R^6^=H, R^5^=Cl, R^2^=CH_3_	60	77.6	78.0	86.3	96.6
**17d**	0	R^1^=R^3^=R^4^=R^6^=H, R^5^=Cl, R^2^=CH_2_CH_3_	44	99.9	102.0	78.4	96.6
**17e**	0	R^1^=R^3^=R^4^=R^6^=H, R^5^=OCH_3_, R^2^=CH_3_	65	91.1	84.2	100.4	82.8
**17f**	0	R^1^=R^3^=R^4^=R^6^=H, R^5^=OCH_3_, R^2^=CH_2_CH_3_	54	84.5	86.5	85.1	91.4
**17g**	0	R^1^=Cl, R^3^=R^4^=R^5^=R^6^=H, R^2^=CH_3_	39	78.5	88.0	84.3	85.3
**17h**	0	R^1^=Cl, R^3^=R^4^=R^5^=R^6^=H, R^2^=CH_2_CH_3_	38	96.3	106.7	90.3	89.2
**17i**	1	R^1^=R^3^=R^4^=R^5^=R^6^=R^2^=H	74	0.86	74.9	0.67	93.1
**17j**	1	R^1^=R^3^=R^4^=R^5^=R^6^=H, R^2^=CH_3_	62	85.6	94.6	87.9	103.6
**17k**	1	R^1^=R^3^=R^4^=R^5^=R^6^=H, R^2^=CH_2_CH_3_	50	94.0	80.4	96.0	74.8
**17l**	1	R^1^=R^3^=H, R^4^=R^5^=R^6^=OCH_3_, R^2^=CH_3_	45	77.3	66.8	82.1	81.9
**17m**	1	R^1^=R^3^=H, R^4^=R^5^=R^6^=OCH_3_, R^2^=CH_2_CH_3_	54	66.8	82.2	77.8	82.2
**17n**	2	R^1^=R^3^=R^4^=R^5^=R^6^=R^2^=H	77	2.0	2.8	0.070	19.9
**18**	0	-	68	13.2	75.0	13.2	87.3
**Maprotiline** **8**	-	-	-	73.0	93.1	65.1	102.0
**Taxol**	-	-	-	9.8	30.4	9.9	48.8

**^a^** Cell proliferation of MUTU-1 and DG-75 cells was determined with an alamarBlue assay (seeding density 1–5 × 10^4^ cells/mL per well for 96-well plates). Compound concentrations of either 1 μM or 10 μM for 24 h (MUTU-1) or 48 h (DG-75) were used to treat the cells (in triplicate) with control wells containing vehicle ethanol (1% *v*/*v*). The mean value for three experiments is shown.

**Table 4:** X-ray crystallography data for compounds **16c**, **16j**, **17l**, **17n**, **19a** and **20a**.

Compound	Structure	A^o^	B^o^	C^o^	d_1_ (Å) C-C	d_2_ (Å) C-C
**16c**	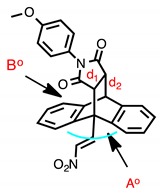	122.05	121.71	-	1.546	1.555
**16j**	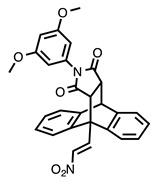	125.99	118.05	-	1.544	1.563
**17l**	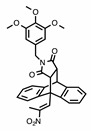	129.35	119.69	-	1.542	1.557
**17n**	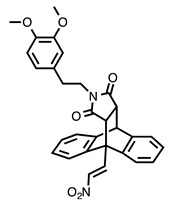	126.73	120.80	-	1.563	1.573
**19a**	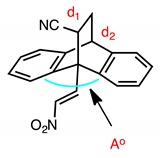	126.94	-	-	1.565	1.556
**20a**	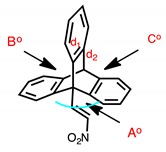	118.50^o^	121.24^o^	120.18^o^	1.400	1.518
**Maprotiline 8**	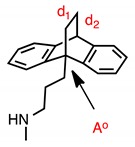	119.46	-	-	1.540	1.546

**Table 5:** Crystal Data and Structure Refinement Parameters for compounds **16c**, **16j**, **17l**, **17n**, **19a** and **20a**.

Compound No.	16c	16j	17l	17n	19a	20a
Empirical formula	C_27_H_20_N_2_O_5_	C_28_H_22_N_2_O_6_	C_31_H_28_N_2_O_6_	C_30_H_26_N_2_O_6_	C_19_H_14_N_2_O_2_	C_22_H_15_NO_2_
Formula weight	452.45	482.47	540.39	510.53	302.32	325.35
F(000)	944.0	1008.0	1136.0	536.0	632.0	680.0
Temperature (K)	100(2)	100(2)	100(2)	100(2)	100(2)	100(2)
Radiation	MoKα	MoKα	MoKα	MoKα	MoKα	MoKα
Crystal size (mm^3^)	0.170 × 0.150 × 0.100	0.190 × 0.160 × 0.120	0.21 × 0.11 × 0.03	0.230 × 0.180 × 0.140	0.270 × 0.140 × 0.070	0.250 × 0.120 × 0.100
Crystal system	monoclinic	monoclinic	monoclinic	triclinic	monoclinic	monoclinic
Space group	P2_1_/c	P2_1_/c	P2_1_/c	P$\bar{1}$	P2_1_/c	Cc
a (Å)	8.3650(3)	14.6412(5)	14.9320(6)	11.2039(3)	15.2934(7)	13.9319(4)
b (Å)	9.8612(4)	8.5186(3)	9.6505(4)	12.0105(3)	6.8232(3)	11.3026(4)
c (Å)	26.0162(11)	18.2016(6)	18.4287(7)	12.7111(3)	15.9060(7)	10.7959(4)
α (°)	90	90	90	65.1220(10)	90	90
β (°)	90.5702(8)	100.5867(12)	97.566(2)	70.7630(10)	117.8513(18)	112.9496(12)
γ (°)	90	90	90	67.6170(10)	90	90
Volume (Å^3^)	2145.94(15)	2231.51(13)	2632.48(18)	1405.31(6)	1467.52(12)	1565.44(9)
Z	4	4	4	2	4	4
ρ_calc_ (g/cm^3^)	1.400	1.436	1.363	1.206	1.368	1.380
μ (mm^−1^)	0.098	0.102	0.097	0.085	0.090	0.089
Reflections collected	48612	90366	66279	60287	114265	53230
Independent reflections	4926	6330	6043	6461	7157	4616
R_int_ =	0.0306	0.0482	0.1354	0.0331	0.0257	0.0200
R_sigma_ =	0.0162	0.0245	0.0827	0.0215	0.0128	0.0111
Data/restraints/parameters	4926/0/308	6330/0/327	6043/419/500	6461/0/345	7157/0/208	4616/2/226
Goodness-of-fit on F^2^	1.014	1.033	1.019	1.017	1.066	1.080
Final R indexes [I ≥ 2σ (I)]	R_1_ = 0.0414, wR_2_ = 0.1057	R_1_ = 0.0421, wR_2_ = 0.0977	R_1_ = 0.0624, wR_2_ = 0.1164	R_1_ = 0.0472, wR_2_ = 0.1227	R_1_ = 0.0359, wR_2_ = 0.1026	R_1_ = 0.0331, wR_2_ = 0.0939
Final R indexes [all data]	R_1_ = 0.0516, wR_2_ = 0.1124	R_1_ = 0.0654, wR_2_ = 0.1099	R_1_ = 0.1372, wR_2_ = 0.1422	R_1_ = 0.0709, wR_2_ = 0.1368	R_1_ = 0.0438, wR_2_ = 0.1080	R_1_ = 0.0353, wR_2_ = 0.0964
Diff. peak/hole (e Å^−3^)	0.37/−0.23	0.42/−0.29	0.66/−0.46	0.27/−0.24	0.59/−0.37	0.45/−0.22
Flack parameter						0.28(17)
CCDC No.	1938150	1938151	1938152	1938153	1938154	1938155

**Table 6:** Yields and preliminary cell viability data for compounds **19a**–**f** (Series IV) in MUTU-1 and DG-75 Burkitt lymphoma cell lines ^a^.

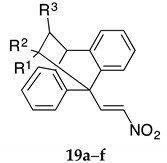			MUTU-1 Cell Viability %		DG-75 Cell Viability %	
Compound	Substitution	Yield (%)	10 μM	1 μM	10 μM	1 μM
**19a**	R^1^=CN, R^2^=R^3^=H	30	0	9.0	0	20.6
**19b**	R^1^=(C=O)OCH_2_CH_3_, R^2^=R^3^=H	18	0.69	48.9	13.4	9.8
**19c**	R^1^=(C=O)OCH_3_, R^2^=R^3^=H	15	8.4	15.6	1.4	92.2
**19d**	R^1^=O(C=O)CH_3_, R^2^=CN, R^3^=H	35	0.69	48.9	1.0	99.4
**19e**	R^1^=R^3^=(C=O)OCH_3_, R^2^=H	32	10.7	84.7	10.3	101.8
**19f**	R^1^=R^3^=(C=O)OCH_2_CH_3_, R^2^=H	15	13.8	13.6	13.8	13.9
**Maprotiline** **8**	-	-	73.0	93.1	65.1	102.0
**Taxol**	-	-	9.8	30.4	9.9	48.8

**^a^** Cell proliferation of MUTU-1 and DG-75 cells was determined with an alamarBlue assay (seeding density 1–5 × 10^4^ cells/mL per well for 96-well plates). Compound concentrations of either 1 μM or 10 μM for 24 h (MUTU-1) or 48 h (DG-75) were used to treat the cells (in triplicate) with control wells containing vehicle ethanol (1% *v*/*v*). The mean value for three experiments is shown.

**Table 7:** Yields and preliminary cell viability data for compounds **20a**–**g** (Series V) in MUTU-1 and DG-75 Burkitt lymphoma cell lines ^a^.

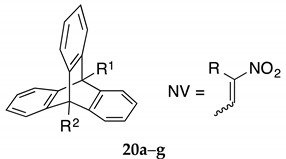					MUTU-1 Cell Viability %		DG-75 Cell Viability %	
Compound	R^1^	R^2^	R	Yield (%)	10 μM	1 μM	10 μM	1 μM
**20a**	H	NV	H	20	1.3	6.0	0.091	90.2
**20b**	H	NV	CH_3_	23	101.8	95.2	100.0	86.4
**20c**	H	NV	CH_2_CH_3_	32	101.8	106.2	84.8	95.1
**20d**	Cl	NV	H	30	0.34	0.95	0	46.0
**20e**	H	CH_2_CH_2_NO_2_	-	80	110.9	98.9	97.2	97.6
**20f**	H	CHO	-	21	5.4	97.9	81.7	72.9
**20g**	H	CH_2_OH	-	85	102.8	101.1	68.7	97.0
**Maprotiline 8**	-	-	-	-	73.0	93.1	65.1	102.0
**Taxol**	-	-	-	-	9.8	30.4	9.9	48.8

**^a^** Cell proliferation of MUTU-1 and DG-75 cells was determined with an alamarBlue assay (seeding density 1–5 × 10^4^ cells/mL per well for 96-well plates). Compound concentrations of either 1 μM or 10 μM for 24 h (MUTU-1) or 48 h (DG-75) were used to treat the cells (in triplicate) with control wells containing vehicle ethanol (1% *v*/*v*). The mean value for three independent experiments is shown.

**Table 8:** Yields and preliminary cell viability data for compounds **21a**–**k** (Series VI) in MUTU-1 and DG-75 Burkitt lymphoma cell lines ^a^.

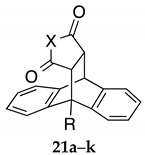							
Compound	R	X	Yield (%)	MUTU-1 Cell Viability %		DG-75 Cell Viability %	
				10 μM	1 μM	10 μM	1 μM
**21a**	H	O	90	93.1	103.2	69.9	91.4
**21b**	H	NH	85	92.8	104.1	74.5	82.0
**21c**	H	N(C_6_H_5_)	90	91.2	93.0	71.3	85.7
**21d**	CHO	O	40	88.4	87.4	64.7	64.8
**21e**	CHO	NH	35	95.1	96.1	71.2	76.0
**21f**	CHO	N(C_6_H_5_)	62	77.7	102.2	72.4	102.0
**21g**	COOH	O	81	88.8	99.2	65.4	93.1
**21h**	COOH	NH	41	92.0	92.6	73.1	86.3
**21i**	COOH	N(C_6_H_5_)	43	92.5	103.4	67.5	73.2
**21j**	OH	O	55	100.4	103.4	93.0	68.0
**21k**	OH	NH	58	100.1	104.0	80.9	65.9
**Maprotiline** **8**	-	-	-	73.0	93.1	65.1	102.0
**Taxol**	-	-	-	9.8	30.4	9.9	48.9

**^a^** Cell proliferation of MUTU-1 and DG-75 cells was determined with an alamarBlue assay (seeding density 1–5 × 10^4^ cells/mL per well for 96-well plates). Compound concentrations of either 1 μM or 10 μM for 24 h (MUTU-1) or 48 h (DG-75) were used to treat the cells (in triplicate) with control wells containing vehicle ethanol (1% *v*/*v*). The mean value for three experiments is shown.

**Table 9:** Yields and preliminary cell viability data for compounds **23a**–**k** (Series VII) in MUTU-1 and DG-75 Burkitt lymphoma cell lines ^a^.

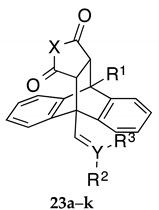							MUTU-1 Cell Viability %		DG-75 Cell Viability %	
Compound	X	Y	R^1^	R^2^	R^3^	Yield (%)	10 μM	1 μM	10 μM	1 μM
**23a**	O	C	H	CN	H	74	82.6	82.7	75.8	89.0
**23b**	NH	C	H	CN	H	38	79.4	79.4	54.1	101.1
**23c**	N(C_6_H_5_)	C	H	CN	H	20	74.1	90.7	89.1	94.4
**23d**	O	C	H	CN	CN	15	82.7	99.8	95.7	103.1
**23e**	NH	C	H	CN	CN	42	83.6	82.9	98.7	85.7
**23f**	N(C_6_H_5_)	C	H	CN	CN	44	71.8	78.9	81.6	82.0
**23g**	NH	N	H	NH_2_	H	63	100.9	95.2	55.0	78.1
**23h**	N(C_6_H_5_)	N	H	NH_2_	H	54	78.0	91.1	87.4	85.8
**23i**	NH	N	Cl	NH_2_	H	34	106.2	102.1	18.9	85.8
**23j**	NH	N	H	OH	H	43	87.6	92.8	63.9	69.1
**23k**	N(C_6_H_5_)	N	H	OH	H	66	83.9	96.9	74.1	81.7
**Maprotiline 8**	-	-	-	-	-	-	73.0	93.1	65.1	102.0
**Taxol**	-	-	-	-	-	-	9.8	30.4	9.9	48.8

**^a^** Cell proliferation of MUTU-1 and DG-75 cells was determined with an alamarBlue assay (seeding density 1–5 × 10^4^ cells/mL per well for 96-well plates). Compound concentrations of either 1 μM or 10 μM for 24 h (MUTU-1) or 48 h (DG-75) were used to treat the cells (in triplicate) with control wells containing vehicle ethanol (1% *v*/*v*). The mean value for three experiments is shown.

**Table 10:** IC_50_ values for selected ethanoanthracenes in the MUTU-I cell line (24 h) *^a^*.

Compound	IC_50_ (µM)	Compound	IC_50_ (µM)	Compound	IC_50_ (µM)
**Taxol**	0.32	**16c**	0.20	**16m**	0.23
**Maprotiline**	15.8	**16d**	0.22	**16n**	0.30
**12a**	2.57	**16e**	0.25	**17n**	0.26
**13j**	0.25	**16f**	0.24	**19a**	0.38
**13m**	0.32	**16g**	0.22	**19c**	0.54
**15**	0.09	**16h**	0.20	**19f**	0.55
**16a**	0.21	**16i**	0.24	**20d**	0.28
**16b**	0.17	**16j**	0.26		

*^a^* Cell proliferation of MUTU-1 cells was determined with an alamarBlue assay (seeding density 1–5 × 10^4^ cells/mL per well for 96-well plates). The compounds (concentration range 0.01 nM to 50 μM) were added (in triplicate) and the cells retained for 24 h, with control wells containing vehicle ethanol (1% *v*/*v*). The values represent the mean for three experiments. IC_50_ value is the concentration required for 50% growth inhibition of MUTU-1 cells.

**Table 11:** IC_50_ values of selected compounds **12a**, **13j**, **15**, **16a**–**d**, **19a** in BL cell lines DG-75 and MUTU-I Burkitt lymphoma cell lines.

Compound	IC_50_ (µM) *^a^*	IC_50_ (µM) *^a^*	*logP ^b^*
	*MUTU-I 24 h*	*DG-75 48 h*	
**12a**	2.57	2.08	4.763
**13j**	0.25	0.78	3.310
**15**	0.09	0.24	5.060
**16a**	0.21	0.71	4.616
**16b**	0.17	0.45	5.269
**16c**	0.20	0.56	4.625
**16d**	0.22	0.59	4.960
**19a**	0.38	0.69	4.275
**Taxol**	0.32	1.32	7.380
**Maprotiline 8**	15.8	37.5	4.602
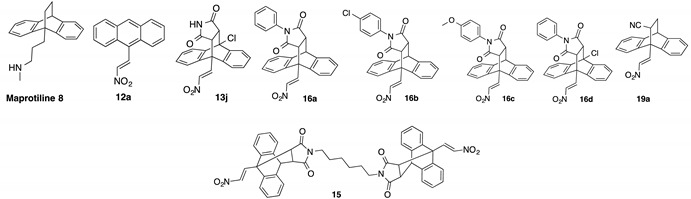			

***^a^*** Cell proliferation of MUTU-1 and DG-75 cells was determined with an alamarBlue assay (seeding density 1–5 × 10^4^ cells/mL per well for 96-well plates). The compounds (concentration range 0.01 nM to 50 μM) were added (in triplicate) and the cells retained for 24 h (MUTU-1) or 48 h (DG-75), with control wells containing vehicle ethanol (1% *v*/*v*). The values represent the mean for three experiments. IC_50_ value is the concentration required for 50% growth inhibition of MUTU-1 cells. ***^b^*** log P values calculated from ChemDraw Ultra, version 12.0. (PerkinElmer, Waltham, MA, USA).

## Supplementary Materials

The following are available online at https://www.mdpi.com/1424-8247/13/1/16/s1, Details of synthesis and spectroscopic characterization data for compounds **11a**–**11n**, **11p**–**11r**, **20a**, **20f**, **20g**, **21a**–**j**, **22a**, **22b**, **22c**, **22e**; ^1^H and ^13^C NMR spectra for compounds **13a**, **16j**, **19a**, **20d** and **23g** (Figures S1–S23); Tier 1 Profiling screen of selected ethanoanthracenes and related compounds, (Table S1); Lipinski properties for selected ethanoanthracenes and related compounds, (Table S2).

## Abbreviations

**Abbreviations**	**Full Name**
AKT	protein kinase B
BL	Burkitt’s lymphoma
C-H COSY	Carbon-Hydrogen Correlation Spectroscopy
CLL	Chronic lymphocytic leukaemia
DEPT	Distortionless Enhancement by Polarization Transfer
EBV	Epstein Barr virus
ER	Estrogen Receptor
HMBC	Heteronuclear Multiple Bond Correlation
LDH	Lactose dehydrogenase
MS	Mass Spectrometry
mTOR	mammalian target of rapamycin
NAC	N-Acetylcysteine
NET	Norepinephrine transporter
NF–κΒ	nuclear factor kappa B
NMR	Nuclear Magnetic Resonance
MOE	Molecular Operating Environment
PBMCs	Peripheral blood mononuclear cells
PBS	Phosphate-buffered saline
PI3K	Phosphatidylinositol 3-kinase
PTP	protein tyrosine phosphatase
PUMA	p53 upregulated modulator of apoptosis
ROS	Reactive oxygen species
RXRα	Retinoid X receptor alpha
SAR	Structure activity relationship
SERT	Serotonin reuptake transporter
SSRI	Selective serotonin reuptake inhibitor
TNFα	tumor necrosis factor alpha
