# Supplementary material for: Design, Synthesis and Biochemical Evaluation of Novel Ethanoanthracenes and Related Compounds to Target Burkitt’s Lymphoma
Source: Pharmaceuticals (Basel). 2020 Jan 17;13(1):16. doi: 10.3390/ph13010016 (PMC7168933; doi:10.3390/ph13010016)
Supplement: Supplementary file 1 [file pharmaceuticals-13-00016-s001.pdf]

pharmaceuticals

Design, Synthesis and Biochemical Evaluation of Novel Ethanoanthracenes and Related Compounds to Target Burkitt's Lymphoma

Andrew J. Byrne [1], Sandra A. Bright [2], James P. McKeown [1], John E. O'Brien [3], Brendan Twamley [3], Darren Fayne [2], D. Clive Williams [2] and Mary J. Meegan [1,*]

1 School of Pharmacy and Pharmaceutical Sciences, Trinity Biomedical Sciences Institute, Trinity College Dublin, 152-160 Pearse St, Dublin 2, D02 R590, Ireland; byrnea40@tcd.ie (A.J.B.); mckeowjp@tcd.ie (J.P.M.)

2 School of Biochemistry and Immunology, Trinity Biomedical Sciences Institute, Trinity College Dublin, 152-160 Pearse St, Dublin 2, D02 R590, Ireland; brights@tcd.ie (S.A.B.); FAYNED@tcd.ie (D.F.); Clive.Williams@tcd.ie (D.C.W.)

3 School of Chemistry, Trinity Biomedical Sciences Institute, Trinity College Dublin, 152-160 Pearse St, Dublin 2, D02 R590, Ireland; jeobrien@tcd.ie (J.E.O.); TWAMLEYB@tcd.ie (B.T.)

* Correspondence: mmeegan@tcd.ie; Tel.: +353-1-896-2798; Fax: +353-1-8962793

Experimental details for preparation and characterisation of compounds 11a-11n, 11p-11r, 20a, 20f, 20g, 21a-j, 22a-c, 22e.

Materials and Methods: Chemistry

All reagents were commercially available and were used without further purification unless otherwise indicated. Tetrahydrofuran (THF) was distilled immediately prior to use from Na/Benzophenone under a slight positive pressure of nitrogen, toluene was dried by distillation from sodium and stored on activated molecular sieves (4Å) and dichloromethane was dried by distillation from calcium hydride prior to use. Uncorrected melting points were measured on a Gallenkamp SMP 11 melting point apparatus. Infra-red (IR) spectra were recorded as thin film on NaCl plates, or as potassium bromide discs on a Perkin Elmer FT-IR Spectum 100 spectrometer. ^{1}H and ^{13}C nuclear magnetic resonance (NMR) spectra were recorded at 27°C on a Brucker Avance DPX 400 spectrometer (400.13 MHz, ^{1}H; 100.61 MHz, ^{13}C) at 20 °C in either $CDCl_3$ (internal standard tetramethylsilane TMS) or CD_3OD by Dr. John O'Brien and Dr. Manuel Ruether in the School of Chemistry, Trinity College Dublin. For $CDCl_3$, ^{1}H-NMR spectra were assigned relative to the TMS peak at δ 0.00 ppm and ^{13}C-NMR spectra were assigned relative to the middle $CDCl_3$ triplet at δ 77.00 ppm. For CD_3OD, ^{1}H and ^{13}C-NMR spectra were assigned relative to the centre peaks of the CD_3OD multiplets at δ 3.30 and 49.00 ppm respectively. Electrospray ionisation mass spectrometry (ESI-MS) was performed in the positive ion mode on a liquid chromatography time-of-flight (TOF) mass spectrometer (Micromass LCT, Waters Ltd., Manchester, UK) equipped with electrospray ionization (ES) interface operated in the positive ion mode at the High Resolution Mass Spectrometry Laboratory by Mr. Brian Talbot in the School of Pharmacy and Pharmaceutical Sciences, Trinity College Dublin and Dr. Martin Feeney in the School of Chemistry, Trinity College Dublin. Mass measurement accuracies of < ±5 ppm were obtained. Low resolution mass spectra (LRMS) were acquired on a Hewlett-Packard 5973 MSD GC-MS system in electron impact (EI) mode. R_f values are quoted for thin layer chromatography on silica gel Merck F-254 plates, unless otherwise stated. Flash column chromatography was carried out on Merck Kieselgel 60 (particle size 0.040-0.063 mm). Chromatographic separations were also carried out on Biotage SP4 instrument. All products isolated were homogenous on TLC. Analytical high-performance liquid chromatography (HPLC) to determine the purity of the final compounds was performed using a Waters 2487 Dual Wavelength Absorbance detector, a Waters 1525 binary HPLC pump, a Waters In-Line Degasser AF and a Waters 717plus Autosampler. The column used was a Varian Pursuit XRs C18 reverse phase 150 x 4.6 mm chromatography column. Samples were detected using a wavelength of 254 nm.

General procedure 1: preparation of phenyl and benzyl maleimides (11a-11n, 11p-11r)

To a solution of maleic anhydride (20 mmol) dissolved in diethyl ether (25 mL) was added the appropriate benzyl or aryl amine (20 mmol) dissolved in diethyl ether (10 mL). The reaction was stirred under reflux at RT for 1 h. The precipitated solid was isolated by filtration and washed with diethyl ether. This solid was immediately used in the next step and placed in a conical flask containing sodium acetate (0.7 g) and acetic anhydride (10 mL). This mixture was heated to 90 °C for 0.5 h, after which the mixture was poured over ice water (100 mL). The precipitated solid was isolated by filtration and recrystallised from ethanol.

1-Benzyl-1H-pyrrole-2,5-dione (11a)

Preparation as described above from maleic anhydride (20 mmol) and benzyl amine (20 mmol) according to general procedure 1. The product was obtained as tan crystals, 1.51 g (40%), Mp. 69-72 °C (lit. M.p. 68-69 °C [1]). IR ν_{max} (KBr): 3039, 2947, 1700 (C=O), 1631 (C=C), 1530, 1496, 1456, 1137 cm^{-1}. ^{1}H NMR (400 MHz, $CDCl_3$) δ 4.64 (s, 2 H, CH_2), 6.66 (s, 2 H, HC=CH), 7.11 - 7.41 (m, 5 H, 5 x ArH). ^{13}C NMR (101 MHz, $CDCl_3$) δ 41.3 (CH_2), 127.7 (CH), 128.1, 128.1, 128.2 (CH) , 128.6 (CH), 128.8, 130.9, 134.1 (HC=CH), 136.1, 170.3 (O=C). HRMS (APCI) calculated for $C_{11}H_{10}NO_2$ [M$^+$+H] 188.0712: found 188.0705.

1-(3,4,5-Trimethoxybenzyl)-1H-pyrrole-2,5-dione (11b)

Preparation as described above from maleic anhydride (20 mmol) and 3,4,5-trimethoxybenzyl amine (20 mmol) according to general procedure 1. The product was obtained as brown crystals, 1.11 g (20%), Mp. 135-138 °C (lit. M.p. 128-130 °C [2]). IR ν_{max} (KBr): 3017, 2990 (C-H), 1709 (C=O), 1592 (C=C), 1508, 1429 (C=C), 1464 (CH_2), 1358 (CH_3), 1122 (C-N) cm^{-1}. ^{1}H NMR (400 MHz, $CDCl_3$) δ 3.75 - 3.85 (m, 9 H, 3 x OCH_3), 4.56 (s, 2 H, CH_2), 6.57 (s, 2 H, HC=CH), 6.69 (s, 2 H, 2 x ArH). ^{13}C NMR (101 MHz, $CDCl_3$) ppm 41.6 (CH_2), 56.0 (m-OCH_3 x 2), 60.7 (p-OCH_3), 76.7, 77.3, 105.6 (CH), 131.8, 134.1 (HC=CH), 137.5, 153.2, 170.3 (C=O). HRMS (APCI) calculated for $C_{14}H_{16}NO_5$ [M$^+$+H] 278.1028: found 278.1025.

1-(4-Chlorophenyl)-1H-pyrrole-2,5-dione (11c)

Preparation as described above from maleic anhydride (20 mmol) and 4-chloroaniline (20 mmol) according to general procedure 1. The product was obtained as yellow crystals, 2.29 g (55%), Mp. 118-124 °C (109-110 °C [3]). IR ν_{max} (KBr): 3116, 3084 (Ar C-H), 1583 (C=C), 1497, 1452 (C=C), 1712 (C=O), 1149 (C-N) cm^{-1}. ^{1}H NMR (400 MHz, $CDCl_3$) δ 6.83 (s, 2 H, HC=CH) 7.29 (d, *J* = 8.54 Hz, 2 H, 2 x ArH) 7.41 (d, *J* = 8.54 Hz, 2 H, 2 x ArH). ^{13}C NMR (101 MHz, $CDCl_3$) ppm 127.0 (CH), 129.2 (CH), 129.7 (s), 133.5 (s), 134.2 (HC=CH), 169.1 (O=C). HRMS (APCI) calculated for $C_{10}H_7ClNO_2$ [M$^+$+H] 208.0165: found 208.0165.

1-(4-Methylphenyl)-1H-pyrrole-2,5-dione (11d)

Preparation as described above from maleic anhydride (20 mmol) and 4-methylaniline (20 mmol) according to general procedure 1. The product was obtained as yellow crystals (40%), Mp 145-155 °C (lit. M.p. 148 °C [3]). IRν_{max} (KBr): 3075, 2924 (C-H), 1707 (C=O), 1517, 1449 (C=C), 1585.38 (C=C), 1314 (CH_3), 1152 (CN) cm^{-1}. ^{1}H NMR (400 MHz, $CDCl_3$) δ 2.37 (s, 3 H, CH_3), 6.81 (s, 2 H, HC=CH), 7.16 - 7.32 (m, 4 H, 4 x ArH). ^{13}C NMR (101 MHz, $CDCl_3$) ppm 21.1 (CH_3), 126.0 (CH), 128.5, 129.8 (CH), 134.1 (HC=CH), 138.0, 169.7 (O=C).

HRMS (APCI) calculated for $C_{11}H_{10}NO_2$ [M$^+$+H] 188.0712: found 188.0713.

1-(4-(Dimethylamino)phenyl)-1H-pyrrole-2,5-dione (11e)

Compound **11e** was prepared from maleic anhydride (20 mmol) and N,N-dimethylbenzene-1,4-diamine (20 mmol) according to general procedure 1. The product was obtained as red crystals, 1.52 g (35%), Mp. 159-162 °C (lit. M.p. 153-154 °C [4]). IR ν_{max} (KBr): 3066, 2984 (C-H), 1693 (C=O), 1520,

1447.01 (C=C), 1568 (C=C), 1153 (CN) cm^{-1}. ^{1}H NMR (400 MHz, $CDCl_3$) δ 2.98 (s, 6 H, 2 x CH_3), 6.77 (d, *J* = 9.16 Hz, 2 H, 2 x ArH), 6.80 (s, 2 H, 2 x =CH), 7.14 (d, *J* = 8.55 Hz, 2 H, 2 x ArH). ^{13}C NMR (101 MHz, $CDCl_3$) ppm 40.4 (2 x CH_3), 112.4 (CH), 119.4, 127.2 (CH), 134.0 (CH), 150.1, 170.2 (C=O). HRMS (APCI) calculated for $C_{12}H_{13}N_2O_2$ [M^++H] 217.0977: found 217.0975.

1-(4-Benzoylphenyl)-1H-pyrrole-2,5-dione (11f)

Compound **11f** was prepared from maleic anhydride (20 mmol) and (4-aminophenyl)(phenyl)methanone (20 mmol) according to general procedure 1. The product was obtained as a yellow solid, 1.61 g (29%), Mp. 149-159 °C (lit. M.p. 162-164 °C [5]). IR$_{Vmax}$ (KBr): 3093, 2988 (C-H), 1711 (C=O), 1592, 1510 (C=C), 1148 (CN) cm^{-1}. ^{1}H NMR (400 MHz, DMSO-d_6) δ 7.23 (s, 2 H, 2 x =CH), 7.55 - 7.59 (m, 4 H, 4 x ArH), 7.67 (d, *J* = 7.32 Hz, 1 H, 1 x ArH), 7.76 (d, *J* = 7.32 Hz, 2 H, 2 x ArH), 7.85 (d, *J* = 8.55 Hz, 2 H, 2 x ArH). ^{13}C NMR (101 MHz, DMSO-d_6) ppm 126.1 (CH), 128.6 (CH), 129.7 (CH), 129.7 (CH), 130.3 (CH), 132.8 (CH), 134.9 (CH), 135.3, 135.7, 136.8, 169.6 (2 x NC=O), 195.0 (C=O). HRMS (APCI) calculated for $C_{17}H_{12}NO_3$ [M^++H] 278.0817: found 278.0817.

2-(2,5-Dioxo-2,5-dihydro-1H-pyrrol-1-yl)phenyl acetate (11g)

Compound **11g** was prepared from maleic anhydride (20 mmol) and 2-aminophenol (20 mmol) according to general procedure 1. The product was obtained as colourless solid 0.84 g (18%), Mp. 70-75 °C [6]. IR$_{Vmax}$ (KBr): 3098, 2988 (C-H), 1707 (C=O), 1504, 1456 (C=C), 1586.53 (C=C), 1156 (CN) cm^{-1}. ^{1}H NMR (400 MHz, $CDCl_3$) δ 2.15 (s, 3 H, CH_3), 6.82 (s, 2 H, 2 x =CH), 7.19 - 7.34 (m, 3 H, 3 x ArH), 7.35 - 7.47 (m, 1 H, 1 x H3). ^{13}C NMR (101 MHz, $CDCl_3$) ppm 20.7 (CH_3), 123.0, 123.8 (CH), 126.2 (CH), 129.2 (CH), 129.7 (CH), 134.4 (CH), 146.1, 167.9 (C=O), 168.6 (2 x NC=O). HRMS (APCI) calculated for $C_{12}H_{10}NO_4$ [M^++H] 232.0610: found 232.0616.

1-(3-Chlorophenyl)-1H-pyrrole-2,5-dione (11h)

Compound **11h** was prepared from maleic anhydride (20 mmol) and 3-chloroaniline (20 mmol) according to general procedure 1. The product was obtained as a colourless solid, 2.28 g (55%), Mp. 96-102 °C (lit. M.p. 87-89.5 °C [7]). IR$_{Vmax}$ (KBr): 3107, 3083 (C-H), 1712 (C=O), 1577, 1542 (C=C), 1591 (C=C), 1143 (CN) cm^{-1}. ^{1}H NMR (400 MHz, $CDCl_3$) δ 6.80 (s, 2 H, 2 x =CH), 7.21 - 7.26 (m, 1 H, H2), 7.27 - 7.38 (m, 3 H, 3 x ArH). ^{13}C NMR (101 MHz, $CDCl_3$) ppm 123.8 (CH), 125.9 (CH), 127.8 (CH), 129.9 (CH), 132.3, 134.1 (CH), 134.4, 168.8 (C=O). HRMS (APCI) calculated for $C_{10}H_6{}^{35}ClNO_2$ [M^+-e^-] 207.0087; found 207.0097.

3-(2,5-Dioxo-2,5-dihydro-1H-pyrrol-1-yl)phenyl acetate (11i)

Compound **11i** was prepared from maleic anhydride (20 mmol) and 3-aminophenol (20 mmol) according to general procedure 1. The product was obtained as a yellow oil, 1.99 g (43% [6]). IR$_{Vmax}$ (KBr): 3103, 2988, (C-H), 1709 (C=O), 1489, 1449 (C=C), 1595 (C=C), 1369 (CH_3), 1191 (CN) cm^{-1}. ^{1}H NMR (400 MHz, $CDCl_3$) δ 2.27 (s, 3 H, CH_3), 6.81 (s, 2 H, 2 x =CH), 7.06 - 7.13 (m, 1 H, 1 x ArH), 7.17 (t, *J* = 2.14 Hz, 1 H, 1 x ArH), 7.23 - 7.29 (m, 1 H, 1 x ArH), 7.37 - 7.48 (m, 1 H, 1 x ArH). ^{13}C NMR (101 MHz, $CDCl_3$) ppm 21.0 (CH_3), 119.0 (CH), 120.9 (CH), 122.8 (CH), 129.6 (CH), 132.1, 134.2 (CH), 150.8, 169.0 (C=O), 169.1 (2 x N=O). HRMS (APCI) calculated for $C_{12}H_{10}NO_4$ [M^++H] 232.0610: found 232.0617.

1-(3,4-Dimethoxyphenethyl)-1H-pyrrole-2,5-dione (11j)

Compound **11j** was prepared from maleic anhydride (20 mmol) and 2-(3,4-dimethoxyphenyl)ethan-1-amine (20 mmol) according to general procedure 1. The product was obtained as a yellow solid 0.79 g (15%), Mp. 138-144 °C (lit. M.p. 122-124 °C [8]). IR$_{Vmax}$ (KBr): 3093, 2988 (C-H), 1698 (C=O), 1608, 1514.87 (C=C), 1592 (C=C), 1261 (CH_3), 1140 (CN) cm^{-1}. ^{1}H NMR (400 MHz, DMSO-d_6) δ 2.74 (t, *J* = 7.32 Hz, 2 H, CH_2), 3.62 (t, *J* = 7.32 Hz, 2 H, CH_2), 3.71 (s, 3 H, OCH_3), 3.70 (s, 3 H, OCH_3), 6.64 (dd, *J* = 8.24, 1.53 Hz, 1 H, 1 x ArH), 6.74 (d, *J* = 1.83 Hz, 1 H, H2”), 6.82 (d, *J* = 7.94 Hz, 1 H, 1 x ArH), 6.97 (s, 2 H, 2 x =CH). ^{13}C NMR (101 MHz, DMSO-d_6) ppm 33.2 (CH_2), 38.6

(CH_2), 55.4 (OCH_3), 55.4 (OCH_3), 111.7 (CH), 112.3 (CH), 120.5 (CH), 1305, 134.4 (CH), 147.4, 148.6, 170.8 (C=O). HRMS (APCI) calculated for $C_{14}H_{16}NO_4$ [M^++H] 262.1079: found 262.1074.

Methyl 4-(2,5-dioxo-2,5-dihydro-1H-pyrrol-1-yl)benzoate (11k)

Compound **11k** was prepared from maleic anhydride (20 mmol) and methyl 4-aminobenzoate (20 mmol) according to general procedure 1. The product was obtained as a colourless solid 1.85 g (40%), Mp. 161-168 °C (lit. M.p. 161-163 °C [9]). IR$_{Vmax}$ (KBr): 3108, 2958 (C-H), 1705 (C=O), 1601, 1510 (C=C), 1585.52 (C=C), 1140 (CN) cm^{-1}. ^{1}H NMR (400 MHz, $CDCl_3$) δ 3.91 (s, 3 H, OCH_3), 6.86 (s, 2 H, 2 x =CH), 7.48 (m, *J* = 8.55 Hz, 2 H, 2 x ArH), 8.11 (m, *J* = 8.55 Hz, 2 H, 2 x ArH). ^{13}C NMR (101 MHz, $CDCl_3$) ppm 52.2 (OCH_3), 125.1 (CH), 129.0, 130.3 (CH), 134.3 (CH), 135.3, 166.1 (C=O), 168.9 (2 x NC=O). HRMS (APCI) calculated for $C_{12}H_{10}NO_4$ [M^++H] 232.0610: found 232.0610.

1-(3,5-Dimethoxyphenyl)-1H-pyrrole-2,5-dione (11l)

Compound **11l** was prepared from maleic anhydride (20 mmol) and 3,5-dimethoxyaniline (20 mmol) according to general procedure 1. The product was obtained as a yellow solid. 3.21 g (69%), Mp. 161-168 °C (lit. M.p. 121.8 °C [10]). IR$_{Vmax}$ (KBr): 3027, 2970 (C-H), 1717 (C=O), 1602, 1453 (C=C), 1475 (C=C), 1146 (CN) cm^{-1}. ^{1}H NMR (400 MHz, $CDCl_3$) δ 3.79 (s, 6 H, 2 x OCH_3), 6.44 - 6.51 (m, 3 H, 3 x ArH), 6.83 (s, 2 H, 2 x =CH). ^{13}C NMR (101 MHz, $CDCl_3$) ppm 55.4 (OCH_3), 100.3 (CH), 104.6 (CH), 132.6, 134.1 (CH), 160.9, 169.3 (C=O). HRMS (APCI) calculated for $C_{12}H_{11}NO_4$ [M^+] 233.0688: found 233.0692.

1-(4-Fluorophenyl)-1H-pyrrole-2,5-dione (11m)

Compound **11m** was prepared from maleic anhydride (20 mmol) and 4-fluoroaniline (20 mmol) according to general procedure 1. The product was obtained as yellow crystals, 2.48 g (65%), Mp. 160-162 °C (lit. M.p. 136-138 °C [11]). IR$_{Vmax}$ (KBr): 3073, 2988 (C-H), 1708 (C=O), 1601, 1584 (Ar C=C), 1514 (C=C), 1148 (CN) cm^{-1}. ^{1}H NMR (400 MHz, $CDCl_3$) δ 6.85 (s, 2 H, =CH), 7.16 (t, *J* = 8.55 Hz, 2 H, 2 x ArH), 7.33 (dd, *J* = 9.16, 4.88 Hz, 2 H, 2 x ArH). ^{13}C NMR (101 MHz, $CDCl_3$) ppm 116.0 (CH), 116.2 (CH), 127.8 (CH), 127.9, 134.2 (CH), 160.5, 163.0, 169.3 (C=O). HRMS (APCI) calculated for $C_{10}H_6FNO_2$ [M^+] 191.0383: found 191.0384.

1-(4-Bromophenyl)-1H-pyrrole-2,5-dione (11n)

Compound **11n** was prepared from maleic anhydride (20 mmol) and 4-bromoaniline (20 mmol) according to general procedure 1. The product was obtained as yellow crystals (59%), Mp. 120-125 °C (lit. Mp. 122-123 °C [12]). IR$_{Vmax}$ (KBr): 3089, 2988 (C-H), 1719 (C=O), 1592, 1444 (C=C), 1488 (C=C), 1146 (CN) cm^{-1}. ^{1}H NMR (400 MHz, $CDCl_3$) δ 6.84 (s, 2 H, 2 x =CH), 7.22 - 7.28 (m, 2 H, 2 x ArH), 7.55 - 7.60 (m, 2 H, 2 x ArH). ^{13}C NMR (101 MHz, $CDCl_3$) ppm 121.5, 127.3 (CH), 130.2, 132.2 (CH), 134.2 (CH), 169.0 (C=O). HRMS (APCI) calculated for $C_{10}H_6BrNO_2$ [M^+] 250.9582: found 250.9585.

1-(4-Aminophenyl)-1H-pyrrole-2,5-dione (11p)

Compound **11p** was prepared from maleic anhydride (20 mmol) and benzene-1,4-diamine (20 mmol) according to general procedure 1. The product was obtained as a yellow solid, 2.65 g, (70%), Mp. 120-128 °C (lit. Mp. 153 °C [13]). IR$_{Vmax}$ (ATR): 3355 (N-H), 2970, 2938 (C-H), 1692 (C=O), 1585, 1469 (C=C), 1129 (CN) cm^{-1}. ^{1}H NMR (400 MHz, $CDCl_3$) δ 5.75 (s, 2 H. 2 x =CH), 7.21 (s, 2 H, 2 x ArH), 7.46 (s, 2 H, 2 x ArH). ^{13}C NMR (101 MHz, DMSO-d_6) ppm 127.2 (CH), 130.8, 134.7 (CH), 152.3 169.8 (C=O). HRMS (APCI) calculated for $C_{10}H_9N_2O_2$ [M^++H] 189.0659: found 189.0653.

1-(Hydroxymethyl)-1H-pyrrole-2,5-dione (11q)

Compound **11q** was prepared from maleimide and formaldehyde. A solution of 36% formaldehyde (aqueous) (5 mL) and maleimide (2 g) was refluxed at 100 °C for 1 h. The volume of solvent was reduced and the residue was cooled on ice. The crude product was purified by

recrystallisation from 2-propanol to afford a colourless solid, 2.15 g (84%), Mp. 101-104 °C (lit. Mp. 103.5-104.5 °C [14]). IR$_{Vmax}$ (KBr): 3570 (O-H), 1703 (C=O), 1506 (C=C), 1150 (CN) cm^{-1}. ^{1}H NMR (400 MHz, $CDCl_3$) δ 3.13 (t, *J* = 7.94 Hz, 1 H, OH), 5.09 (d, *J* = 7.94 Hz, 2 H, CH_2), 6.78 (s, 2 H, 2 x =CH). ^{13}C NMR (101 MHz, $CDCl_3$) ppm 61.1 (CH_2), 134.6 (CH), 170.1 (C=O). HRMS (APCI) calculated for $C_5H_6NO_3$ [M$^+$+H] 128.0348: found 128.0348.

1-Methyl-1H-pyrrole-2,5-dione (11r)

Compound **11r** was prepared from maleic anhydride (20 mmol) and methylamine (20 mmol) according to general procedure 1. The product was obtained as yellow crystals,1.34 g (60%), Mp. 60-68 °C (lit. M.p. 67 °C [15]). IR$_{Vmax}$ (ATR): 3173, 3095 (C-H), 1696 (C=O), 1600, 1437 (C=C), 1222 (CN) cm^{-1}. ^{1}H NMR (400 MHz, DMSO-d_6) δ 2.86 (s, 3 H, CH_3), 6.99 (s, 2 H, 2 x =CH). ^{13}C NMR (101 MHz, DMSO-d_6) ppm 20.3, 23.3, 24.5, 35.2 (CH_3), 67.9, 134.6 (HC=CH), 169.7, 171.1, 174.1, 174.1. HRMS (APCI) calculated for $C_5H_6NO_2$ [M$^+$+H] 112.0399: found 112.0395.

General Procedure 2: Preparation of ethanoanthracenes

To a solution of the appropriate anthracene derivative (1 mmol) in toluene or xylene (2 mL) was added the required dienophile e.g. maleic anhydride or appropriate maleimide (1.3 mmol). The mixture was heated, with stirring at 90 °C for 48 h. The reaction was then cooled to RT and the resulting solid was isolated by filtration. The solid product was sequentially washed with toluene (2 mL) and diethyl ether (2 mL) and recrystallized from toluene.

General procedure 3: preparation of Triptycene compounds (20a, 20f, 20g)

(i) Preparation of benzenediazonium-2-carboxylate. Anthranilic acid (5.4 g, 0.04 mmol) and trichloroacetic acid 0.06 g in tetrahydrofuran (60 mL) was placed in a 250 mL conical flask. The reaction mixture was stirred and cooled on an ice water bath. Isoamyl nitrite (10 mL) was added portion-wise over 1 min. The mixture is allowed to warm to RT over 1.5 h. The mixture was then cooled again over ice and filtered using a plastic Buchner funnel. Subsequently the tan solid was washed with ice cold tetrahydrofuran until the washings were colourless. The yield of air dried benzenediazonium-2-carboxylate was 78 – 80 %. The benzenediazonium-2-carboxylate was washed with toluene and the tan solid was then stored in solution with toluene (60 mL). (ii) Preparation of Triptycene compounds. To a boiling solution of the appropriate anthracene derivative (4 mmol) in toluene (60 mL) was slowly added a slurry of benzenediazonium-2-carboxylate (prepared from 5.4 g (0.04 mmol) of anthranilic acid) in toluene over the course of 1 h. The mixture was heated to reflux for 1 h. After evaporation of the solvent, the residual oil was subjected to column chromatography (dichloromethane: hexane 1:1) to afford the product which was recrystallised from dichloromethane – hexane gave pure product which was isolated by filtration.

(E)-9-(2-Nitrovinyl)-9,10-dihydro-9,10-[1,2]benzenoanthracene (20a)

Compound **20a** was prepared from (*E*)-9-(2-nitrovinyl)anthracene **12a** (1 g, 4 mmol) and benzenediazonium-2-carboxylate (prepared from anthranilic acid (5.4 g, 0.04 mmol)) according to general procedure 3. The product was obtained as colourless solid, 260 mg (20%), Mp. 258-260 °C (lit. Mp. 262-265 °C [16]). IR$_{Vmax}$ (ATR): 3111, 3069 (C-H), 1651 (C=C), 1457 (C=C), 1526, 1347 (NO_2) cm^{-1}. ^{1}H NMR (400 MHz, $CDCl_3$) δ 5.53 (s, 1 H, C10), 7.05 - 7.16 (m, 6 H, 6 x ArH), 7.44 (d, *J* = 6.71 Hz, 3 H, 3 x ArH), 7.51 (d, *J* = 6.71 Hz, 3 H, 3 x ArH), 7.75 (d, *J* = 14.04 Hz, 1 H, H1′), 8.66 (d, *J* = 14.04 Hz, 1 H, H2′). ^{13}C NMR (101 MHz, $CDCl_3$) ppm 54.1 (C10), 121.4 (CH), 124.2 (CH), 125.2 (CH), 126.0 (CH), 134.6 (C1′), 143.8, 145.2, 146.1 (C2′). HRMS (APCI) calculated for $C_{22}H_{16}NO_2$ [M$^+$+H] 326.1181: found 326.1190.

9,10-[1,2]Benzenoanthracene-9(10H)-carbaldehyde (20f)

Compound **20f** was prepared from 9-anthraldehyde (1.1 g, 5 mmol) and benzenediazonium-2-carboxylate (prepared from anthranilic acid (5.4 g, 0.04 mmol) according to general procedure 3. The

product was obtained as a colourless solid, 296 mg (21%), Mp. 242-244 °C (lit. Mp. 243-257 °C [17]). IR$_{Vmax}$ (ATR): 3020, 2833 (C-H), 1728 (C=O), 1579 (C=C), 1451 (C=C) cm^{-1}. ^{1}H NMR (400 MHz, $CDCl_3$) δ 5.42 (s, 1 H, C10), 7.00 - 7.13 (m, 6 H, 6 x ArH), 7.37 - 7.52 (m, 3 H, 3 x ArH), 7.60 - 7.69 (m, 3 H, 3 x ArH), 11.24 (s, 1 H, CHO). ^{13}C NMR (101 MHz, $CDCl_3$) ppm 54.2 (C10), 60.8 (C9), 122.4 (CH), 124.0 (CH), 125.2 (CH), 125.8 (CH), 142.6, 145.8, 201.0 (CHO). HRMS (APCI) calculated for $C_2H_{14}O$ [M$^+$] 282.1045: found 282.1040.

9,10-[1,2]Benzenoanthracen-9(10H)-ylmethanol (20g)

To a solution of 9,10-[1,2]benzenoanthracene-9(10*H*)-carbaldehyde **20f** (100mg, 0.35 mmol) in dichloromethane (10 mL) and isopropanol (2 mL) was added sodium borohydride (60 mg, 1.6 mmol). The reaction mixture was stirred at room temperature for 24 h and neutralised using 1 M HCl. The solution was extracted with dichloromethane, dried with sodium sulphate and solvent removed *in vacuo*. The product was obtained as a colourless solid, 91 mg (85%), Mp. 240-242 °C (lit. Mp. 242-243 °C [18]). IR$_{Vmax}$ (ATR): 3369 (O-H), 3051, 2981 (C-H), 1712 (C=O), 1609 (C=C), 1455 (C=C) cm^{-1}. ^{1}H NMR (400 MHz, $CDCl_3$) δ 2.21 (br. s., 1 H, OH), 5.26 (br. s., 2 H, CH_2), 5.42 (s, 1 H, C10), 6.98 - 7.10 (m, 6 H, 6 x ArH), 7.42 (d, *J* = 6.71 Hz, 4 H, 4 x ArH), 7.50 (br. s., 2 H, 2 x ArH). ^{13}C NMR (101 MHz, $CDCl_3$) ppm 54.3 (C10), 54.4 (C9), 61.1 (CH_2), 122.0 (CH), 123.6 (CH), 125.1 (CH), 125.2 (CH), 144.4, 146.8. HRMS (ESI) calculated for $C_{21}H_{16}ONa$ [M$^+$+Na] 307.1099: found 307.1097.

9,10-Dihydro-9,10-[3,4]furanoanthracene-12,14-dione (21a)

Compound **21a** was prepared from anthracene (0.18 g, 1 mmol) and maleic anhydride (0.13 g, 1.3 mmol) according to general procedure 2. The product was obtained as a colourless solid, 249 mg (90%), Mp. 264-266 °C (lit. Mp. 267-268 °C [19]). IR$_{Vmax}$ (ATR): 3065 (C-H), 1711 (C=O), 1610 (C=C), 1497, 1455 (C=C) cm^{-1}. ^{1}H NMR (400 MHz, DMSO-d_6) δ 3.66 (s, 2 H, H9 & H10), 4.88 (s, 2 H, H11 & H15), 7.08 - 7.27 (m, 4 H, 4 x ArH), 7.29 - 7.40 (m, 2 H, 2 x ArH), 7.40 - 7.55 (m, 2 H, 2 x ArH). ^{13}C NMR (101 MHz, DMSO-d_6) ppm 44.3 (C9 & C10), 47.9 (C11 & C15), 124.4 (CH), 124.8 (CH), 126.5 (CH), 127.0 (CH), 139.1, 141.1, 171.5 (C12 & C14). HRMS (APCI) calculated for $C_{18}H_{13}O_3$ [M$^+$+H] 277.0865: found 277.0858.

9,10-Dihydro-9,10-[3,4]epipyrroloanthracene-12,14-dione (21b)

Compound **21b** was prepared from anthracene (0.18 g, 1 mmol) and maleimide (0.13 g, 1.3 mmol) according to general procedure 2. The product was obtained as a colourless solid, 234 mg (85%), Mp. 299-300 °C (lit. Mp. 300-301 °C [20]). IR$_{Vmax}$ (ATR): 3347 (O-H), 3065, 2935 (C-H), 1712 (C=O), 1456, 1439 (C=C) cm^{-1}. ^{1}H NMR (400 MHz, DMSO-d_6) δ 3.20 (s, 2 H, H9 & H10), 4.73 (s, 2 H, H11 & H15), 7.08 - 7.21 (m, 4 H, 4 x ArH), 7.21 - 7.32 (m, 2 H, 2 x ArH), 7.37 - 7.51 (m, 2 H, 2 x ArH), 10.77 (br. s., 1 H, NH). ^{13}C NMR (101 MHz, DMSO-d_6) ppm 44.4 (C9 & C10), 47.6 (C11 & C15), 124.1 (CH), 124.7 (CH), 126.1 (CH), 126.4 (CH), 139.5, 142.0, 178.0 (C12 & C14). HRMS (APCI) calculated for $C_{18}H_{14}NO_2$ [M$^+$+H] 276.1025: found 276.1012.

9,10-Dihydro-9,10-[3,4]epipyrroloanthracene-12,14-dione (21c)

Compound **21c** was prepared from anthracene (0.18 g, 1 mmol) and phenylmaleimide (0.224 g, 1.3 mmol) according to general procedure 2. The product was obtained as a colourless solid, 316 mg (90%), Mp. 227-229 °C (lit. Mp. 228-230 °C [21]). IR$_{Vmax}$ (ATR): 3059, 3001 (C-H), 1710 (C=O), 1596 (C=C), 1497, 1445 (C=C) cm^{-1}. ^{1}H NMR (400 MHz, DMSO-d_6) δ 3.41 (s, 2 H, H9 & H10), 4.86 (s, 2 H, H11 & H15), 6.32 - 6.49 (m, 2 H, 2 x ArH), 7.11 - 7.26 (m, 5 H, 5 x ArH), 7.26 - 7.37 (m, 4 H, 4 x ArH), 7.51 (dd, *J* = 5.19, 3.36 Hz, 2 H, 2 x ArH). ^{13}C NMR (101 MHz, DMSO-d_6) ppm 44.8 (C9 & C10), 46.6 (C11 & C15), 124.3, 124.8, 125.3, 126.3, 126.5, 126.6, 128.2, 128.4, 128.8, 128.9, 131.8, 139.3, 141.6, 175.9 (C12 & C14). HRMS (APCI) calculated for $C_{24}H_{18}NO_2$ [M$^+$+H] 352.1338: found 352.1341.

12,14-Dioxo-9,10-[3,4]furanoanthracene-9(10H)-carbaldehyde (21d)

To a solution of 9-anthraldehyde (1.03 g, 5 mmol) in xylene (10 mL) was added maleic anhydride (0.5 g, 5 mmol). The reaction mixture was heated to reflux for 1 h. The reaction was cooled to room temperature, filtered and the precipitate was washed with toluene and hexane. The solid was allow to dry at room temperature and was recrystallized from toluene as a colourless solid, 610 mg (40%), Mp. 235-237 (lit. Mp. 238-239 °C [12]). IR$_{Vmax}$ (ATR): 2980, 2883 (C-H), 1745 (C=O), 1654 (C=C), 1517, 1466 (C=C) cm^{-1}. ^{1}H NMR (400 MHz, DMSO-*d*$_6$) δ 3.80 (dd, *J* = 9.16, 3.05 Hz, 1 H, H10), 4.32 (d, *J* = 9.16 Hz, 1 H, H11), 4.95 (d, *J* = 3.66 Hz, 1 H, H15), 7.10 - 7.34 (m, 5 H, 5 x ArH), 7.38 - 7.48 (m, 1 H, 1 x ArH), 7.58 (dd, *J* = 7.32, 1.22 Hz, 1 H, 1 x ArH), 7.65 - 7.76 (m, 1 H, 1 x ArH), 10.84 (s, 1 H, CHO). ^{13}C NMR (101 MHz, DMSO-*d*$_6$) ppm 44.6 (C10), 48.4 (C11), 48.7 (C15), 57.0 (C9), 122.8 (CH), 123.4 (CH), 125.1 (CH), 125.5 (CH), 126.5 (CH), 127.2 (CH), 127.4 (CH), 127.6 (CH), 136.9, 138.8, 138.8, 141.0, 170.9 (C=O x 2), 201.6 (C=O). HRMS (APCI) calculated for $C_{19}H_{13}O_4$ [M$^+$+H] 305.0814: found 305.0812.

12,14-Dioxo-9,10-[3,4]epipyrroloanthracene-9(10H)-carbaldehyde (21e)

To a solution of 9-anthraldehyde (1.03 g, 5 mmol) in xylene (10 mL) was added maleimide (0.5 g, 5 mmol). The reaction mixture was heated to reflux for 1 h. The reaction was cooled to room temperature, filtered and the precipitate was washed with toluene and hexane. The solid was allow to dry at room temperature and was recrystallized from toluene as a colourless solid, 532 mg (35%), Mp. 280-283 °C (lit. Mp. 286-287 °C [20]). IR$_{Vmax}$ (ATR): 3674 (N-H), 2980, 2892 (C-H), 1732 (C=O), 1583 (C=C), 1468, 1440 (C=C) cm^{-1}. ^{1}H NMR (400 MHz,DMSO-*d*$_6$) δ 3.37 (d, *J* = 3.66 Hz, 1 H, H10), 3.91 (d, *J* = 8.55 Hz, 1 H, H11), 4.79 (d, *J* = 3.66 Hz, 1 H, H15), 7.06 - 7.30 (m, 5 H, 5 x ArH), 7.30 - 7.40 (m, 1 H, 1 x ArH), 7.55 (d, *J* = 7.32 Hz, 1 H, 1 x ArH), 7.62 - 7.83 (m, 1 H, 1 x ArH), 10.78 (s, 1 H, NH), 10.97 (br. s., 1 H, CHO). ^{13}C NMR (101 MHz, DMSO-*d*$_6$) ppm 45.0 (C10), 48.2 (C11), 49.5 (C15), 55.4 (C9), 122.8 (CH), 124.2 (CH), 124.8 (CH), 125.2 (CH), 126.3 (CH), 126.3 (CH), 126.6 (CH), 126.9 (CH), 135.3 (CH), 137.5, 138.9, 140.7, 141.8, 171.1 (C=O), 172.8 (C=O), 177.1 (CHO). HRMS (APCI) calculated for $C_{19}H_{14}NO_3$ [M$^+$+H] 304.0974: found 304.0971.

12,14-Dioxo-13-phenyl-9,10-[3,4]epipyrroloanthracene-9(10H)-carbaldehyde (21f)

To a solution of 9-anthraldehyde (1.03 g, 5 mmol) in xylene (10 mL) was added phenylmaleimide (0.865 g, 5 mmol). The reaction mixture was heated to reflux for 1 h. The reaction was cooled to room temperature, filtered and the precipitate was washed with toluene and hexane. The solid was allow to dry at room temperature and was recrystallized from toluene as a colourless solid, 1.18 g (62%), Mp. 225-226 °C [22]. IR $_{Vmax}$ (ATR): 2970, 2884 (C-H), 1724 (C=O), 1596 (C=C), 1481, 1457 (C=C) cm^{-1}. ^{1}H NMR (400 MHz, DMSO-*d*$_6$) δ 3.57 (dd, *J* = 8.55, 3.66 Hz, 1 H, H10), 4.12 (d, *J* = 8.55 Hz, 1 H, H11), 4.93 (d, *J* = 3.05 Hz, 1 H, H15), 6.35 - 6.48 (m, 2 H, 2 x ArH), 7.13 - 7.43 (m, 9 H, 9 x ArH), 7.62 (d, *J* = 6.71 Hz, 1 H, 1 x ArH), 7.69 - 7.79 (m, 1 H, 1 x ArH), 10.83 (s, 1 H, CHO). ^{13}C NMR (101 MHz, DMSO-*d*$_6$) ppm 45.1 (C10), 47.1 (C11), 47.8 (C15), 57.2 (C9), 122.8 (CH), 123.5 (CH), 125.0 (CH), 125.3 (CH), 126.4 (CH), 126.4 (CH), 126.7 (CH), 127.1 (CH), 127.2 (cH), 128.6 (CH), 128.9 (CH), 131.5 (CH), 137.0, 139.1, 139.2, 141.5, 175.4 (C=O), 175.5 (C=O), 202.0 (CHO). HRMS (APCI) calculated for $C_{25}H_{18}NO_3$ [M$^+$+H] 380.1287: found 380.1282.

12,14-Dioxo-9,10-[3,4]furanoanthracene-9(10H)-carboxylic acid (21g)

To a solution of anthracene-9-carboxylic acid (1.1 g, 5 mmol) in xylene (10 mL) was added maleic anhydride (0.5 g, 5 mmol). The reaction mixture was heated to reflux for 1 h. The reaction was cooled to room temperature, filtered and the precipitate was washed with toluene and hexane. The solid was allow to dry at room temperature and was recrystallized from toluene as a colourless solid, 1.30 g (81%), Mp. 265-267 °C (lit. M.p. 268 °C [23]. IR$_{Vmax}$ (ATR): 2980, 2884 (C-H), 1746 (C=O), 1656 (C=C), 1583, 1469 (C=C) cm^{-1}. ^{1}H NMR (400 MHz, DMSO-*d*$_6$) δ 3.70 (dd, *J* = 9.16, 3.05 Hz, 1 H, H10), 4.16 (d, *J* = 9.77 Hz, 1 H, H11), 4.91 (d, *J* = 3.05 Hz, 1 H, H15), 7.15 - 7.32 (m, 5 H, 5 x ArH), 7.34 - 7.40 (m, 1 H, 1 x ArH), 7.52 - 7.59 (m, 1 H, 1 x ArH), 7.88 - 7.98 (m, 1 H, 1 x ArH), 14.14 (br. s., 1 H, COOH). ^{13}C NMR (101 MHz, DMSO-*d*$_6$) ppm 44.8 (C10), 48.4 (C11), 50.1 (C15), 55.8 (C9), 122.8 (CH), 124.6 (CH), 125.0

(CH), 125.0 (CH), 126.8 (CH), 127.0 (CH), 127.3 (CH), 137.2, 138.4, 139.7, 140.7, 170.3 (C=O), 170.3 (C=O), 171.1 (C=O). HRMS (APCI) calculated for $C_{19}H_{13}O_5$ [M⁺+H] 321.0763: found 321.0760.

12,14-Dioxo-9,10-[3,4]epipyrroloanthracene-9(10H)-carboxylic acid (21h)

To a solution of anthracene-9-carboxylic acid (1.1 g, 5 mmol) in xylene (10 mL) was added maleimide (0.5 g, 5 mmol). The reaction mixture was heated to reflux for 1 h. The reaction was cooled to room temperature, filtered and the precipitate was washed with toluene and hexane. The solid was allow to dry at room temperature and was recrystallized from toluene as a colourless solid, 656 mg (41%), Mp. 298-299 °C [24]. IR$_{Vmax}$ (ATR): 3327 (N-H), 2970, 2931 (C-H), 1719 (C=O), 1583 (C=C), 1482, 1459 (C=C) cm^{-1}. ^{1}H NMR (400 MHz, DMSO-*d*$_6$) δ 3.23 (dd, *J* = 8.55, 3.05 Hz, 1 H, H10), 3.78 (d, *J* = 8.55 Hz, 1 H, H11), 4.71 (d, *J* = 3.05 Hz, 1 H, H15), 7.04 - 7.30 (m, 6 H, 6 x ArH), 7.31 - 7.60 (m, 1 H, 1 x ArH), 7.87 - 8.06 (m, 1 H, 1 x ArH), 10.78 (s, 1 H, NH), 13.69 (br. s., 1 H, COOH). ^{13}C NMR (101 MHz, DMSO-*d*$_6$) ppm 45.0 (C10), 48.2 (C11), 49.5 (C5), 55.4 (C9), 122.8 (CH), 124.2 (CH), 124.8 (CH), 125.2 (CH), 126.3 (CH), 126.3 (CH), 126.6 (CH), 126.9 (CH), 135.3 (CH), 137.5, 139.0, 140.7, 141.7, 171.1 (C=O), 172.7 (C=O), 177.1 (COOH). HRMS (APCI) calculated for $C_{19}H_{14}NO_4$ [M⁺+H] 320.0923: found 320.0921.

12,14-Dioxo-13-phenyl-9,10-[3,4]epipyrroloanthracene-9(10H)-carboxylic acid (21i)

To a solution of anthracene-9-carboxylic acid 1.1 g (5 mmol) in 10 mL of xylene was added phenyl maleimide 0.865 g (5 mmol). The reaction mixture was heated to reflux for 1 hour. The reaction was cooled to room temperature, filtered and the precipitate was washed with toluene and hexane. The solid was allow to dry at room temperature and was recrystallized from toluene as a colourless solid, (43%), Mp. 290-292 °C [24]. IR $_{Vmax}$ (ATR): 3292 (O-H), 2980, 2891 (C-H), 1743 (C=O), 1583 (C=C), 1483, 1465 (C=C) cm^{-1}. ^{1}H NMR (400 MHz, DMSO-*d*$_6$) δ ppm 3.48 (dd, *J* = 8.55, 3.05 Hz, 1 H, H10), 4.00 (d, *J* = 8.55 Hz, 1 H, H11), 4.89 (d, *J* = 3.05 Hz, 1 H, H15), 6.44 (dd, *J* = 7.63, 1.53 Hz, 2 H, 2 x ArH), 7.16 - 7.41 (m, 9 H, 9 x ArH), 7.58 (dd, *J* = 5.49, 3.05 Hz, 1 H, 1 x ArH), 7.95 - 8.06 (m, 1 H, 1 x ArH), 13.89 (br. s., 1 H, COOH). ^{13}C NMR (101 MHz, DMSO-*d*$_6$) δ ppm 45.4 (C10), 47.2 (C11), 48.7 (C15), 55.9 (C9), 122.9 (CH), 124.4 (CH), 124.8 (CH), 125.3 (CH), 126.5 (CH), 126.5 (CH), 126.6 (CH), 126.8 (CH), 127.1 (CH), 128.5 (CH), 128.8 (CH), 131.7, 137.4, 138.9, 140.2, 141.3, 171.0 (C=O), 175.0 (C=O), 175.5 (C=O). HRMS (APCI) calculated for $C_{25}H_{18}NO_4$ [M+H] 396.1236: found 396.1230.

9-Hydroxy-9,10-dihydro-9,10-[3,4]furanoanthracene-12,14-dione (21j)

To a solution of anthrone (0.97 g, 5 mmol) in xylene (10 mL) was added maleic anhydride (0.5 g, 5 mmol). The reaction mixture was heated to reflux for 1 h. The reaction was cooled to room temperature, filtered and the precipitate was washed with toluene and hexane. The solid was allow to dry at room temperature and was recrystallized from toluene as a colourless solid, 805 mg (55%), Mp. 215-218 °C (lit. Mp. 218-219 °C [25]). IR$_{Vmax}$ (ATR): 3481 (O-H), 2970, 2883 (C-H), 1767 (C=O), 1660 (C=C), 1479, 1458 (C=C) cm^{-1}. ^{1}H NMR (400 MHz, DMSO-*d*$_6$) δ 3.50 (d, *J* = 9.16 Hz, 1 H, H11), 3.74 (dd, *J* = 9.16, 3.66 Hz, 1 H, H10), 4.82 (d, *J* = 3.05 Hz, 1 H, H15), 7.14 - 7.29 (m, 4 H, 4 x ArH), 7.33 (d, *J* = 6.71 Hz, 1 H, 1 x ArH), 7.46 (d, *J* = 7.32 Hz, 1 H, 1 x ArH), 7.55 (t, *J* = 6.41 Hz, 2 H, 2 x ArH). ^{13}C NMR (101 MHz, DMSO-*d*$_6$) ppm 43.3 (C11), 49.2 (C10), 52.2 (C15), 76.1 (C9), 120.9 (CH), 121.2 (CH), 123.9 (CH), 124.4 (CH), 126.3 (CH), 126.5 (CH), 126.9 (CH), 126.9 (CH), 137.2, 139.5, 140.9, 144.2, 168.8 (C=O), 171.4 (C=O). HRMS (APCI) calculated for $C_{18}H_{13}O_4$ [M⁺+H] 293.0814: found 293.0814.

2-(Anthracen-9-ylmethylene)malononitrile (22a)

To a solution of 9-anthraldehyde (0.393 g, 1.91 mmol) and malononitrile (0.372 g, 6.11 mmol) in ACN (50 mL), was added 1 drop of piperidine. The solution was then heated at 90 °C for 0.5 h. The solution was then concentrated *in vacuo*, the residual solid dissolved in DCM and washed with HCl, water and brine. The organic phases were combined and dried over Na_2SO_4, solvent removed *in vacuo* and was recrystallized from ethanol as orange crystals, 459 mg, (95%), Mp. 256-257 °C (lit. Mp. 206 °C [26]). IR$_{Vmax}$ (KBr): 2965, 2932 (C-H), 2228 (CN), 1621 (C=C), 1574, 1445 (Ar C=C) cm^{-1}. ^{1}H NMR (400 MHz, $CDCl_3$) δ 7.55 (t, *J* = 7.63 Hz, 3 H, 3 xArH), 7.65 (t, *J* = 7.63 Hz, 2 H, 2 x ArH), 7.89 (d, *J* = 8.54 Hz,

2 H, H4 & H5), 8.05 (d, *J* = 7.93 Hz, 2 H, H8 & H1), 8.59 (s, 1 H, H10), 8.88 (s, 1 H, H1′). ^{13}C NMR (101 MHz, $CDCl_3$) ppm 92.2, 108.9, 111.5 (CN), 113.0 (CN), 123.4 (C9), 123.8, 126.1, 128.3, 129.1, 129.5, 130.8, 132.5, 160.8 (C2′). HRMS (ESI) calculated for $C_{18}H_9N_2$ [M$^+$-H] 253.0771: found 253.0764.

(E)-3-(Anthracen-9-yl)acrylonitrile (22b)

To a solution of 9-anthraldehyde (1 g, 4.85 mmol) and cyanoacetic acid (0.52 g, 6.11 mmol) in DMF (6 mL), was added morpholine (0.7 mL). The solution was then heated at 90 °C for 6 h. The solution was then cooled to -10 °C for 16 h. The resultant crystals were filtered and washed with diethyl ether, to afford the product as orange crystals, 446 mg (40%), Mp. 205-207 °C (lit. Mp. 209.5-210.5 °C [27]). IR$_{Vmax}$ (KBr): 3051 (C-H), 2218 (CN), 1623, 1442.02 (C=C) cm^{-1}. ^{1}H NMR (400 MHz, $CDCl_3$) δ 5.84 (d, *J* = 17.09 Hz, 1 H =CH), 7.43 - 7.54 (m, 4 H, 4 x ArH), 7.97 (d, *J* = 7.93 Hz, 2 H, H4 & H5), 8.06 (d, *J* = 8.54 Hz, 2 H, H1 & H8), 8.27 (d, *J* = 17.09 Hz, 1 H, =CH), 8.41 (s, 1 H, H10). ^{13}C NMR (101 MHz, $CDCl_3$) ppm 105.4 (C2′), 117.4 (CN), 124.3 (C9), 125.4 (CH), 126.9 (CH), 127.6, 128.9 (CH), 129.0 (CH), 129.2, 131.0, 148.4 (C1′). HRMS (APCI) calculated for $C_{17}H_{12}N$ [M$^+$+H] 230.0966: found 230.097.

(E)-(Anthracen-9-ylmethylene)hydrazine (22c)

To a solution of 9-anthraldehyde (1 g, 4.85 mmol) in DCM (20 ml) and ethanol (10 mL), was added dropwise hydrazine (1 g, 20 mmol). The solution was then stirred at RT for 24 h. The solvent was removed *in vacuo*. The residual solid was recrystallized from a mixture of dichloromethane and hexane. The product was isolated as an orange solid, 0.965 g (90%), Mp. 122-124 °C (lit. Mp. 125-126 °C [28]). IR$_{Vmax}$ (ATR): 3363 (N-H), 3044, 2982 (C-H), 1622 (C=C), 1484, 1440 (C=C) cm^{-1}. ^{1}H NMR (400 MHz, DMSO-d_6) δ 7.18 (s, 2 H, NH_2), 7.52 (br. s., 4 H, 4 x ArH), 8.07 (d, *J* = 7.32 Hz, 2 H, 2 x ArH), 8.52 (s, 1 H, C10), 8.62 (d, *J* = 8.55 Hz, 2 H, 2 x ArH), 8.89 (s, 1 H, C1′). ^{13}C NMR (101 MHz, DMSO-d_6) ppm 125.3 (CH), 125.4 (CH), 125.9 (CH), 126.8 (CH), 127.8, 128.6 (CH), 129.0, 131.1, 136.5 (C1′). HRMS (APCI) calculated for $C_{15}H_{13}N_2$ [M$^+$+H] 221.1079: found 221.1080.

(E)-Anthracene-9-carbaldehyde oxime (22e)

9-Anthraldehyde (1 g) was dissolved in ethanol (20 mL) and heated to 75 °C. To this was added hydroxylamine hydrochloride 0.4 g in water 3.33 mL (neutralised with sodium carbonate). The mixture was heated for ten minutes and diluted with water until cloudy. The reaction mixture was then iced and the precipitate was filtered to afford the product as a white solid, 0.99 g (90%), Mp. 157-160 °C (lit. Mp. 159-162 °C [29]). IR$_{Vmax}$ (ATR): 3420 (O-H), 3084, 3012, 2980 (C-H), 1634, 1622, 1482 (C=C) cm^{-1}. ^{1}H NMR (400 MHz, DMSO-d_6) δ 7.45 - 7.65 (m, 4 H, 4 x ArH), 8.03 - 8.19 (m, 2 H, 2 x ArH), 8.41 - 8.54 (m, 2 H, 2 x ArH), 8.62 (s, 1 H, H10), 9.23 (s, 1 H, OH). ^{13}C NMR (101 MHz, DMSO-d_6) ppm 124.8, 125.1 (CH), 125.2 (CH), 125.4 (CH), 126.1 (CH), 126.7 (CH), 128.4 (CH), 128.6, 128.7 (CH), 129.5, 130.9, 146.4 (CH), 146.5 (CH). HRMS (APCI) calculated for $C_{15}H_{12}NO$ [M$^+$+H] 222.0919: found 222.0914.

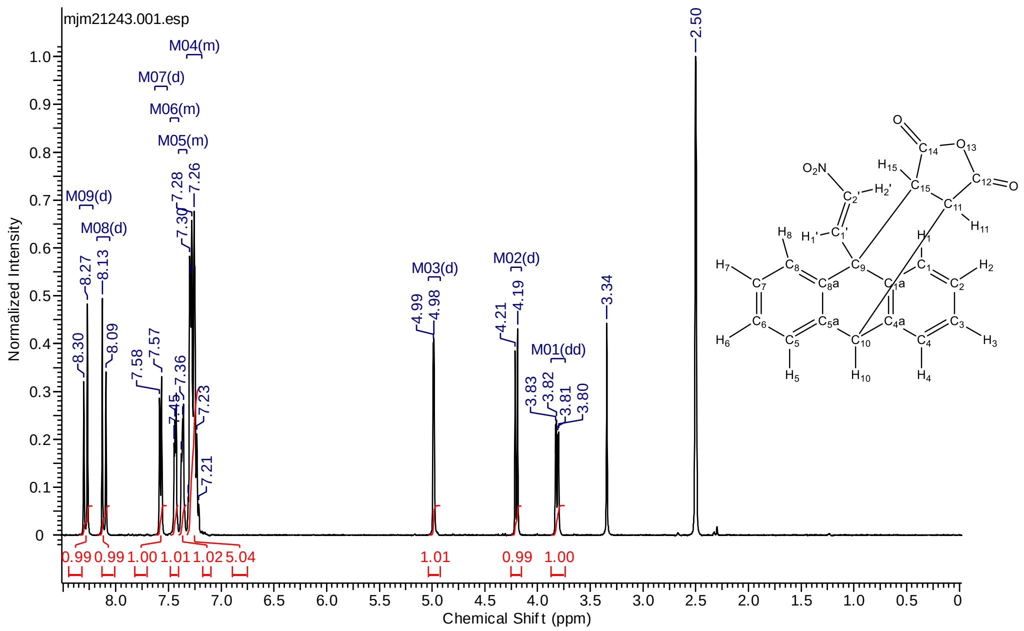

Figure S1. ^{1}H NMR spectrum of (*E*)-9-(2-nitrovinyl)-9,10-dihydro-9,10-[3,4]furanoanthracene-12,14-dione (13a).

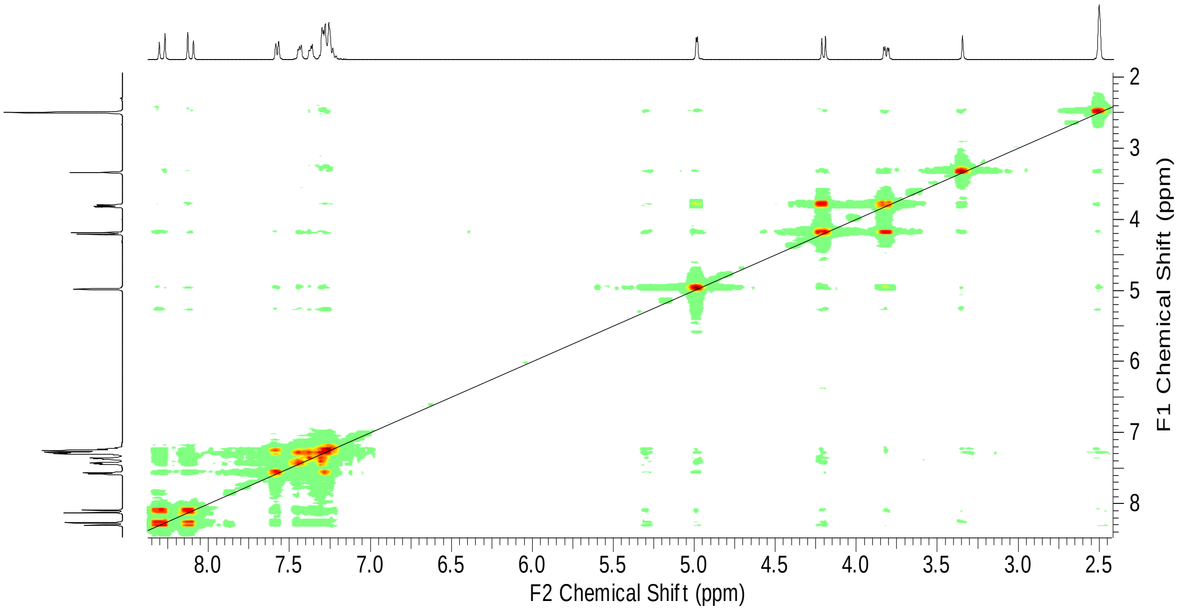

Figure S2. H-H COSY NMR spectrum of (*E*)-9-(2-nitrovinyl)-9,10-dihydro-9,10-[3,4]furanoanthracene-12,14-dione (13a).

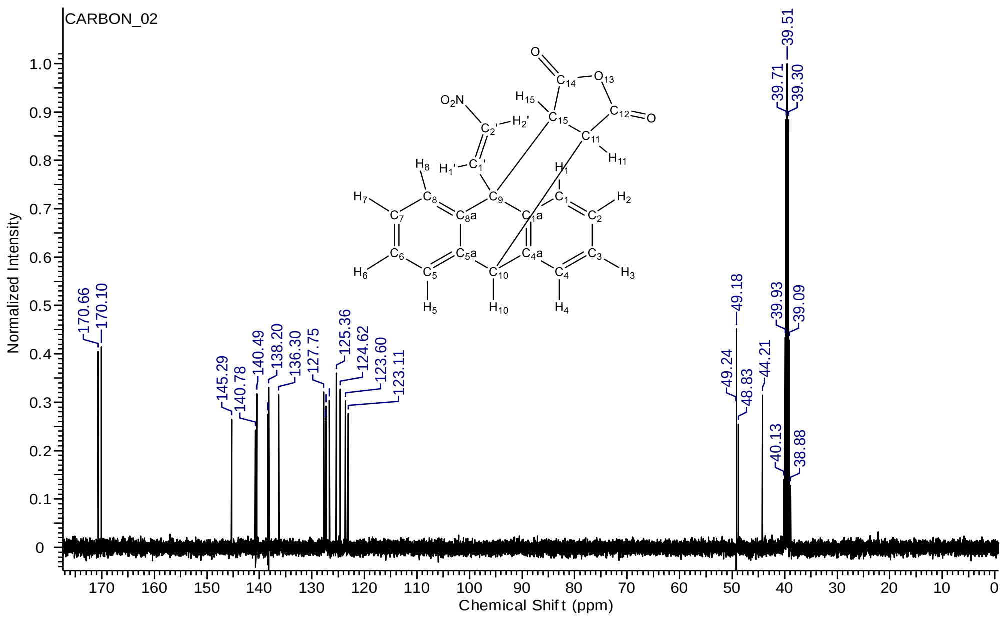

Figure S3. ^{13}C NMR spectrum of (*E*)-9-(2-nitrovinyl)-9,10-dihydro-9,10-[3,4]furanoanthracene-12,14-dione (13a).

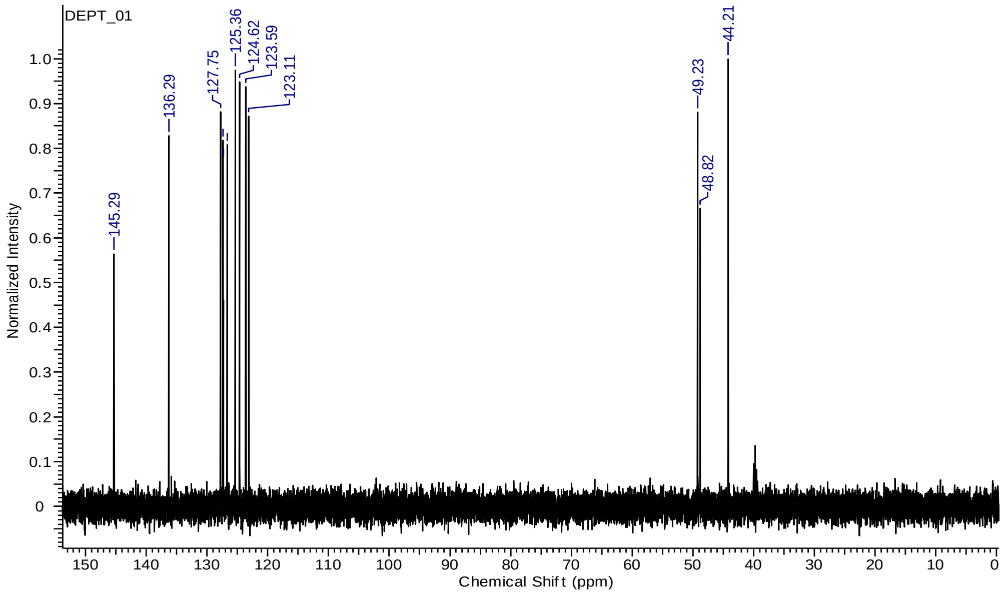

Figure S4. DEPT 90 NMR spectrum of (*E*)-9-(2-nitrovinyl)-9,10-dihydro-9,10-[3,4]furanoanthracene-12,14-dione (13a).

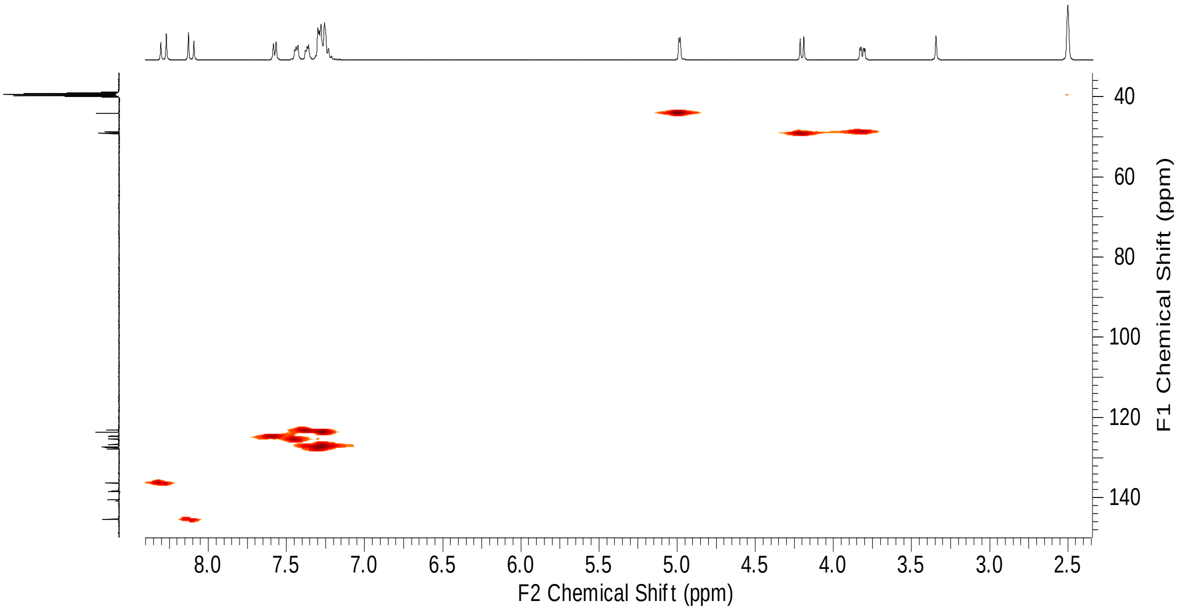

Figure S5. C-H COSY NMR spectrum of (*E*)-9-(2-nitrovinyl)-9,10-dihydro-9,10-[3,4]furanoanthracene-12,14-dione (13a).

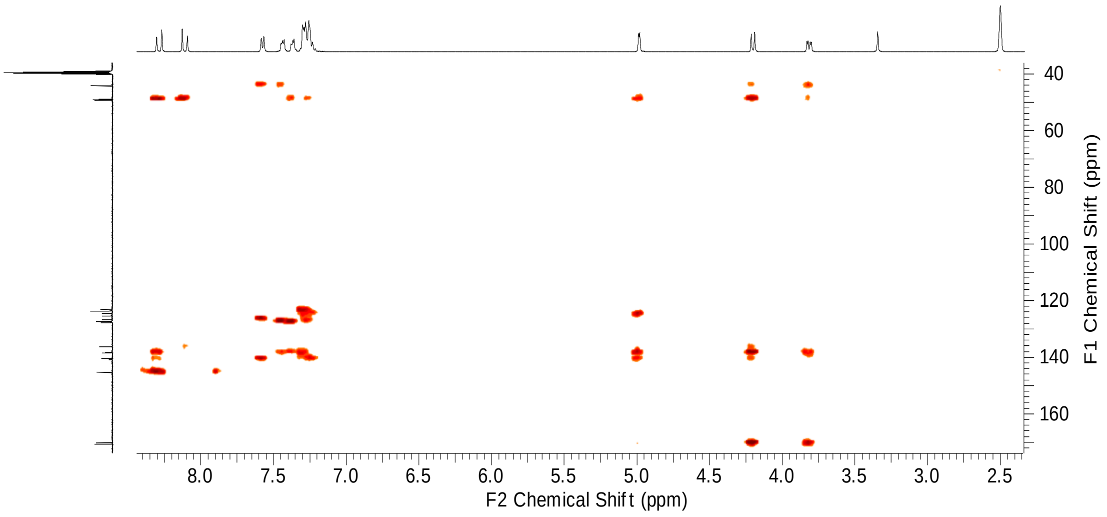

Figure S6. HMBC NMR spectrum of (*E*)-9-(2-nitrovinyl)-9,10-dihydro-9,10-[3,4]furanoanthracene-12,14-dione (13a).

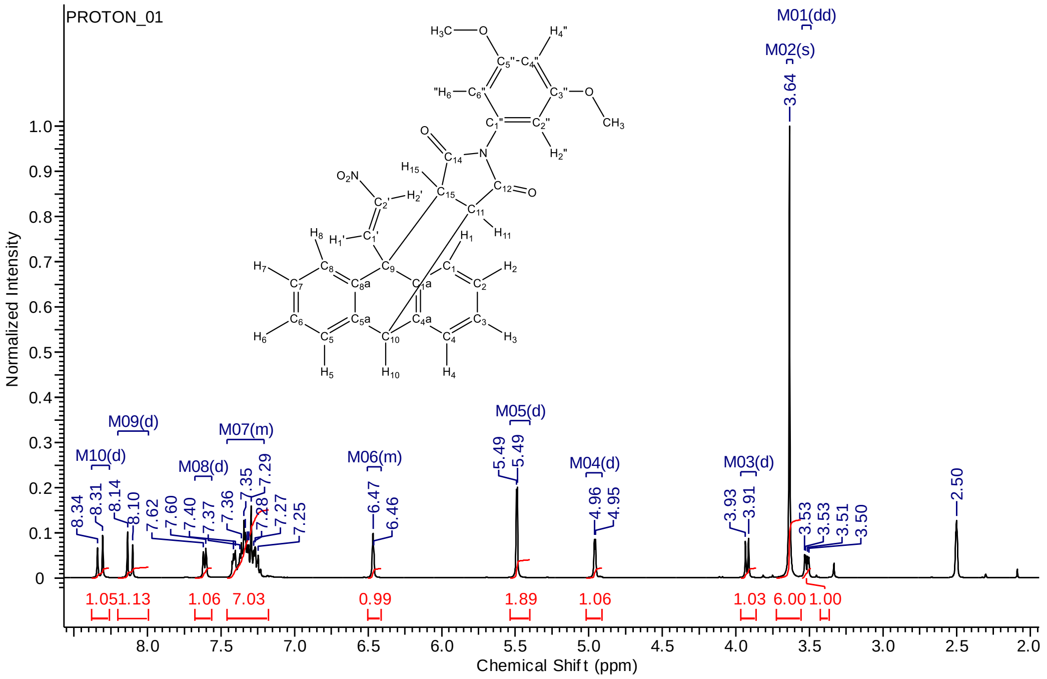

Figure S7. ^{1}H NMR spectrum of (*E*)-13-(3,5-dimethoxyphenyl)-9-(2-nitrovinyl)-9,10-dihydro-9,10-[3,4]epipyrroloanthracene-12,14-dione (16j).

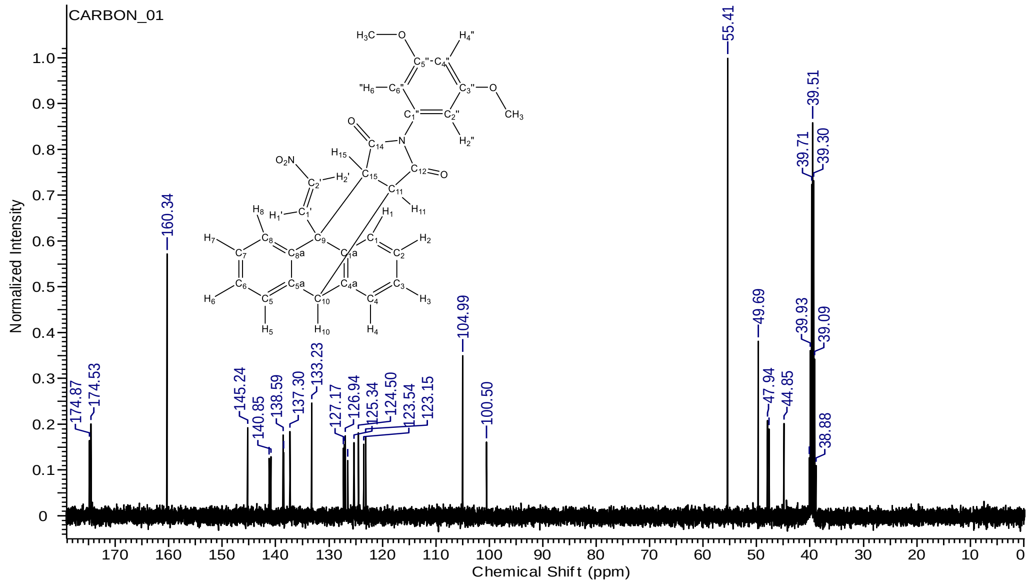

Figure S8. ^{13}C NMR spectrum of (*E*)-13-(3,5-dimethoxyphenyl)-9-(2-nitrovinyl)-9,10-dihydro-9,10-[3,4]epipyrroloanthracene-12,14-dione (16j).

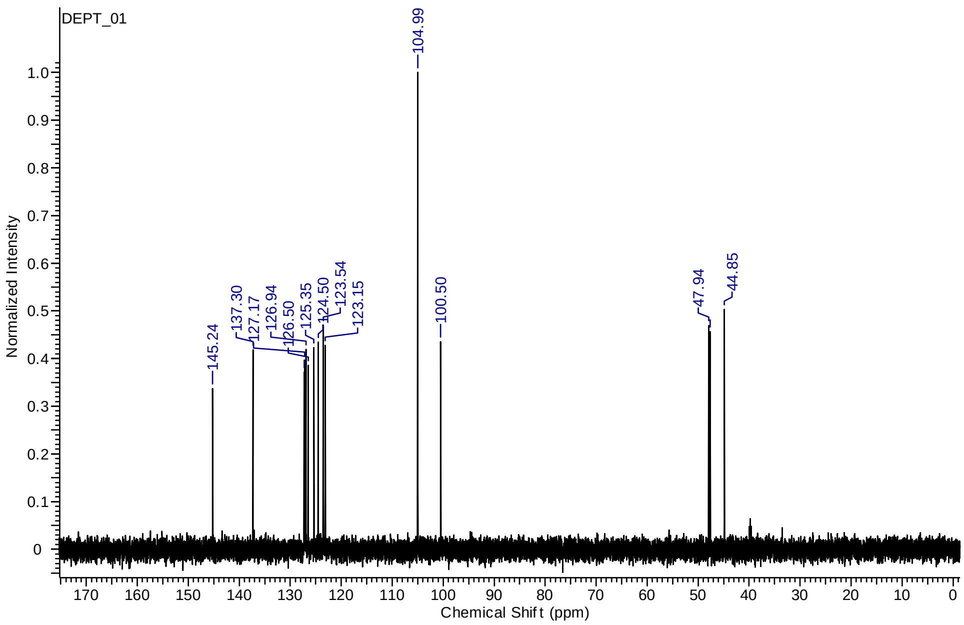

Figure S9. DEPT 90 NMR spectrum of (*E*)-13-(3,5-dimethoxyphenyl)-9-(2-nitrovinyl)-9,10-dihydro-9,10-[3,4]epipyrroloanthracene-12,14-dione (16j).

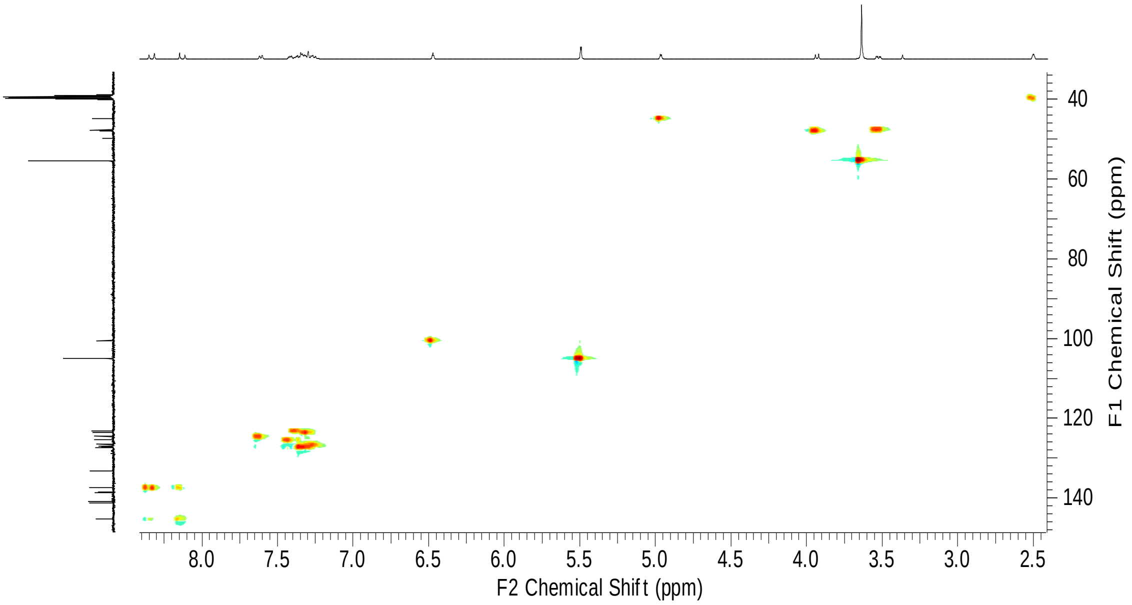

Figure S10. C-H COSY spectrum of (*E*)-13-(3,5-dimethoxyphenyl)-9-(2-nitrovinyl)-9,10-dihydro-9,10-[3,4]epipyrroloanthracene-12,14-dione (16j).

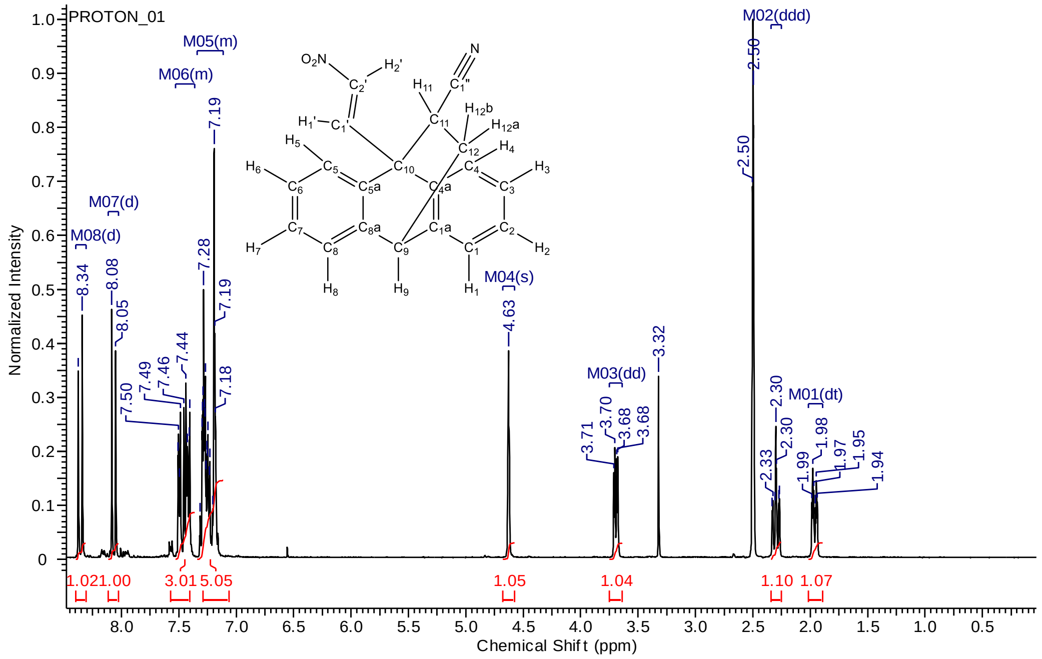

Figure S11. ^{1}H NMR spectrum of (*E*)-10-(2-nitrovinyl)-9,10-dihydro-9,10-ethanoanthracene-11-carbonitrile (19a).

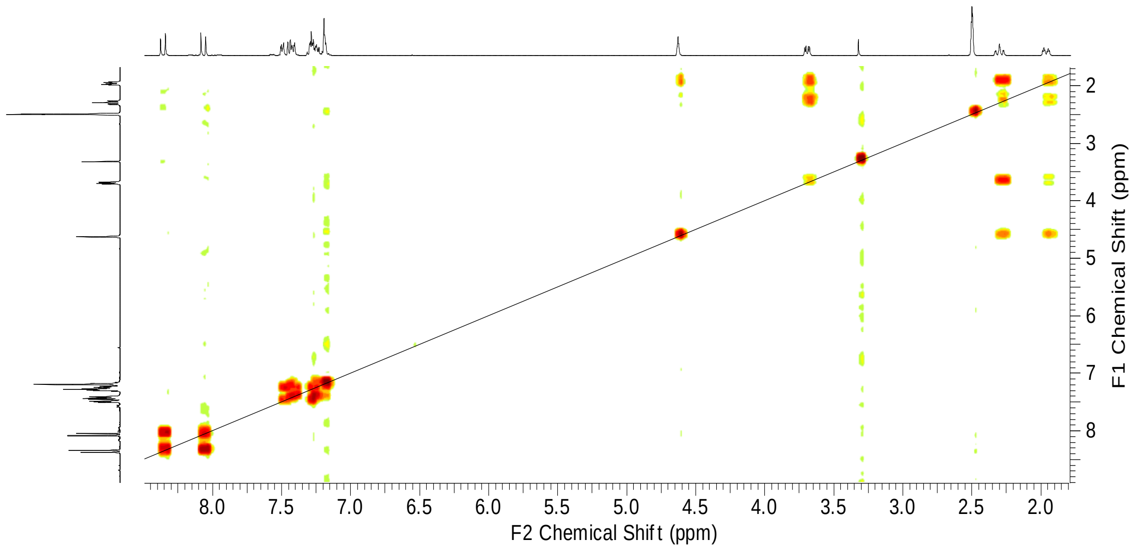

Figure S12. H-H COSY spectrum of (*E*)-10-(2-nitrovinyl)-9,10-dihydro-9,10-ethanoanthracene-11-carbonitrile (19a).

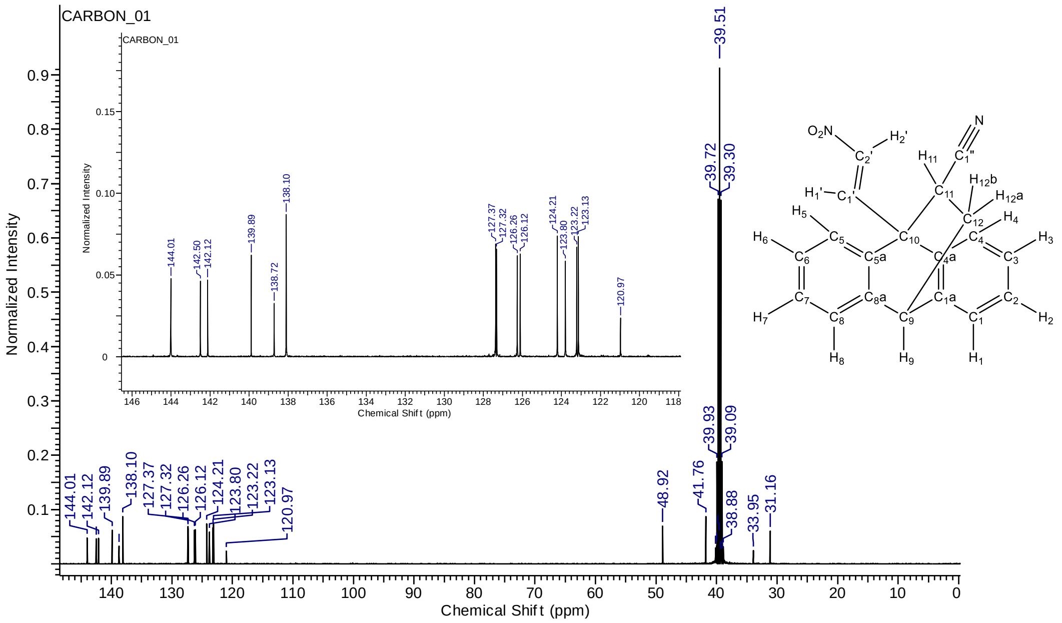

Figure S13. ^{13}C NMR spectrum of (*E*)-10-(2-nitrovinyl)-9,10-dihydro-9,10-ethanoanthracene-11-carbonitrile (19a).

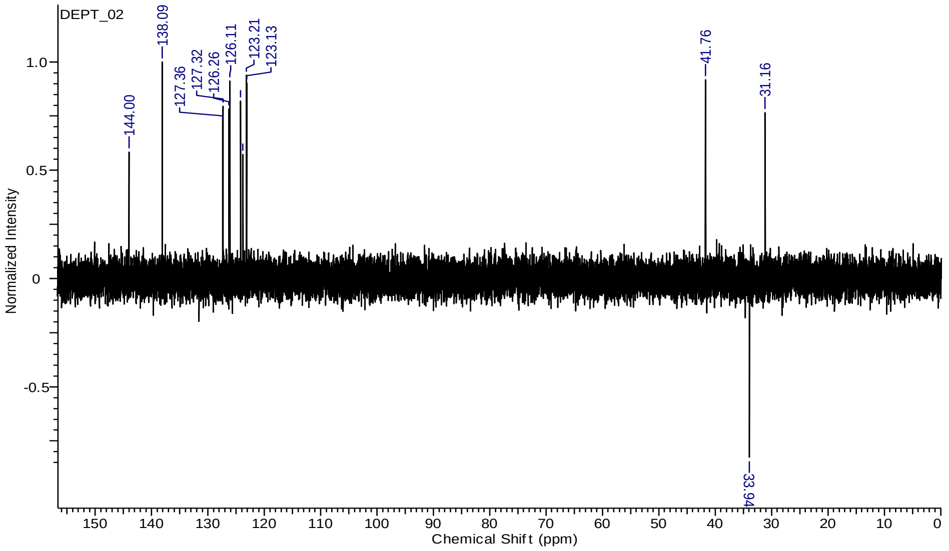

Figure S14. DEPT 135 spectrum of (*E*)-10-(2-nitrovinyl)-9,10-dihydro-9,10-ethanoanthracene-11-carbonitrile (19a).

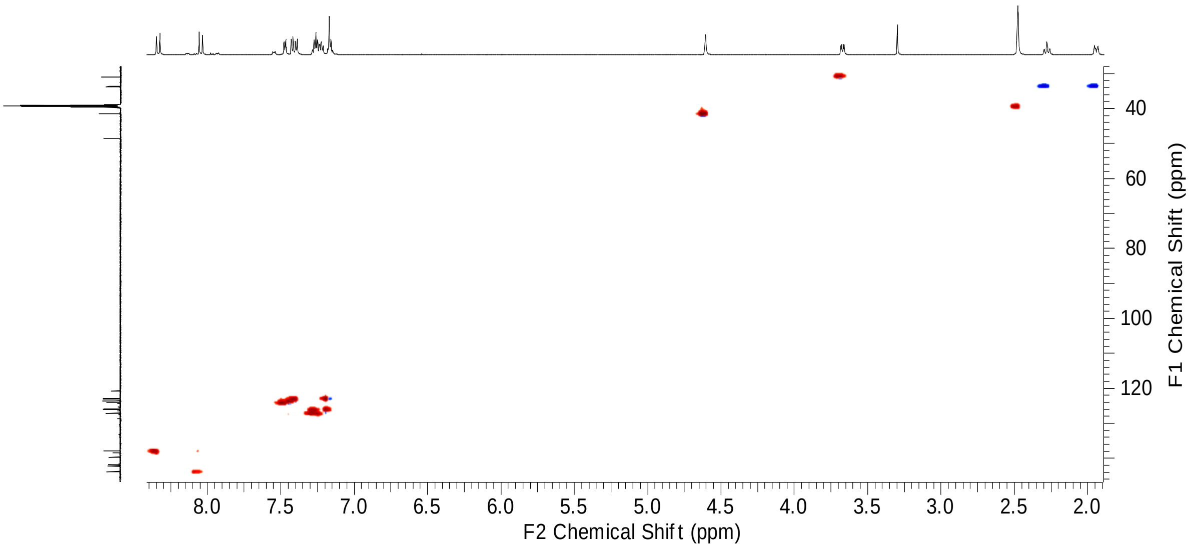

Figure S15. C-H COSY spectrum of (*E*)-10-(2-nitrovinyl)-9,10-dihydro-9,10-ethanoanthracene-11-carbonitrile (19a).

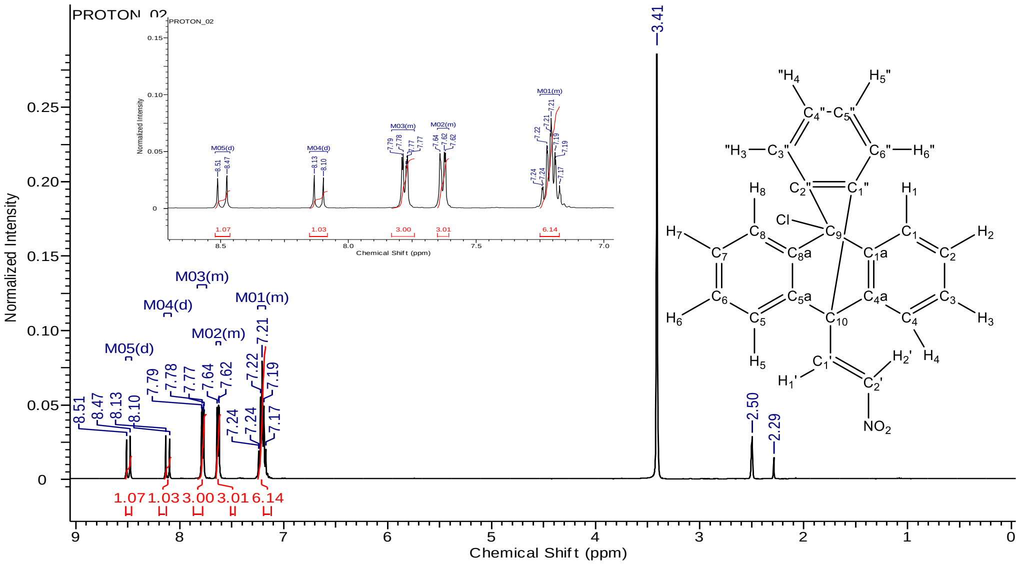

Figure S16. ^{1}H NMR spectrum of (*E*)-9-chloro-10-(2-nitrovinyl)-9,10-dihydro-9,10-[1,2]benzenoanthracene (20d).

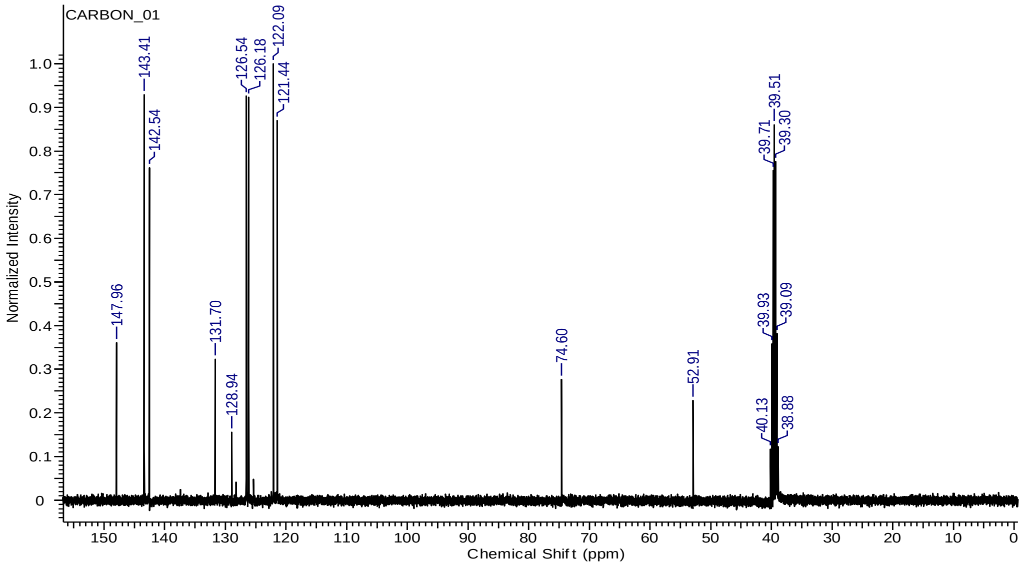

Figure S17. ^{13}C NMR spectrum of (*E*)-9-chloro-10-(2-nitrovinyl)-9,10-dihydro-9,10-[1,2]benzenoanthracene (20d).

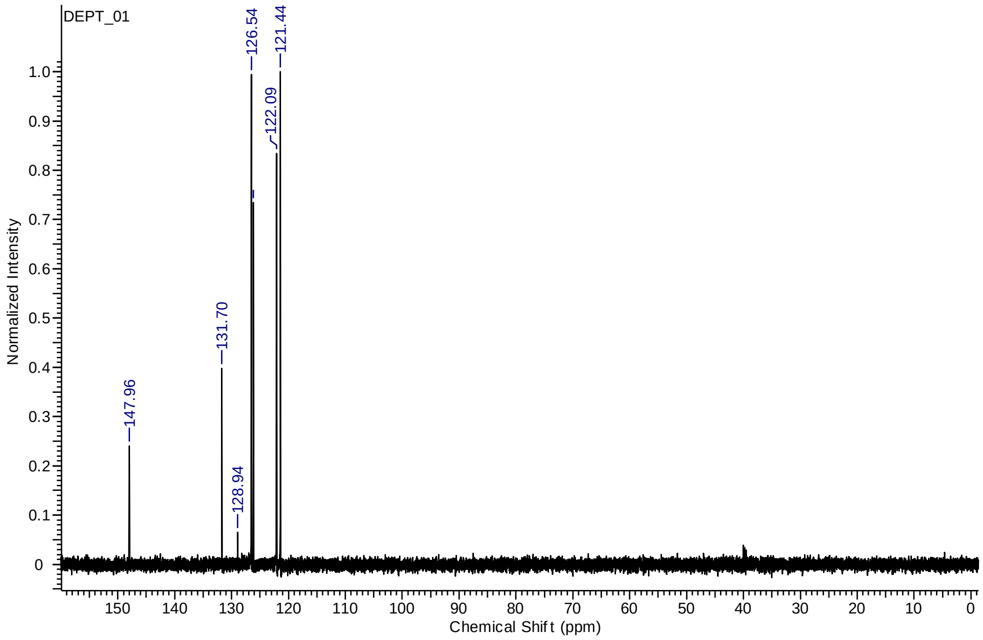

Figure S18. DEPT 90 spectrum of (*E*)-9-chloro-10-(2-nitrovinyl)-9,10-dihydro-9,10-[1,2]benzenoanthracene (20d).

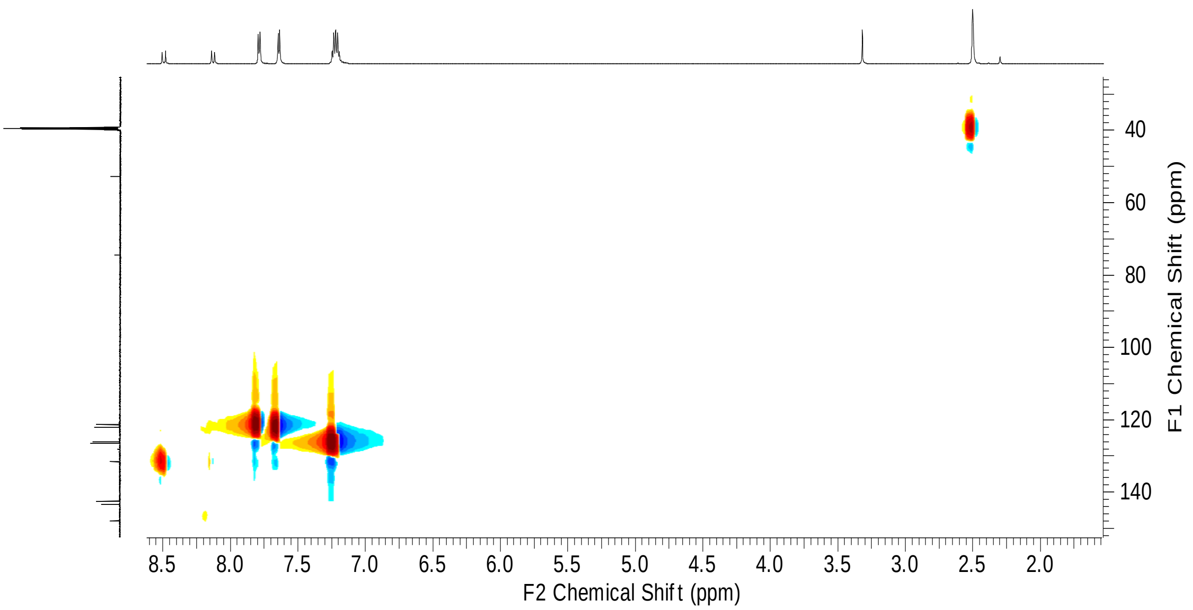

Figure S19. C-H COSY spectrum of (*E*)-9-chloro-10-(2-nitrovinyl)-9,10-dihydro-9,10-[1,2]benzenoanthracene (20d).

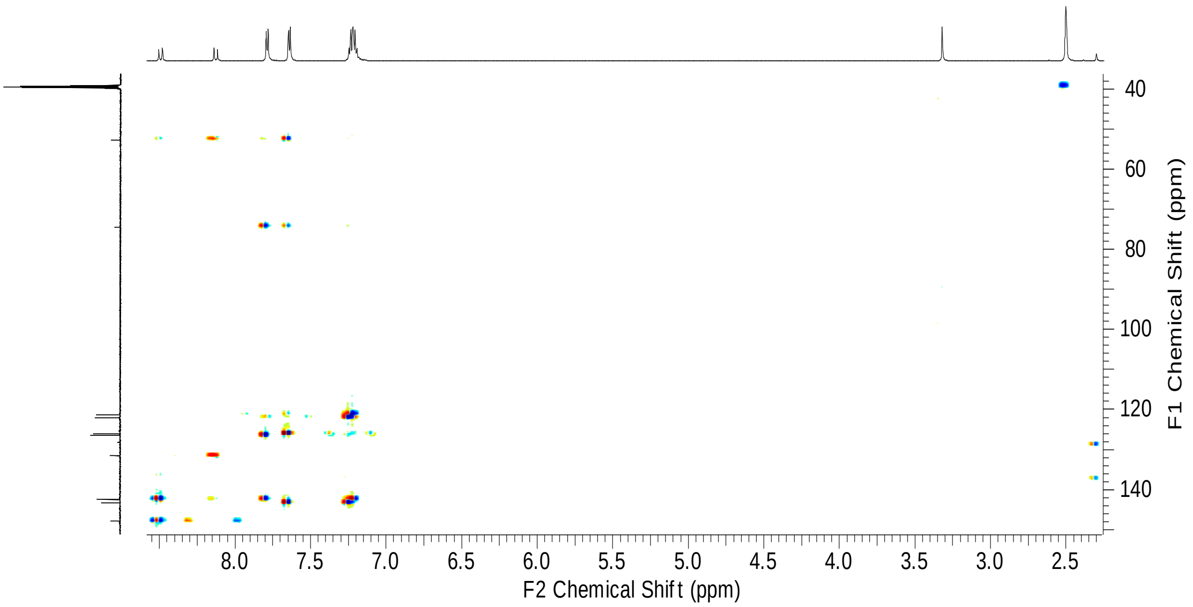

Figure S20. HMBC spectrum of (*E*)-9-chloro-10-(2-nitrovinyl)-9,10-dihydro-9,10-[1,2]benzenoanthracene (20d).

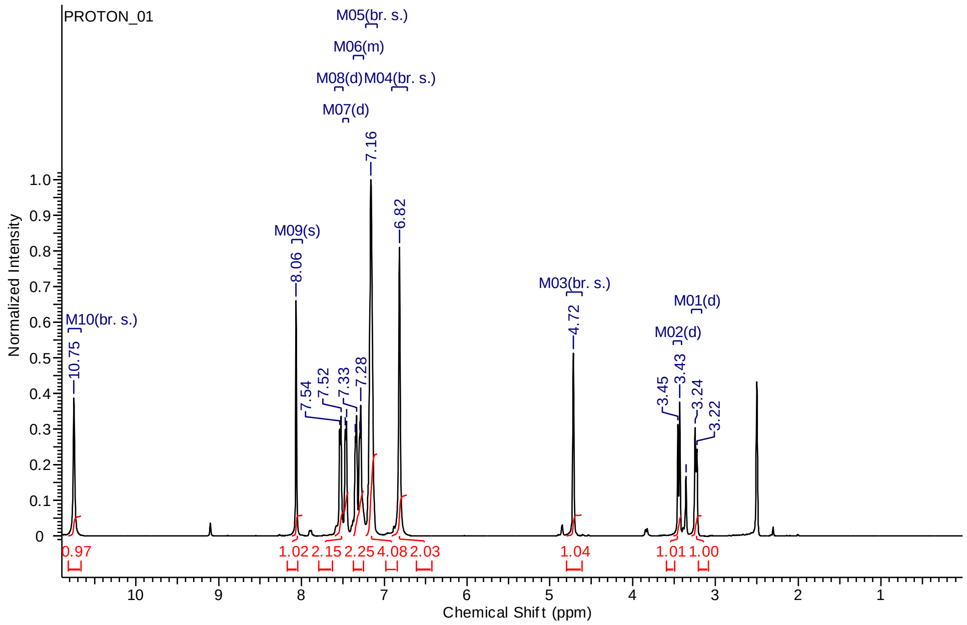

Figure S21. ^{1}H NMR spectrum of (*E*)-9-(hydrazonomethyl)-9,10-dihydro-9,10-[3,4]epipyrroloanthracene-12,14-dione (23g).

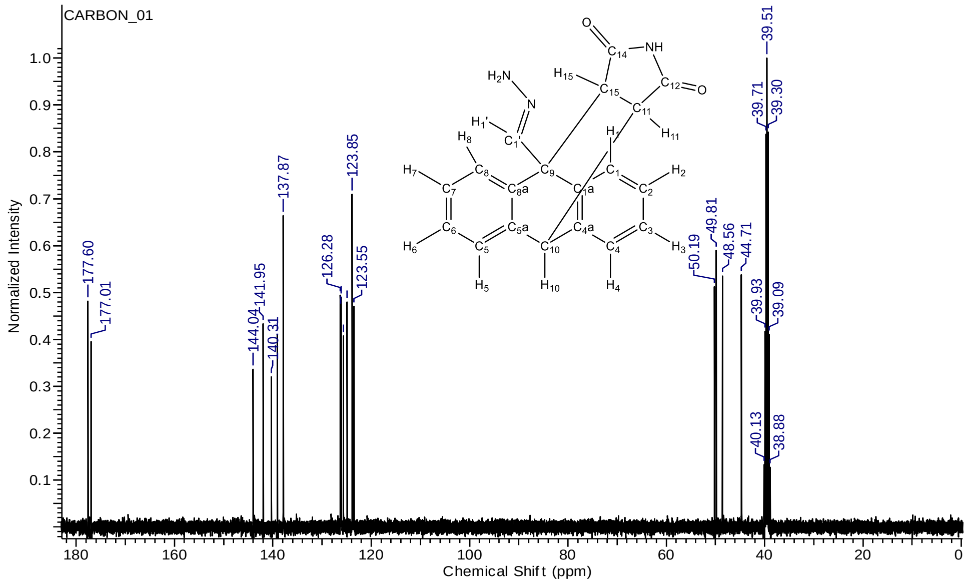

Figure S22. ^{13}C NMR spectrum of (*E*)-9-(hydrazonomethyl)-9,10-dihydro-9,10-[3,4]epipyrroloanthracene-12,14-dione (23g).

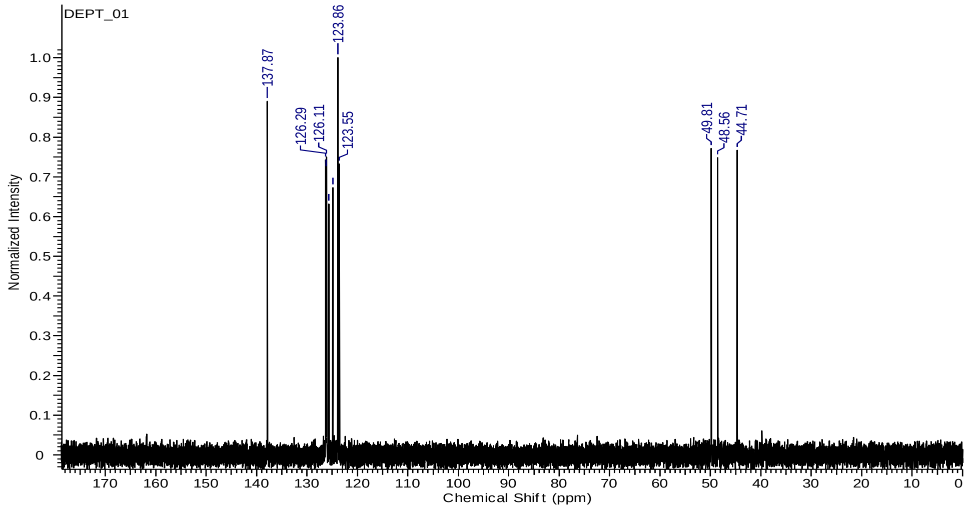

Figure S23. DEPT 90 NMR spectrum of (*E*)-9-(hydrazonomethyl)-9,10-dihydro-9,10-[3,4]epipyrroloanthracene-12,14-dione (23g).

Table S1. Tier-1 profiling screen of selected ethanoanthracenes.

Molecule	ID	ADMET Solubility [b]	ADMET Solubility Level [c]	ADMET BBB [d]	ADMET BBB Level [e]	ADMET EXT CYP2D6 Prediction	ADMET EXT Hepatotoxic Prediction
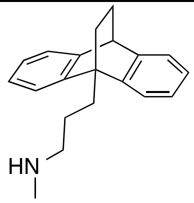	**8**	−5.2340	2	0.91500	0	true	true
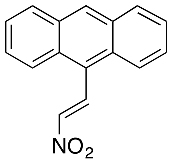	**12a**	−5.7050	2	0.40900	1	false	true
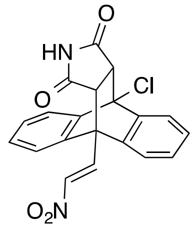	**13j**	−4.9690	2	−0.86900	3	false	true
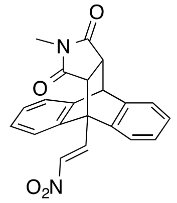	**13m**	−4.8360	2	−0.67600	3	false	true
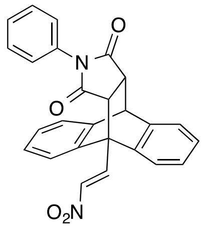	**16a**	−6.3790	1	−0.15900	2	false	true

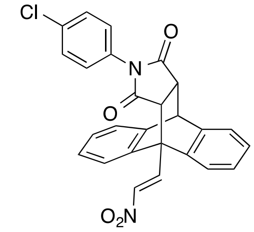	**16b**	−6.9710	1	0.047000	1	false	true
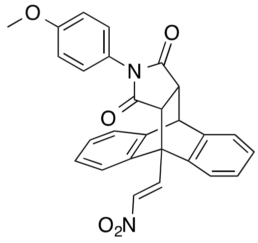	**16c**	−6.2330	1	-0.30500	2	false	true
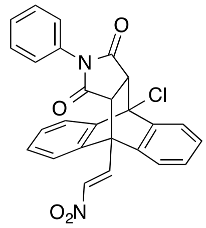	**16d**	−6.8310	1	0.037000	2	false	true
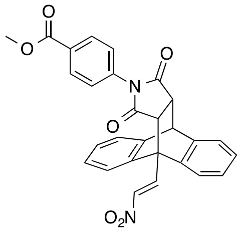	**16h**	−5.9530	2	-	4	false	false
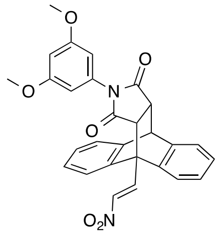	**16j**	−6.1270	1	-	4	false	true
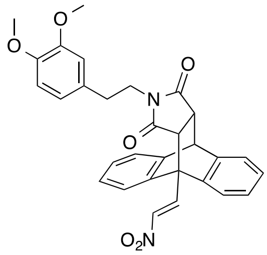	**17n**	−5.8600	2	-	4	false	true
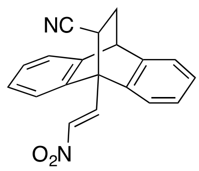	**19a**	−4.9590	2	−0.20100	2	false	true
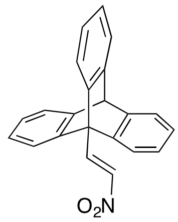	**20a**	−6.3320	1	0.52400	1	false	true

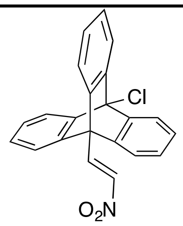	**20d**	−6.8050	1	0.64600	1	false	true

[a]Calculated using Pipeline Pilot Professional (v8.5.0.200) BIOVIA, Dassault Systèmes; [b]ADMET Solubility: Log of the water solubility at 25 °C (LogSw)(mol/L); [c]ADMET Solubility Level: Ranking of the solubility values into the following classes: 0: Extremely Low; 1: Very Low; 2: Low; 3: Good; 4: Optimal; 5: Very Soluble; [d]ADMET BBB: Predicts the blood brain barrier penetration of a molecule, defined as the ratio of the concentrations of solute (compound) on the both sides of the membrane after oral administration. [e]ADMET Blood Brain Barrier Absorption (BBB) Level: Ranking of LogBBB values into one of the following levels: 0: Very High; 1: High; 2: Medium; 3: Low; 4: Undefined (molecule is outside the confidence area of the regression model used to calculate LogBB).

Table S2. ADMET and Lipinski properties for selected ethanoanthracenes[a].

Molecule	ID	ADMET Absorption Level [b]	ADMET EXT PPB Prediction [c]	ALogP	Molecular Weight	Num HB A	Num HB D	Num Rot Bonds	Molecular Volume	Molecular Polar Surface Area
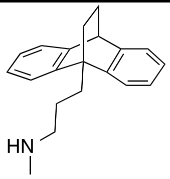	**8**	0	true	4.1160	277.40	1	1	4	201.68	12.030
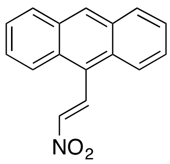	**12a**	0	true	4.0120	249.27	2	0	3	163.61	45.820
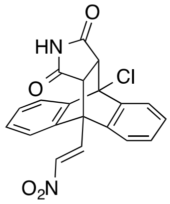	**13j**	0	false	2.1100	380.78	4	1	2	224.32	91.990
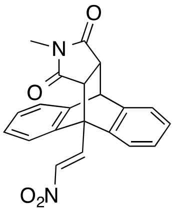	**13m**	0	false	1.9230	360.36	4	0	2	224.66	83.190
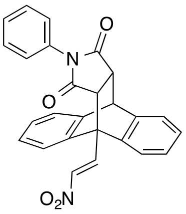	**16a**	0	true	3.4990	422.43	4	0	3	257.59	83.190

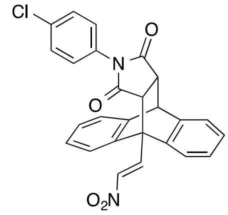	**16b**	0	true	4.1640	456.88	4	0	3	276.80	83.190
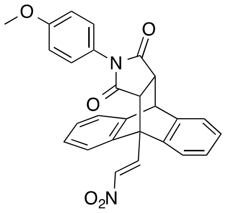	**16c**	0	true	3.4830	452.46	5	0	4	277.14	92.430
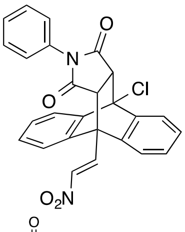	**16d**	0	true	3.8930	456.88	4	0	3	277.48	83.190
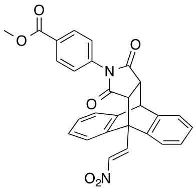	**16h**	1	true	3.3550	480.47	6	0	5	298.75	109.50
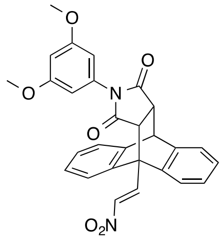	**16j**	0	true	3.4670	482.48	6	0	5	300.81	101.66
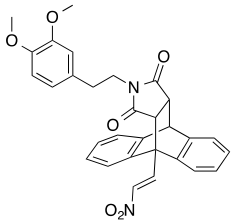	**17n**	0	true	3.7950	510.54	6	0	7	332.36	101.66
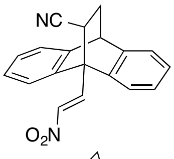	**19a**	0	true	3.2140	302.33	3	0	2	193.79	69.610
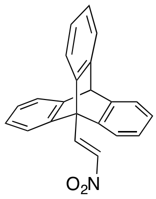	**20a**	0	true	4.3870	325.36	2	0	2	207.51	45.820
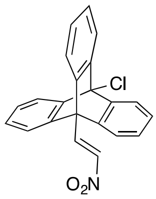	**20d**	0	true	4.7800	359.81	2	0	2	226.03	45.820

[a]Calculated using Pipeline Pilot Professional (v8.5.0.200) BIOVIA, Dassault Systèmes; [b]ADMET Absorption Level: Ranking of the molecule into one of the following levels: 0: Good; 1: Moderate; 2:

Poor; 3: Very Poor; [c]ADMET Plasma Protein Binding (PPB) Prediction: If true, the compound is predicted to be a binder (>=90%). Otherwise, it is predicted to be a weak or nonbinder(<90%).

References

1. Tawney, P.O.S.; R. H.; Conger, R. P.; Leibbrand, K. A.; Stiteler, C. H.; Williams, A. R. Maleimide and derivatives. II. Maleimide and N-methylolmaleimide. *J. Org. Chem.* **1961**, *26*, 15–21.
2. Kharitonov, Y.V.S.t.; E. E.; Shakirov, M. M.; Tolstikov, G. A. Synthetic Transformations of Higher Terpenoids: VIII. [4+2]-Cycloaddition Reactions of Lambertianic Acid. *Russ. J. Org. Chem.* **2003**, *39*, 57–74.
3. Kretov, A.E.K.c.; N. E. N-Arylmaleimides, their preparation, and properties. *Zh. Obshch. Khim.* **1956**, *26*, 221–225.
4. Roderick, W.R.B.; P. L. Action of trifluoroacetic anhydride on N-substituted amic acids. *J. Org. Chem.* **1963**, *28*, 2018–2024.
5. Wang, H.; Wei, J.; Jiang, X.; Yin, J. Novel polymerizable N-aromatic maleimides as free radical initiators for photopolymerization. *Polym. Int.* **2006**, *55*, 930–937.
6. Caulfield, M.J.; Solomon, D.H. Studies on polyimides: 2. Formation of high molecular weight poly(N-(hydroxyphenyl) maleimides). *Polym.* **1999**, *40*, 1251–1260.
7. Bastin, L. D.; Nigam, M.; Martinus, S.; Maloney, J. E; Benyack, L. L.; Gainer. B. Synthesis of substituted *N*-phenylmaleimides and use in a Diels–Alder reaction: a green multi-step synthesis for an undergraduate organic chemistry laboratory. *Green Chemistry Letters and Reviews*, **2019**, *12*, 127–135.
8. Manteca, I.E., B.; Ardeo, A.; Arrasate, S.; Osante, I.; Sotomayor, N.; Lete, E. Functionalized organolithium compounds: generation via reductive lithiation and nucleophilic addition to N-phenethylimides. Access to functionalized dihydropyrrolo[2,1-a]isoquinolinones. *Tetrahedron* **1998**, *54*, 12361–12378.
9. Kolyamshin, O.A.D.; V. A.; Kol'tsov, N. I. 4-(3-Dialkylamino-2,5-dioxopyrrolidin-1-yl)benzoic acid esters. *Russ. J. Org. Chem.* **2007**, *43*, 393–396.
10. Abblard, J.B., M. Imides substituted on the nitrogen atom. *Ger. Offen.* **1978**, *43*, 43.
11. Pal, B.P.; P. K.; Jaisankar, P.; Giri, V. S. First triphenylphosphine-promoted reduction of maleimides to succinimides. *Synthesis* 2003, 10, 1549–1552.
12. Matuszak, N.; Muccioli, G.G.; Labar, G. D.; Lambert, M. Synthesis and in Vitro Evaluation of N-Substituted Maleimide Derivatives as Selective Monoglyceride Lipase Inhibitors. *J. Med. Chem.* **2009,** *52*, 7410–7420.
13. Trujillo-Ferrara, J.;C.-B., Jose; Espinosa, J.; Garcia, J.; Martinez, F.; Miranda, R. Solvent-free synthesis of arylamides and arylimides, analogues of acetylcholine. *Synth. Commun.* **2005**, *35*, 2017–2023.
14. Tawney, P. O.; Snyder, R. H.; Conger, R. P.; Leibbrand, K. A.; Stiteler, C. H.; Williams, A. R. Maleimide and derivatives. II. Maleimide and N-methylolmaleimide, *J. Org. Chem.*, **1961**, *28*, 15–21.
15. Furdik, M.; Sutoris, V.; Drabek, J.; Pospisilova, S. Synergists in pyrethrum. III. N-Substituted 4-cyclohexene-1,2-dicarboximides and their 4-chloro derivatives. *Chem. Zvesti*. **1959**, *13*, 581–7.
16. Brunovlenskaya, I.I.; S., N.; Skvarchenko, V. R. Deamination of certain amines with amino group at the substituent in the head of a triptycene molecule bridge. *Dokl. Akad. Nauk SSSR* **1975**, *225*, 1323.
17. Yamamoto, G.; Kobayashi, Y.; Ono, K.; Yano, E.; Minoura, M.; Mazaki, Y. Synthesis and structures of 9-triptycylallene derivatives. *Bull. Chem. Soc. Jpn.* **2006**, *79*, 1293–1299.
18. Bartlett, P.D.; Greene, F.D. Triptycene 1-Carboxylic Acid and Related Compounds. The Decomposition of Ditriptoyl Peroxide. *J. Am. Chem. Soc.* **1954**, *76*, 1088–1096.
19. Nanu, I.B., V.; Secosan, E. Esters of 9,10-dihydroanthracene endo-α,β-succinic acid as models for the study of large-molecule plasticizers. *Bul. Stiint. Teh. Inst. Politeh. Timisoara* **1969**, *14*, 141–146.
20. Bova, S.S., S.; Rampa, A.; Gobbi, S.; Cima, L.; Fusi, F.; Sgaragli, G.; Cavalli, M.; de los Rios, C.; Striessnig, J.; Bisi, A. Anthracene Based Compounds as New L-type Ca2+ Channel Blockers: Design, Synthesis, and Full Biological Profile. *J. Med. Chem. 2009,* **52,** *1259–1262*.
21. Grossmann, G.; Potrzebowski, M.J.; Olejniczak, S.; Ziółkowska, N.E.; Bujacz, G.D.; Ciesielski, W.; Prezdo, W.; Nazarov, V.; Golovko, V. Structural studies of N-(2′-substituted phenyl)-9,10-dihydro-9,10-ethanoanthracene-11,12-dicarboximides by X-ray diffraction and NMR spectroscopy—proofs for CH/π interactions in liquid and solid phases. *New J. Chem.* **2003**, *27*, 1095–1101.
22. Yasmin, L.; Stubbs, K.A.; Raston, C.L. Vortex fluidic promoted Diels–Alder reactions in an aqueous medium. *Tetrahedron Lett.* **2014**, *55*, 2246–2248.
23. Alder, K.; K., Heimbach; The diene synthesis. XXXVII. The addition of anions of acrylic acid and butadiene-1-carboxylic acid. *Chem. Ber.* **1953**, *86*, 1312–1317.

24. Kiriazis, A.; Leikoski, T.; Mutikainen, I.; Yli-Kauhaluoma, J. The Diels-Alder Reaction between Deactivated Dienes and Electron-Deficient Dienophiles on Solid Support: Stereoselective Synthesis of Hexahydro-1,3-dioxoisoindoles. *J. Comb. Chem.* **2004**, *6*, 283–285.
25. Goh, Y.W.; White, J.M. Structure Correlation Study of Some Diels–Alder Cycloadducts of Anthracene. *Aust. J. Chem.* **2009**, *62*, 419–424.
26. Jiang, L.; Fu, Y.; Li, H.; Hu, W. Single-Crystalline, Size, and Orientation Controllable Nanowires and Ultralong Microwires of Organic Semiconductor with Strong Photoswitching Property. *J. Am. Chem. Soc.* **2008**, *130*, 3937–3941.
27. Hann, R.A. Use of dimethylformamide as a solvent for the knoevenagel reaction. *J. Chem. Soc. Perkin Trans. 1* **1974**, 1379.
28. Lock, G., Stach, K. Catalytic decomposition of hydrazones. I. Aromatic aldehyde hydrazones. *Ber. Dtsch. Chem. Ges. B* 1943, **76B**, 1252–1256.
29. Zhang, X.M.; Bordwell, F.G.; Bares, J.E.; Cheng, J.P.; Petrie, B.C. Homolytic bond dissociation energies of the acidic carbon-hydrogen bonds in .alpha.-substituted and 10-substituted 9-methylanthracenes and their related radical anions. *J. Org. Chem.* **1993**, *58*, 3051–3059.
